# DHPS‐Mediated Hypusination Regulates METTL3 Self‐m6A‐Methylation Modification to Promote Melanoma Proliferation and the Development of Novel Inhibitors

**DOI:** 10.1002/advs.202402450

**Published:** 2024-07-01

**Authors:** Jing‐si Guo, Jian Ma, Xi‐he Zhao, Ji‐fang Zhang, Kai‐li Liu, Long‐tian Li, Yu‐xi Qin, Fan‐hao Meng, Ling‐yan Jian, Yue‐hui Yang, Xin‐yang Li

**Affiliations:** ^1^ Department of Pharmacy Shengjing Hospital of China Medical University Shenyang 110004 P. R. China; ^2^ Department of Obstetrics and Gynecology Shengjing Hospital of China Medical University Shenyang 110004 P. R. China; ^3^ Department of Oncology Shengjing Hospital of China Medical University Shenyang 110004 P. R. China; ^4^ School of Pharmaceutical Engineering Jining Medical College University Park No.16 Haichuan Road, Gaoxin Jining Shandong 272000 P. R. China; ^5^ School of Pharmacy China Medical University Shenyang 110122 P. R. China

**Keywords:** DHPS, eIF5A‐Hypusine, m6A, melanoma

## Abstract

Discovering new treatments for melanoma will benefit human health. The mechanism by which deoxyhypusine synthase (DHPS) promotes melanoma development remains elucidated. Multi‐omics studies have revealed that DHPS regulates m6A modification and maintains mRNA stability in melanoma cells. Mechanistically, DHPS activates the hypusination of eukaryotic translation initiation factor 5A (eIF5A) to assist METTL3 localizing on its mRNA for m6A modification, then promoting METTL3 expression. Structure‐based design, synthesis, and activity screening yielded the hit compound **GL‐1** as a DHPS inhibitor. Notably, **GL‐1** directly inhibits DHPS binding to eIF5A, whereas **GC‐7** cannot. Based on the clarification of the mode of action of **GL‐1** on DHPS, it is found that **GL‐1** can promote the accumulation of intracellular Cu^2+^ to induce apoptosis, and antibody microarray analysis shows that **GL‐1** inhibits the expression of several cytokines. **GL‐1** shows promising antitumor activity with good bioavailability in a xenograft tumor model. These findings clarify the molecular mechanisms by which DHPS regulates melanoma proliferation and demonstrate the potential of **GL‐1** for clinical melanoma therapy.

## Graphical abstract

This study has innovatively identified DHPS‐mediated m6A methylation modification in melanoma and obtained a novel DHPS allosteric inhibitor, **GL‐1**, which has high efficiency, low toxicity, and good bioavailability in vivo and in vitro. These results provide a new reference for the pathogenesis of melanoma and the clinical treatment of melanoma.

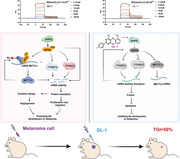

## 1 Introduction

Melanoma is a highly malignant form of cancer with increasing incidence and mortality rates.^[^ ^1^, ^2^, ^3^, ^4^, ^5^, ^6^ ^]^ Early surgical resection is the only effective treatment for malignant melanoma, and unfortunately, the prognosis is poor.^[^ ^7^, ^8^, ^9^ ^]^ Therefore, it is crucial to discover the development mechanism of melanoma and develop effective therapeutic drugs to improve patient outcomes.

Deoxyhypusine synthase (DHPS), as an intracellularly conserved enzyme, catalyzes the hypusination of eukaryotic translation initiation factor 5A (eIF5A).^[^ ^10^, ^11^, ^12^ ^]^ Our team was the first to report the inhibitory effect of DHPS inhibitors on melanoma.^[^ ^13^ ^]^ In recent years, it has been found that DHPS is highly expressed in some cancers (e.g., colorectal cancer, leukemia) and promotes the protein translation process by activating eIF5A (eIF5A‐Hyp), which maintains the hyperproliferation of cancer cells.^[^ ^14^, ^15^, ^16^ ^]^ eIF5A promotes peptide chain elongation and release by occupying the E‐site of the ribosome, thus regulating the translation process.^[^ ^17^ ^]^ However, not all proteins require eIF5A regulation, and the expression level of DHPS varies in different cancer cells.^[^ ^13^, ^14^, ^15^, ^16^ ^]^ Therefore, discovering the regulatory mechanism of DHPS/eIF5A in melanoma is an essential basis for clinical chemotherapy.

Since DHPS regulates the protein translation process of mRNAs by promoting the hypusination of eIF5A, it remains to be elucidated whether this process is associated with epitope modification. N6‐methyladenosine (m6A) is a dynamic and reversible methylation modification at the N6 site of adenosine that is important for maintaining the dynamic equilibrium of mRNA.^[^ ^18^, ^19^, ^20^ ^]^ There have been limited studies on the role of m6A‐related regulatory proteins in melanoma, including “writers,” “erasers,” and “readers.”^[^ ^21^, ^22^, ^23^, ^24^, ^25^ ^]^ Methyltransferase‐like 3 (METTL3) of the “Writers” plays a vital role in promoting melanoma tumorigenesis;^[^ ^26^, ^27^, ^28^ ^]^ obesity‐associated protein (FTO) in “Erasers”^[^ ^29^ ^]^ acts as an, as an m6A demethylase, plays a crucial role in promoting melanoma tumorigenesis and anti‐PD‐1 resistance.^[^ ^29^, ^30^ ^]^ Additionally, YTH domain family 2 (YTHDF2) of the “Readers” promotes the development of ocular melanoma by reducing mRNA degradation through reduced m6A recognition.^[^ ^31^ ^]^ Thus, studying the regulatory relationship the study of the regulatory relationship between DHPS/eIF5A‐Hyp and m6A modification will provide a theoretical basis for the clinical application of DHPS in the treatment of melanoma and serve as a mechanistic reference for developing novel DHPS inhibitors.

Here, we aimed to uncover the pro‐oncogenic mechanism of DHPS in melanoma by mediating the hypusination of eIF5A to promote the m6A modification of METTL3 itself through a multi‐omics approach to elucidate the value of DHPS as a therapeutic target for melanoma. Based on the oncogenic mechanism of DHPS, a series of novel DHPS allosteric inhibitors were obtained through structure‐based compound design and synthesis. The screening of the hit compound (**GL‐1**) at the enzyme and cellular levels, in vivo studies were conducted to determine the ability of **GL‐1** to target DHPS and its efficacy against melanoma.

## 2 Result

### 2.1 Discovery and Validation of DHPS as a Target in Skin Melanoma

**Figure 1:**
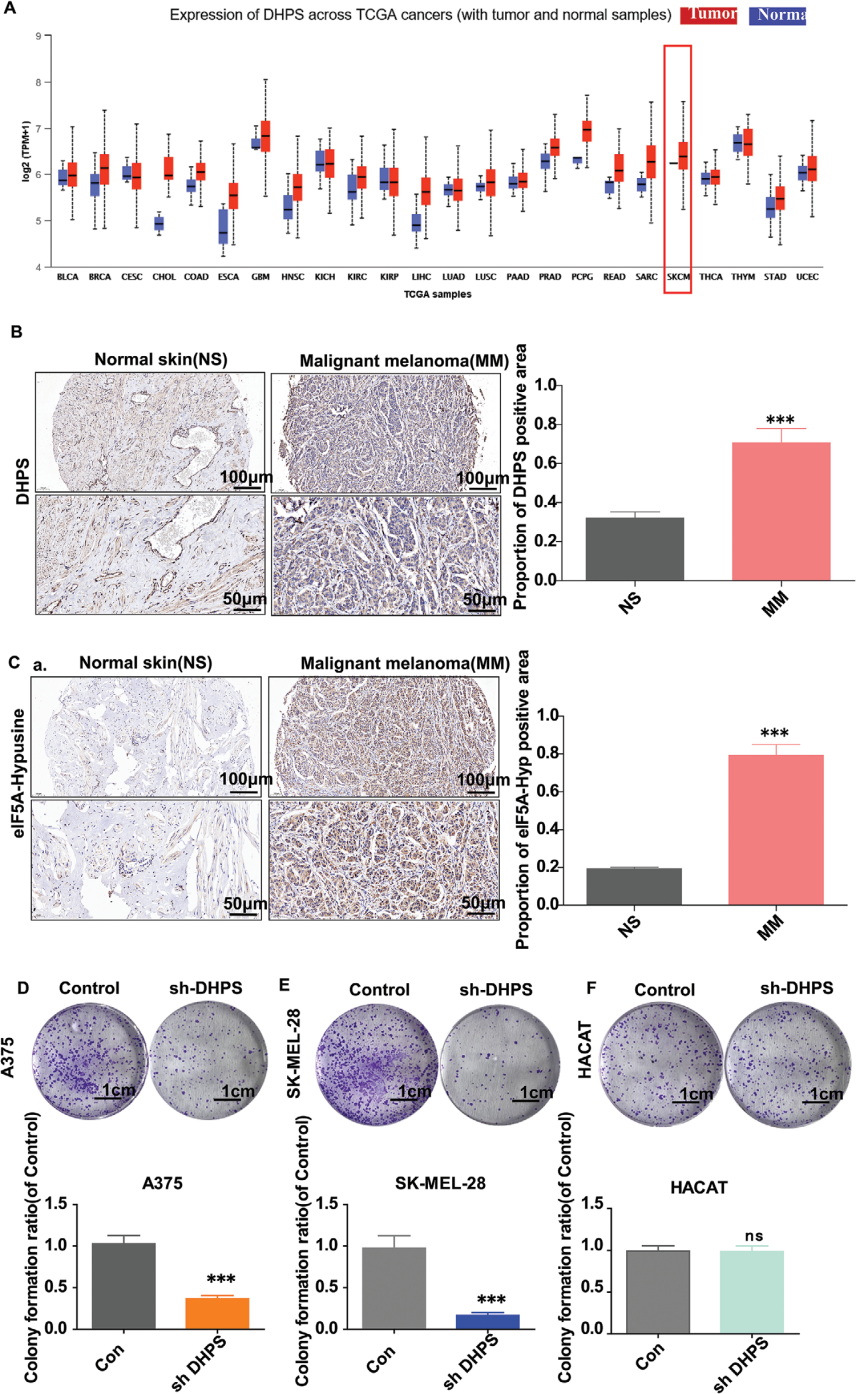
Identification of DHPS as a potential differential target in skin melanoma. A) Bioassay analysis of DHPS shows that DHPS is specifically highly expressed in a variety of cancer tissues, particularly skin cancer. B) Human melanoma microarray results show that DHPS is highly expressed in melanoma and low in healthy tissues. (Scale bar = 100 µm/50 µm, *n* = 129, ^***^ *p* < 0.001) C) Human melanoma microarray results show that hypusination is high in melanoma and low in healthy tissues. (Scale bar = 100 µm/50 µm, *n* = 129 ^***^ *p* < 0.001) D–F) Knockdown of DHPS effectively inhibits melanoma proliferation but does not affect normal skin cells (Scale bar = 1 cm, *n* = 3, ^***^ *p* < 0.001).

Analysis of The Cancer Genome Atlas (TCGA) database revealed that DHPS is highly expressed in cancer tissues, particularly in skin cancer (**Figure** **1**A).^[^ ^32^ ^]^ Furthermore, a human melanoma tissue chip (111 melanoma samples and 18 adjacent normal tissues) was used for analysis. It was found that DHPS is more highly expressed in melanoma than in normal skin tissue (Figure 1B). DHPS‐mediated hypusination modifications were also differentially expressed in melanoma compared to normal skin tissue (Figure 1C). When the DHPS gene was knocked down, the proliferation of melanoma cells was inhibited. In contrast, the proliferative ability of normal skin cells was unaffected (Figure 1D).

### 2.2 DHPS Affects the Stability of mRNA by Regulating m6A Modifications in Melanoma

**Figure 2:**
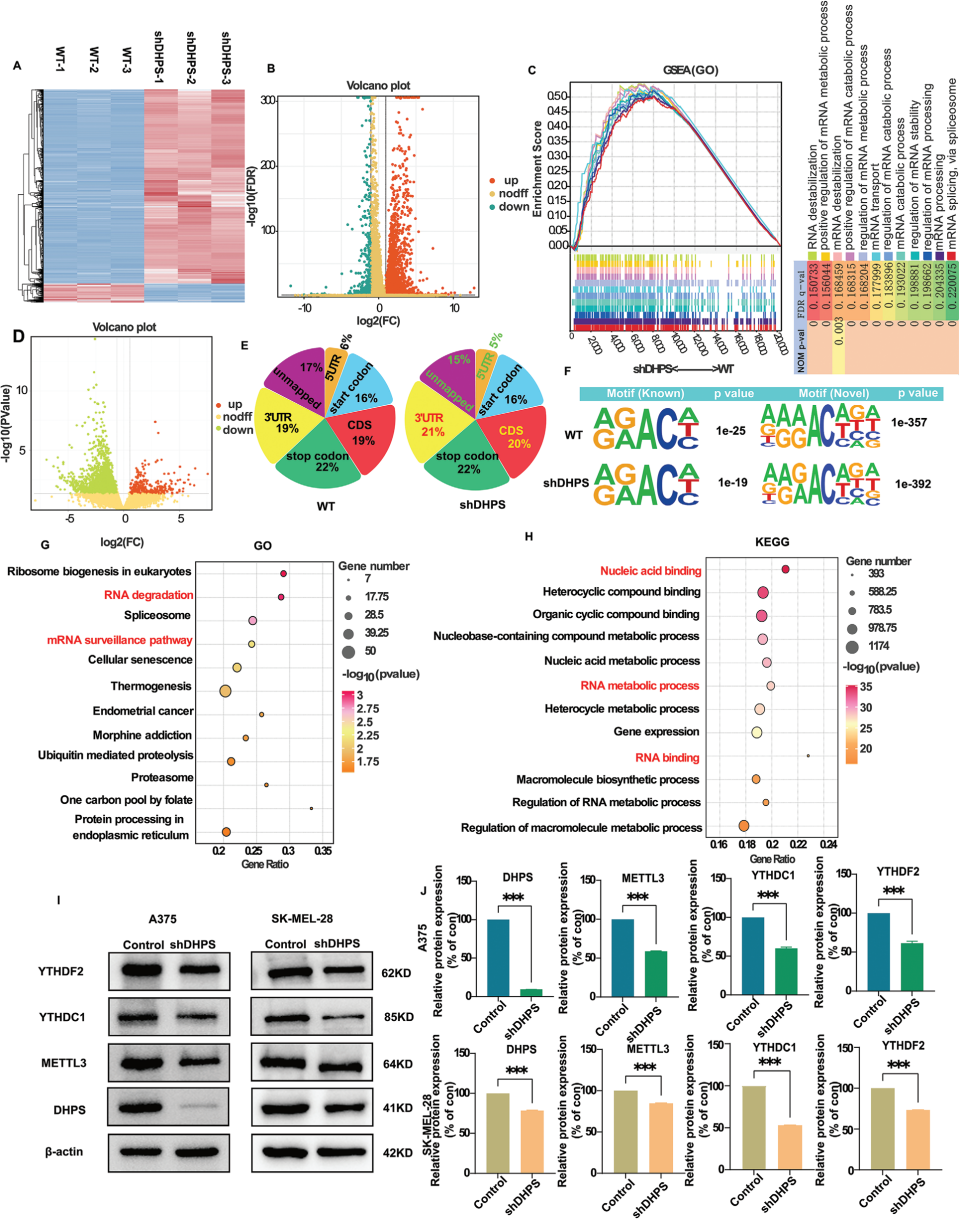
DHPS maintains mRNA stability by m6A methylation modification in melanoma cells. A) The heat map analysis, hierarchical clustering, and volcano plot represent a differential analysis B) of the A375 cell (WT) and DHPS knockdown A375 cell (shDHPS) genes. Color scale: Red is higher expressed; blue is lower expressed. C) The mRNA metabolism and stability‐related pathways were positively associated with low DHPS expression by GSEA (P < 0.05, FDR < 0.25). D) Volcano plot of m6A peaks detected by MeRIP‐seq in the A375‐WT and the A375‐shDHPS. Red dots mean m6A peaks up, while green dots mean m6A peaks down. Note that multiple peaks may map to the same gene. E) Peak distribution of m6A modification in MeRIP‐seq results. F) The sequence motif identified from the sequencing profile. G) KEGG and GO analysis H) of genes with high m6A levels in the A375‐WT and A375‐shDHPS cells. I,J) Protein expression levels of YTHDF2, YTHDC1, and METTL3 in A375 cells after DHPS knockdown. (Treatment group vs control group, ^*^ *p* < 0.05, ^**^ *p* < 0.01, ^***^ *p* < 0.001).

To explore the mechanism by which DHPS regulates melanoma development, high‐throughput sequencing of melanoma cells after knockdown of the DHPS gene was performed here using mRNA‐seq. After the knockdown of the DHPS gene, the mRNA expression of 4750 genes was up‐regulated, and 466 genes were down‐regulated in A375 cells (**Figure** **2**A,B). GESA analysis revealed that the differential genes mainly focused on mRNA stability pathways (e.g., silencing, degradation, and metabolism) (Figure 2C). However, the colony formation assay in Figure 1D suggests that DHPS is an oncogene contrary to the mRNA overexpression shown by the high‐throughput sequencing results. Thus, the phenomenon of mRNA overexpression in melanoma cells with proliferation inhibition suggests that the regulatory mechanism of DHPS may be related to the stability mechanism of mRNA.

The m6A modification is a crucial factor that affects mRNA stability. To understand whether DHPS is associated with the m6A modification mechanism of mRNAs, a high‐throughput analysis of m6A methylation levels was performed on knockdown DHPS and normal A375 cells. The results of MeRIP‐seq showed that in A375 cells with the DHPS gene knockdown, the m6A peak abundance of 2551 genes decreased, while that of 246 genes increased (Figure 2D). Subsequent investigation on the m6A peak distributions revealed that total m6A distribution patterns were the same in the control groups and DHPS knockdown groups (Figure 2E). The consensus motif “RRACH,” which is highly concentrated at m6A sites, was present in both the control and DHPS knockdown cells (Figure 2F).^[^ ^33^ ^]^ Enrichment analysis showed that the regulation of melanoma cell signaling pathways by DHPS may be related to RNA stability (Figure 2G,H). Additionally, western blot analysis revealed that the expression of three vital proteins that regulate m6A modification, namely METTL3, YTHDF2, and YTHDC1, was reduced in melanoma cells after DHPS knockdown (Figure 2I,J).

These findings suggest that DHPS may drive melanoma by modifying the gene through m6A methylation.

### 2.3 DHPS Regulates the Self‐m6A‐Methylation of METTL3 Through the Catalysis of the Hypusination of eIF5A to Maintain mRNA Stability

**Figure 3:**
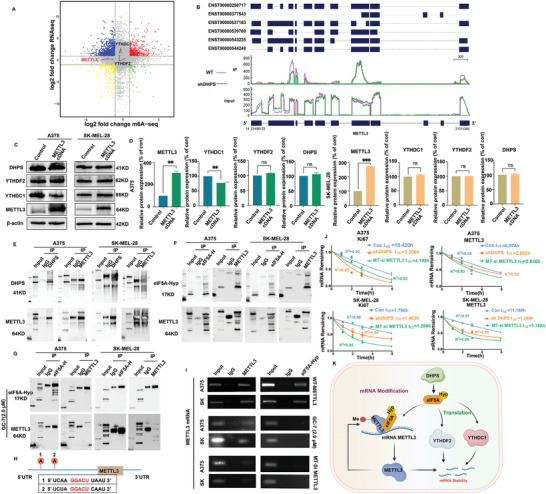
DHPS regulates mRNA stability and YTHDC1, and YTHDF2 protein expression in melanoma cells through eIF5A‐Hyp‐mediated METTL3 self‐m6A‐methylation modification. A) Nine‐quadrant plot of A375‐WT compared to A375‐shDHPS. The horizontal axis is the multiplicity of difference in peak abundance values of m6A (taken as log2), the vertical axis is the multiplicity of difference in gene expression of the transcriptome (taken as log2), and the dashed lines on the horizontal and vertical axes are the default thresholds for screening for differential genes/peak in the two cohorts |log2FC| > 1. Grey dots indicate nondifferentiated genes, red and yellow dots indicate genes and m6A peak abundance values trend consistently up/down, and blue and green dots indicate the opposite trend of base gene and m6A peak abundance values. B) The m6A abundance of METTL3 transcripts in A375‐shDHPS cells compared to A375‐WT cells. C,D) Protein expression levels of YTHDF2, YTHDC1, and METTL3 in A375 or SK‐MEL‐28 cells after METTL3 overexpression. (Treatment group versus control group, ^**^ *p* < 0.01, ^***^ *p* < 0.001). The interaction between DHPS and METTL3 E) or eIF5A‐Hyp and METTL3 F) was studied in the Co‐IP assay. G) Co‐IP assay for inhibition of eIF5A‐Hyp interaction with METTL3 after **GC‐7** (2 µm) treatment in melanoma cells A375 and SK‐MEL‐28 cells. H) The m6A peak region of METTL3 uncovered the presence of two similar “RRACH” sequences in the CDS region near the 5′UTR. I) RIP assays of METTL3 mRNA binding to eIF5A‐Hyp or METTL3. Interference with METTL3 and **GC‐7** (2.0 µm) inhibited the binding of METTL3 mRNA to eIF5A‐Hyp or METTL3. J) (t_1/2_) of Ki67 and METTL3 in A375 and SK‐MEL‐28 cells. K) Schematic representation of the mechanism by which DHPS regulates mRNA stability in melanoma cells.

To identify critical downstream targets of DHPS‐mediated m6A modifications in A375 cells, we analyzed the frequency and distribution of m6A modifications in differentially expressed mRNA by MeRIP‐seq and RNA‐seq tandem analysis. This involved the selection of mRNAs with m6A peaks and mRNA abundance changes of >1.0‐fold at *p* < 0.05. Further analysis revealed that the methylation of METTL3 itself was downregulated, with no significant difference in mRNA content. The analysis of the m6A methylation site of METTL3 also revealed that the abundance of its 3′UTR and CDS region was reduced significantly (**Figure** **3**A,B). Meanwhile, the mRNA expression levels of m6A modification of YTHDF2 or YTHDC1 were not significantly different (Figure 3A). Consequently, we postulated that the regulatory mechanism of DHPS on METTL3 might be distinct from that of DHPS on YTHDC1 or YTHDF2. To ascertain whether the regulatory effect of DHPS on YTHDC1 or YTHDF2 involves the participation of METTL3, the expression levels of DHPS, YTHDC1, and YTHDF2 proteins were analyzed following overexpression of METTL3 in melanoma cells. Further overexpression of the METTL3 protein resulted in no significant changes in DHPS, YTHDC1, and YTHDF2 proteins, as shown in Figure 3C,D. These results indicated that DHPS may regulate the mRNA self‐m6A‐methylation modification of METTL3. It was determined that the translational regulation of YTHDF2 and YTHDC1 by DHPS did not involve m6A‐methylation modification.

Considering METTL3's role as a methyltransferase, we hypothesized that METTL3's m6A modification might be “written” by itself and that DHPS or eIF5A‐Hyp could mediate this “writing” process. To investigate this, we conducted Co‐IP and RIP assays to determine whether DHPS or eIF5A‐Hyp regulates the m6A modification of METTL3. The Co‐IP assays revealed that DHPS could not directly bind to METTL3 in melanoma cells, while eIF5A‐Hyp could bind to METTL3. Interestingly, this binding was hindered when the hypusination of eIF5A was inhibited (Figure 3E–G). Additionally, analysis of the m6A peak region of METTL3 uncovered the presence of two similar “RRACH” sequences in the CDS region near the 5′UTR (Figure 3H). Based on this discovery, we designed specific RNA primers for RIP experiments (for details, Scheme S1, Supporting Information), and the results showed that METTL3 could bind to its mRNA in melanoma and eIF5A‐Hyp could also bind to the METTL3 mRNA (Figure 3I). However, this binding was disrupted when the hypusination of eIF5A was inhibited. Furthermore, neither eIF5A‐Hyp nor METTL3 could bind to the METTL3 mRNA after the mutation of A to C in the “RRACH” (Figure 3I). Moreover, the knockdown of DHPS and the mutation of the m6A site of METTL3 resulted in reduced mRNA stability of Ki67 and METTL3 (Figure 3J).

Taken together, these results suggest that DHPS mediates the hypusination of eIF5A by assisting METTL3 in recognizing its m6A site for methylation modification and maintaining the stability of mRNAs in melanoma cells (Figure 3H).

### 2.4 Discovery of GL‐1 as a Novel DHPS Inhibitor

**Figure 4:**
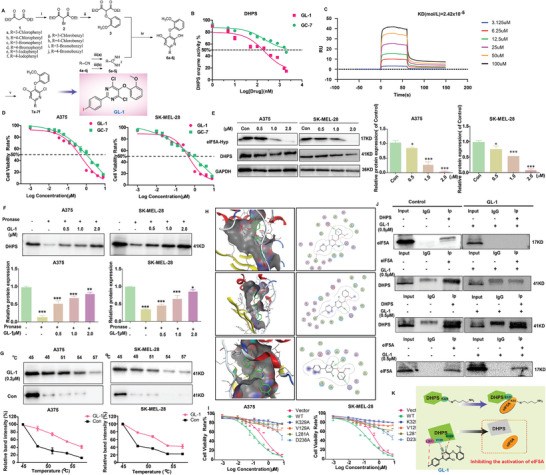
Synthesis and targeting studies of GL‐1 as a novel DHPS inhibitor. A) Synthesis Scheme: Reagents and conditions: i) NBS, H_2_SO_4_, CHCl_3_, 50 °C, 8 h; ii) guaiacol, K_2_CO_3_, acetonitrile, 60 °C, overnight; iii‐a) Na, CH_3_OH, CH_2_Cl_2_, rt, 48 h; NH_4_Cl, rt, overnight; or iii‐b) ethanol, acetyl chloride, CH_2_Cl_2,_ rt, 24 h; Na, NH_4_Cl, CH_3_OH, rt, overnight; iv) Na, CH_3_OH, rt, 24 h; v) POCl_3_, 100 °C, overnight. B) The inhibitory effects of **GL‐1** and **GC‐7** on DHPS enzyme activity were compared at the same concentrations (1, 5, 10, 50, 100, 200, 400, 800, 1600, and 2000 nm). C) SPR analysis of the kinetic interactions between the **GL‐1** and DHPS. D) The inhibitory effects on A375 and SK‐MEL‐28 cell viability were measured after treatment with **GL‐1** or **GC‐7** at different concentrations (0.001, 0.01, 0.02, 0.035, 0.0625, 0.125, 0.25, 0.50, 1.00, 2.00, 4.00, and 8.00 µm) for 24 h. E) Effect of different concentrations (0, 0.5, 1.0, 2.0 µm) of **GL‐1** on hypusiantion protein expression. F,G) Western blot analysis of DARTS and CETSA samples demonstrated that **GL‐1** and DHPS were able to bind stably. H) Docking pose of **GL‐1** within the allosteric site of DHPS (PDB code: 6PGR). I) Cell viability rates after transfection of empty vector plasmids, DHPS‐WT plasmid, DHPS‐K329A plasmid, DHPS‐V129A plasmid, DHPS‐L281A plasmid, and DHPS‐D238A plasmid into A375 and SK‐MEL‐28 cells for 48 h and addition of different concentrations of **GL‐1** for 24 h of continuous action. J) Co‐IP results showed that **GL‐1** could block the binding of DHPS to the eIF5A‐Hyp protein. (Treatment group vs control group, ^*^ *p* < 0.05, ^**^ *p* < 0.01, ^***^ *p* < 0.001).

Based on the new mechanism of DHPS regulation in melanoma, the target potential of DHPS has been identified, which makes the development of DHPS‐targeted inhibitors of great significance. A series of DHPS allosteric inhibitors with novel structures were designed by fragment‐based structure optimization (Scheme S2, Supporting Information). A total of 14 target compounds were obtained by directed synthesis (**Figure** **4**A). Structural confirmation of the target compounds is detailed in the Supporting Information (Scheme S3, Figures S1–S48, and Tables S1–S16). DHPS enzyme assay and CCK‐8 assay screening (Table S17, Supporting Information) were used to obtain the hit compound **7f** (**GL‐1**). **GL‐1** inhibited DHPS enzyme activity more than **GC‐7** (IC_50_ **_GL‐1_**  = 0.21 ± 0.07 µm, IC_50_ **_GC‐7_**  = 1.58 ± 0.02 µm) and has a high affinity for DHPS enzymes (KD** _GL‐1_**  = 2.42 × 10^−5^, KD **_GC‐7_**  = 3.11 × 10^−5^) (Figure 4B,C; Figure S49, Supporting Information). Notably, the ability of **GL‐1** to inhibit cell viability at the same concentration was more prominent compared to **GC‐7**, especially for A375 cells (Figure 4D). Moreover, **GL‐1** inhibited hypusination of eIF5A in a concentration‐dependent manner (Figure 4E).

The ability of **GL‐1** to stably bind to DHPS in cells was confirmed by CETSA and DARTS assays (Figure 4F,G). Docking studies revealed that **GL‐1** could interact with amino acid residues in the active pocket of DHPS in three poses (K329, V129, L281, and D238). A single‐point mutation of the DHPS protein was performed, and the results showed that the interaction of **GL‐1** with all four amino acid residues inhibited the cell viability of either A375 or SK‐MEL‐28 cells. The D238 site was first identified as the critical amino acid site for DHPS activity, although its role was slightly weaker.

Simultaneously, since DHPS can directly bind to eIF5A to exert hypusination function, the Co‐IP assay was used to detect the binding ability of DHPS to eIF5A after **GL‐1** treatment. The results were shown in Figure 4J, **GL‐1** could inhibit the binding of DHPS to eIF5A. However, **GC‐7** could not inhibit the binding of DHPS to eIF5A (Figure S50, Supporting Information).

The above results indicate that **GL‐1** is a potential DHPS inhibitor that could inhibit eIF5A's hypusination by directly interacting with DHPS (Figure 4K).

### 2.5 GL‐1 Exhibits Promising Antimelanoma Efficacy In Vitro

**Figure 5:**
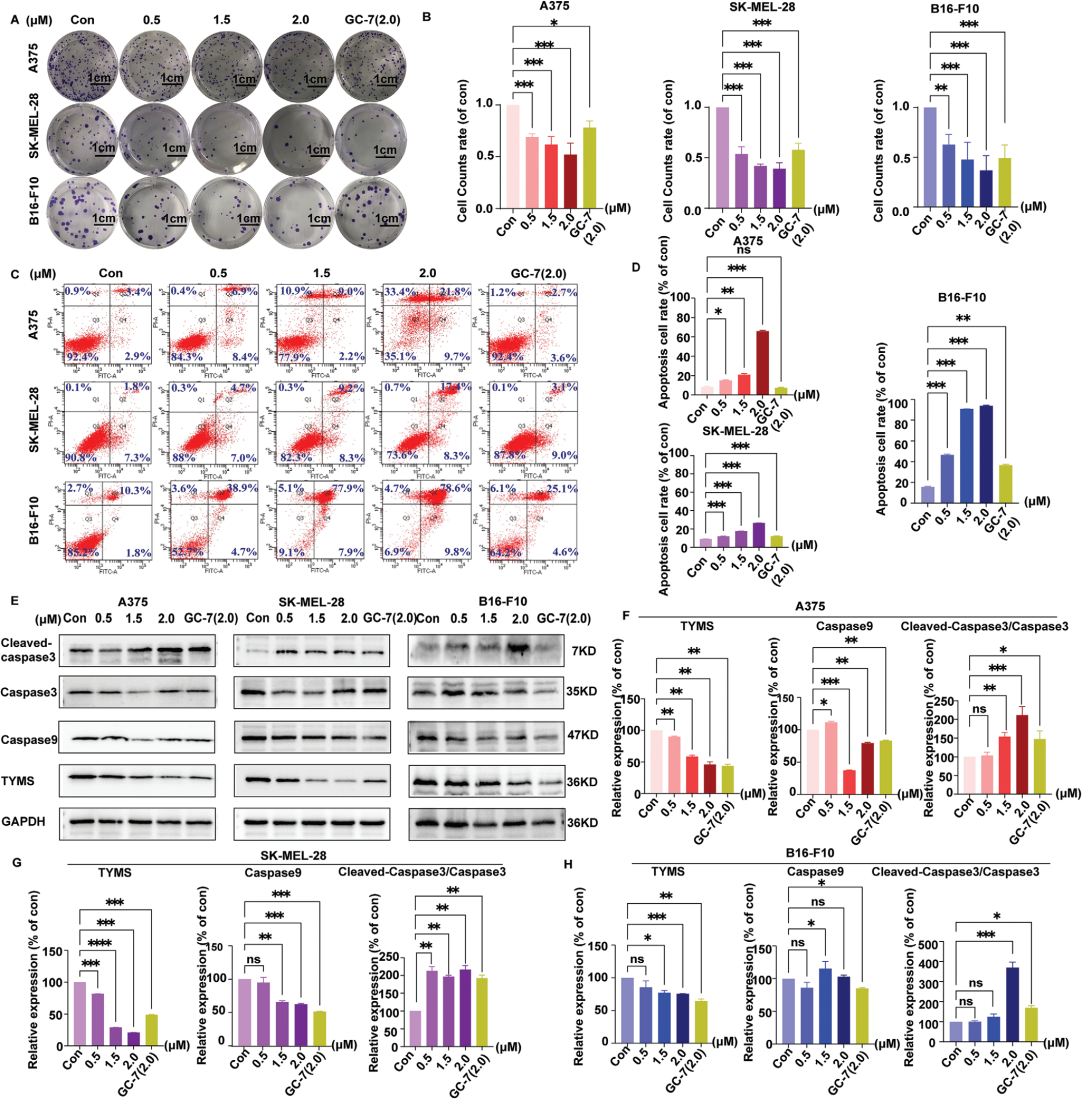
GL‐1 promotes melanoma apoptosis in a concentration‐dependent manner. A,B) GL‐1 could inhibit colony formation in a concentration‐dependent manner (Scale bar = 1 cm). C,D) FlowJ assay the induction of apoptosis in melanoma cells by GL‐1 and GC‐7. E–H) Western blot analysis of GL‐1 and GC‐7 on TYMS, Caspase9, and cleaved‐caspase3, caspase3 proteins from tumor tissue. (Treatment group vs control group, ^*^ *p* < 0.05, ^**^ *p* < 0.01, ^***^ *p* < 0.001).

To verify the antiproliferative ability of **GL‐1**, we conducted a colony formation assay to explore the effect of **GL‐1** on the proliferative ability of melanoma cells. The results, shown in **Figure** **5**A,B, indicate that **GL‐1** has a concentration‐dependent inhibitory effect on the proliferation of A375, SK‐MEL‐28, and B16‐F10 cells. Furthermore, **GL‐1** was more effective than **GC‐7** at the same concentration (2.0 µm).

Flow cytometry analysis was utilized to detect apoptosis induced by **GL‐1** in melanoma cells (Figure 5C,D). However, **GL‐1** had a significantly stronger regulatory effect on B16‐F10 than on A375 and SK‐MEL‐28. In A375 cells, treatment with **GL‐1** at a concentration of 2.0 µm for 24 h resulted in 35.1% cell survival, while **GC‐7** showed 92.4% survival at the same concentration. Western blot analysis revealed that **GL‐1** inhibited the expression of TYMS protein in a concentration‐dependent manner in A375 or SK‐MEL‐28 cells. Similar results were observed in B16‐F10 cells (Figure 5E–H). Additionally, **GL‐1** inhibited the expression of caspase9 protein and induced the activation of caspase3.

In summary, **GL‐1** effectively inhibited the proliferation of melanoma cells by inhibiting the expression of the proliferative protein TYMS and affecting the content of caspase9 and the activation of caspase3.

### 2.6 GL‐1 Destroyed Cu^2+^ Homeostasis and Inhibited Melanoma Cells from Secreting a Variety of Cytokines

**Figure 6:**
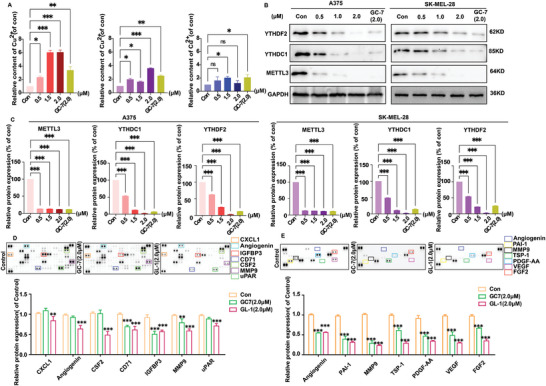
Other mechanisms of anticancer effects of GL‐1 on melanoma cells. A) Changes in Cu^2+^ content after 24 h of intracellular GL‐1 action. B,C) Effects of GL‐1 and GC‐7 on METTL3, YTHDF2, and YTHDC1 protein expression. The effect of GL‐1 (2.0 µm) or GC‐7 (2.0 µm) on human multiple cytokines D) and angiogenesis‐related proteins E) in A375 cells was analyzed, and the average spot pixel density on the array membrane was quantified using ImageJ software to generate histogram profiles of selected analyses. (Treatment group vs control group, ^*^ *p* < 0.05, ^**^ *p* < 0.01, ^***^ *p* < 0.001).

However, **GL‐1** did not regulate the protein expression of caspase9 and activated caspase3 in A375, SK‐MEL‐28, and B16‐F10 cells in a concentration‐dependent manner, suggesting that other apoptosis‐related regulatory mechanisms may exist. In recent years, metal ions have been shown to play an important role in cell proliferation and apoptosis. We are concerned that copper ion homeostasis may directly influence apoptotic signaling in melanoma. To study the impact of **GL‐1** on copper ion homeostasis in melanoma cells, ELISA was used to quantify intracellular Cu^2+^ levels. The results showed that **GL‐1** could disrupt intracellular Cu^2+^ homeostasis in A375 and SK‐MEL‐28 cells in a concentration‐dependent manner, resulting in large amounts of Cu^2+^ retained in the cells (**Figure** **6**A). However, this effect was not evident for B16‐F10 cells.

The study investigated the effect of **GL‐1** on the protein expression of METTL3, YTHDF2, and YTHDC1, which are regulated by DHPS. The results indicated that **GL‐1** inhibited the protein expression of these three proteins in a concentration‐dependent manner in A375 and SK‐MEL‐28 cells (Figure 6B,C), consistent with the findings of DHPS knockdown. Cu^2+^ plays a role in several cancer‐related processes, such as generating and transporting cancer‐related cytokines. Here, we used proteomics to investigate the relevant mechanisms of DHPS and cytokine regulation. Solid‐state antibody chip results, including 105 antibodies, showed that **GC‐7** and **GL‐1** affected the expression of melanoma cytokines, among which ANG, IGFBP3, CSF2, CD71, CXCL1, and MMP9 were the most significant(Figure 6D; Figure S51, Supporting Information). IGFBP3, CSF2, CD71, and MMP9 are all factors associated with cell proliferation and migration. This suggests that when DHPS is inhibited, it directly affects the metastasis of melanoma cells. Further scratch assay also confirmed the ability of **GL‐1** to inhibit cell migration in a concentration‐dependent manner (Figure S52, Supporting Information).

Since ANG, as an angiogenesis factor, directly affects melanoma angiogenesis, we used angiogenesis antibody microarrays containing 55 antibodies to explore the anti‐angiogenic effects of **GL‐1** and **GC‐7**. The results showed that **GL‐1** and **GC‐7** effectively inhibited the expression of ANG, PAI‐1, MMP9, TSP‐1, PDGF, VEGF, and FGF2 factors. The result suggested that inhibition of DHPS activity interferes with the expression of cytokines, particularly angiogenesis factors, and may disrupt the angiogenesis pathway.

### 2.7 GL‐1 Effectively Inhibits Melanoma Development In Vivo and Shows Favorable Bioavailability

**Figure 7:**
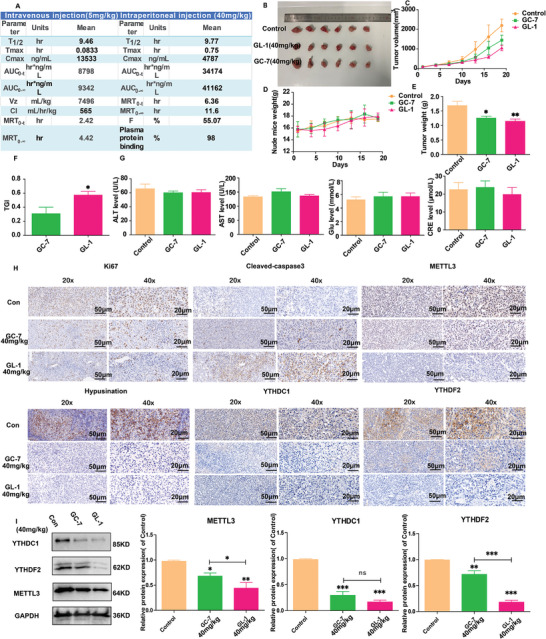
Studies of the in vivo pharmacokinetics and anti‐melanoma activity of GL‐1. A) Evaluation of the pharmacokinetics of **GL‐1**. B–E) A375 cell xenograft mice treated with SPSS, **GC‐7**(40 mg kg^−1^), and **GL‐1**(40 mg kg^−1^) respectively shown in image B), tumor volume C), weight change D), and tumor weight E) analysis. *n* = 6 mice per group. F) TGI of **GL‐1** versus **GC‐7**. G) Blood biochemistry of **GL‐1**or **GC‐7** for ALT, AST, CRE, and blood glucose level. H) IHC analysis of **GL‐1** and **GC‐7** on Ki67, Hypusination, Cleaved‐caspase3, METTL3, YTHDF2, and YTHDC1 protein expression in tumor tissue. I) Western blot analysis of **GL‐1** and **GC‐7** on METTL3, YTHDF2, and YTHDC1 protein expression in tumor tissue. (Scale bar = 50 µm/20 µm, ^*^ *p* < 0.05, ^**^ *p* < 0.01, ^***^ *p* < 0.001).

A pharmacokinetic study was conducted to determine the drug‐gable characteristics of **GL‐1**. Blood concentrations were analyzed at various time points following intravenous injection of 5 mg kg^−1^ and intraperitoneal injection of 40 mg kg^−1^. The results showed that **GL‐1** is stable and has high bioavailability (F = 55.07%) in vivo (**Figure** **7**A). Since there was no significant difference in the in vivo half‐life between intravenous and intraperitoneal administration (T_1/2_, i.v. = 9.46 h, T_1/2_, i.p. = 9.77 h), we chose the milder intraperitoneal administration for the in vivo pharmacodynamic study based on animal welfare considerations.

A xenograft mouse model was constructed using A375 cells to verify the role of **GL‐1** as a DHPS inhibitor against melanoma in vivo. The inhibitory effect of **GL‐1** on melanoma tissues was investigated at the same dose (40 mg kg^−1^) using **GC‐7** as a positive reference. The results are shown in Figure 7B–E; after 18 days of administration at 3‐day intervals, **GL‐1** effectively inhibited the proliferation of tumor tissues with a TGI of 59% while having less effect on the body weight of the model mice (Figure 7F). Moreover, there was no significant difference in the effects on liver and kidney function (Figure 7G). The low toxicity of **GL‐1** was also confirmed by HE stains (Figure S54, Supporting Information). In addition, IHC analysis of tumor tissues showed that **GL‐1** inhibited the expression of Ki67, promoted the expression of cleaved‐caspase3, and inhibited hypusiantion in tumor tissues (Figure 7H). The IHC and western blot results showed that both** GL‐1** and **GC‐7,** as DHPS inhibitors, inhibited METTL3, YTHDF2, and YTHDC1 in tumor tissues (Figure 7H,I).

## 3 Discussion

Melanoma's high degree of malignancy results in a low survival rate due to the lack of effective treatments. The discovery of new therapeutic mechanisms and targeted drugs is crucial to bring hope for patients' survival. DHPS has received extensive attention in recent years.^[^ ^34^, ^35^ ^]^ DHPS catalyzes the hypusination of eIF5A, an essential regulator of protein translation.^[^ ^36^, ^37^, ^38^, ^39^, ^40^ ^]^ However, there have been few reports on how DHPS is regulated in melanoma.

In this study, mRNA‐seq analysis showed that the number of genes up‐regulated by mRNA expression after DHPS knockdown was higher than the number of genes down‐regulated, suggesting that DHPS is closely related to the mRNA stability in melanoma cells. In contrast, MeRIP‐seq results showed that the number of genes down‐regulated by m6A after DHPS knockdown was lower than that of control in melanoma cells. This contradictory mechanism suggests that the oncogenic mechanism of DHPS is intricately linked to m6A modification.

**Figure 8:**
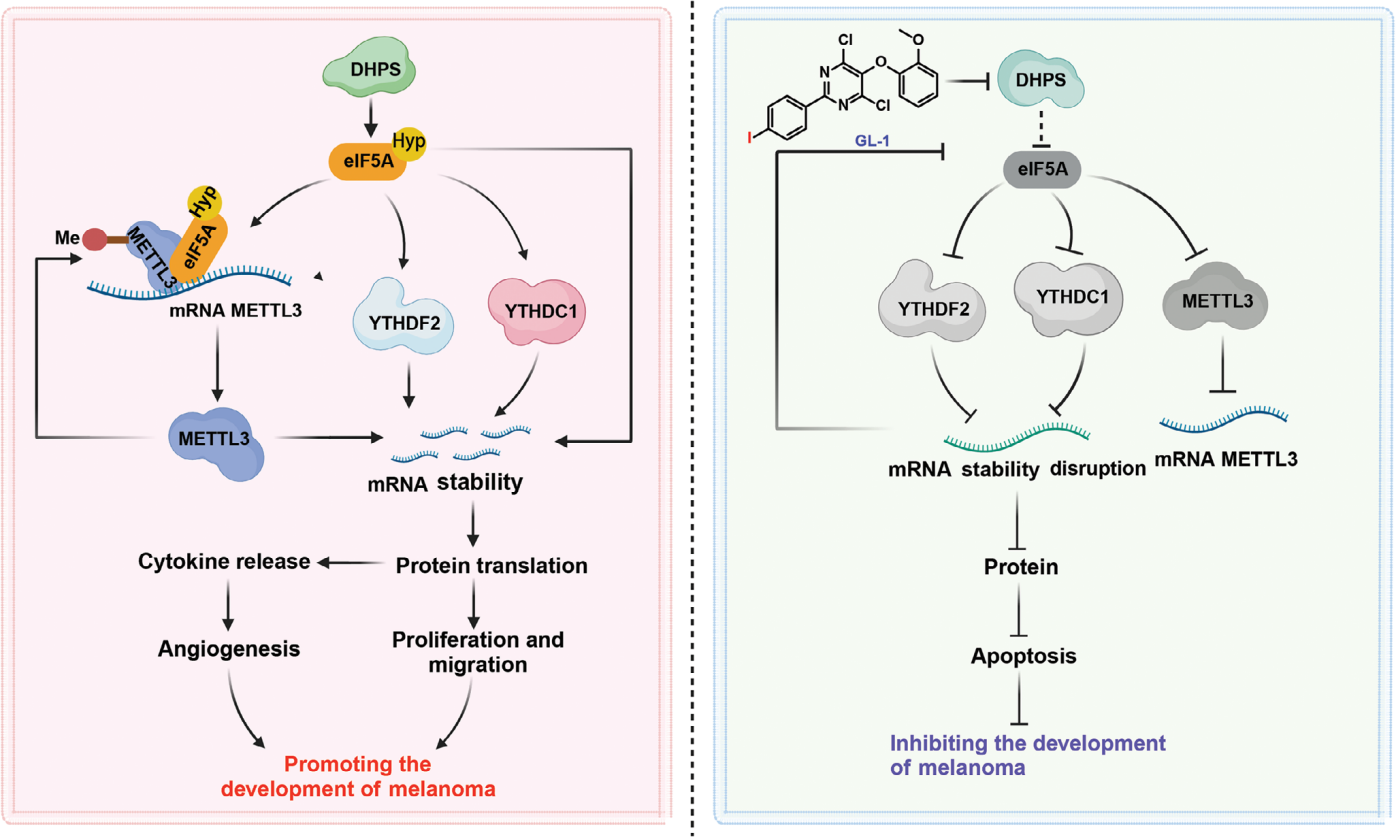
Regulatory mechanisms of DHPS in melanoma cells and the action of the targeted inhibitor GL‐1. Mechanistically, DHPS catalyzes the hypusination of eIF5A. This process has two effects: it mediates the self‐m6A‐methylation modification of METTL3, then promotes METTL3 protein expression and the mRNA translation process of YTHDF2 and YTHDC1. Consequently, DHPS plays a role in maintaining intracellular mRNA homeostasis and promoting cytokine expression, thereby sustaining the proliferative state of melanoma. When DHPS is inhibited, intracellular mRNA degradation is blocked, inhibiting various cytokine expressions and subsequently inhibiting melanoma cell proliferation.

The m6A modification is widely present in cancer cells, but the various levels of m6A in different cancers also predict the diversity of regulatory mechanisms.^[^ ^41^, ^42^, ^43^ ^]^ Hypomethylation in conjunctival melanoma has been shown to promote melanoma development due to reduced recognition of m6A by YTHDF2, which inhibits mRNA degradation and promotes the translation process.^[^ ^31^ ^]^ However, our studies on cutaneous melanoma have demonstrated that the knockdown of DHPS reduces both YTHDF2 and METTL3 proteins. This reduction directly affects the intracellular modification of m6A, which may be the main reason for the changes in mRNA stability observed in cutaneous melanoma cells. Subsequent studies revealed that METTL3 could not regulate the expression of YTHDC1/YTHDF2 proteins in melanoma, which also predicts that the methylation modifications regulated by METTL3 are not required for all genes, but that METTL3‐mediated m6A‐methylation modification is dependent on DHPS. Mechanistic studies revealed that the binding of METTL3 to eIF5A‐Hyp induced METTL3 to modify its self‐m6A‐methylation, and DHPS was a crucial factor in promoting eIF5A‐Hyp expression. Therefore, our results suggest that DHPS is a promising anti‐melanoma target that regulates intracellular m6A‐methylation modification and influences protein translation (**Figure** **8**).

The development of new drugs represents a significant objective of mechanistic studies. Traditional DHPS inhibitors are spermidine analogs, of which **GC‐7** is the most widely used.^[^ ^44^ ^]^ In recent years, both Takeda Pharmaceutical Laboratories and our team have been working on developing novel DHPS allosteric inhibitors.^[^ ^45^, ^46^ ^]^ In this study, a series of novel DHPS inhibitors were optimized and synthesized based on the lead structure, and the hit compound **GL‐1** was obtained by enzyme activity assay and cell viability assay. **GL‐1** inhibits the catalytic effect of DHPS on eIF5A by interacting with four amino acid residues in the active pocket of DHPS to form a stable binding. Of these four amino acid residues, K329 is the most important, as it is the essential site for the attachment of hypusine. Meanwhile, point mutation experiments showed that V129 and L286, like K329, could abolish the inhibitory effect of **GL‐1** on cell viability, and compared with these three sites, the mutation of the D238 site was slightly less effective. These results support that **GL‐1** is a DHPS inhibitor and provide a reference for the design of future DHPS inhibitors.

Surprisingly, both **GL‐1** and **GC‐7** could promote Cu^2+^ accumulation in melanoma cells, and this finding inspires the subsequent study of the regulatory relationship between DHPS and copper metabolism. Meanwhile, the results of the cellular multifactor assays showed that both **GL‐1** and **GC‐7** inhibited the expression of ANG, PAI‐1, TSP‐1, PDGF, VEGF, FGF2, IGFBP‐3, CSF2, CD71, CXCL1, and MMP9; This also suggests that the inhibition of DHPS activity could effectively inhibit the angiogenesis process of melanoma, which provides a new reference for the indication of DHPS inhibitors. The excellent bioavailability of **GL‐1**, low toxicity, and impressive TGI make **GL‐1** a promising candidate for inhibitor development.

Our study has some limitations. Mechanistically, the regulation of METTL3 by eIF5A‐Hyp was significantly different from that of YTHDF2, suggesting that eIF5A‐Hyp may have a unique translational regulation mechanism independent of epitope modification. Meanwhile, the effect of DHPS inhibitors on Cu^2+^ homeostasis in melanoma cells was found in the study of the anticancer mechanism of **GL‐1**, and DHPS may be involved in the metabolic regulation of melanoma cells. These unanticipated findings provide new directions for subsequent studies of the DHPS pro‐carcinogenic mechanism.

In conclusion, this study has innovatively identified DHPS‐mediated m6A methylation modification in melanoma and obtained a novel DHPS allosteric inhibitor, **GL‐1**, which had high efficiency, low toxicity, and good bioavailability in vivo and in vitro. These results provide a new reference for the pathogenesis of melanoma and the clinical treatment of melanoma.

## 4 Experimental Section

### Bioinformatics Analysis

Cancer Genome Atlas (TCGA) was used to analyze tumor/normal differential expression.^[^ ^32^ ^]^

### Tissue Microarray

The expression levels of DHPS and Hypusine in melanoma samples were determined using IHC analysis. Tissue microarrays consisting of 111 melanoma samples and 18 adjacent normal tissues were used. Further information on this can be found in the Section S3.1 (Supporting Information).

### Cell Lines and Transfection

A375, SK‐MEL‐28, B16‐F10, and HACAT. The following cell lines were used in this study: All cell lines underwent STR identification and were examined for mycoplasma contamination. The DHPS shRNA knockdown plasmid was constructed using the target sequence “CCACATACTTGGGCGAGTTTA.” The plasmid was then transformed into receptor cells, and single clones were selected and shaken overnight. The DHPS knockdown plasmid was extracted using the Plasmid Extraction Kit (Tiangen, China) and identified through enzymatic sequencing. Following the lentiviral packaging kit instructions, the target or control plasmid and virus were co‐transfected into 293T cells. After 48 h, the virus solution was concentrated, and quality testing and titer determination were performed. To transfect cells, they were spread into 24‐well plates and the appropriate amount of virus was added based on the previously measured titer value. Infection occurred within 24 h after A375 and SK‐MEL‐28 cell attachment, and fluorescence was observed 72 h later. Infection efficiency was determined using qPCR and Western blot. Details regarding the cell line supplier and culture conditions can be found in Section S5.2 (Supporting Information).

### mRNA‐Seq and MeRIP‐Seq

The mRNA was separated from A375 cells, and the samples were sequenced and analyzed by mRNA. Purified RNA was quantified, and the quality of the mRNA was assessed. The m6A Immunoprecipitation (MeRIP) procedure was used according to the instructions issued by the manufacturer. Detailed information can be found in the Section S3.3 (Supporting Information).

### RNA Immunoprecipitation (RIP) and RNA Stability Assays

The Magna RIP RNA‐binding protein immunoprecipitation kit (# 17–700, Millipore, MA) was used for RIP detection, following the manufacturer's instructions. The experimental procedures are detailed in Section S3.4 (Supporting Information). The primer sequence for METTL3 mRNA is as follows: forward primer 5′‐TGGAAGGGTGTTTTGGAGGA‐3′; reverse primer 5′‐GGTCAACTCCCTGTCCTGAA‐3′.

A375 and SK‐MEL‐28 cells were transfected with shDHPS and MT‐siMETTL3, respectively, while untreated controls were set up. The above cells were treated with a final concentration of 10 µg mL^−1^ of actinomycin D (ActD, CAS#:50‐76‐0, Bioss), and intracellular total RNA was extracted and subjected to qRT‐PCR at 0, 1, 2, 4, and 6 h time points according to the RNA extraction kit instructions (CAS#64‐17‐5, Takara). The half‐life of Ki67 and METTL3 were analyzed using the one‐phase decay model assay in GraphPad Prism 6 (GraphPad InC, USA).

### Colony Formation Assay

A375, SK‐MEL‐28, and B16‐F10 cells were inoculated in the six‐well plate at a density of 400 cells per well. 24 h later, compounds **GL‐1**(0, 0.5, 1.5, 2.0 µm) and **GC‐7** (2.0 µm) with different concentrations were added for 10 days. The cell colonies were fixed with 4% paraformaldehyde for 20 min and stained with crystal violet for 15 min. After washing with PBS 3 times, the number of cell colonies was detected.

### Western Blot and Co‐Immunoprecipitation (Co‐IP) Assays

Protein was extracted from A375, SK‐MEL‐28, or B16‐F10 cells after treatment with different concentrations of compounds **GL‐1** or **GC‐7** for 24 h. For Western blot, we used 10% or 15% polyacrylamide electrophoresis gel to separate and transfer to polyvinyl fluoride (PVDF) membrane for routine experiments. Co‐IP assays (#635721, Takara) were operated by the vendor's guidelines. The primary antibodies used are detailed in Table S18 (Supporting Information).

### General Methods

The chemicals and solvents needed for chemical synthesis were used directly. The methods for synthesis and data confirming the structure can be found in “Sections S1–S3” (Supporting Information). The instruments used for thin‐layer chromatography, HRMS data collection, and melting point measurement were used according to the literature with minor modifications.^[^ ^13^ ^]^

### Deoxyhypusine Synthase (DHPS) Assays

The NAD/NADH‐Glo assay (Promega, USA) was used in this study, as per the manufacturer's instructions. Detailed steps were conducted according to the literature with minor modifications.^[^ ^13^ ^]^ The concentrations of compounds were 1, 5, 10, 50, 100, 200, 400, 800, 1600, 2000 nm.

### Surface Plasmon Resonance (SPR)

The experiment was conducted at 25 °C using a CM5 sensor chip on the BIAcore T200, and data collection and analysis were completed using the BIAcore T200 evaluation software (GE Healthcare) according to the manufacturer's instructions. It was exported to Origin 7 software (v.7.0552, Origin Lab) to generate the final data. Detailed steps are provided in the Section S3.5 (Supporting Information).

### Cell Counting Kit‐8 (CCK‐8) Assays

Cell counting kit 8 (CCK‐8, GlpBio, USA) was used to evaluate the growth inhibition effects of target compounds and **GC‐7** on A375, SK‐mel‐28, HACAT, and B16‐F10 cells, and to establish a blank and control group, the concentrations of the compounds were 0.001, 0.01, 0.02, 0.035, 0.0625, 0.125, 0.25, 0.50, 1.00, 2.00, 4.00, 8.00 µm. The absorbance of the cells was measured at 450 nm by enzyme‐labeled apparatus. The number of living cells was proportional to absorbance.

### Cellular Thermal Shift Assay (CETSA) and Drug Affinity Responsive Target Stability (DARTS) Assay

For the CETSA assay, the cell lysate was divided into two equal parts. One part was used as a control, while the other was incubated with **GL‐1** (0.2 µm) at room temperature for 1 h. In the DARTS assay, 3 µL of **GL‐1** was added to a mixture of 297 µL of cell lysate supernatant and TNC solution. After incubation for 1 h, the mixture was divided into 6 centrifuge tubes with 50 µL each. Different proportions of pronase (#537088, Millipore, USA) solutions were added according to BCA results. The source of reagents and the experimental procedure for CETSA and DARTS were conducted according to the literature with minor modifications.^[^ ^47^ ^]^

### Docking

Molecular docking of DHPS with the inhibitor **GL‐1** was studied using MOE (Molecular Operating Environment, version 2016.08, Chemical Computing Group Inc., Canada). The docking site was set to “ligand” (**GL‐1**), and the parameters were left at their default state. After optimization and Protonate 3D, the docking structure was minimized for energy in the Amber10: EHT force field.

### Analysis of Apoptosis

The Annexin V‐FITC/PI Apoptosis detection kit was purchased from Vzyme BioTech Co. (#N401‐01, Vzyme). A375, SK‐MEL‐28, and B16‐F10 cells were stained with Annexin V‐FITC and PI for 15 min. The stained cells' spontaneous apoptosis was detected and analyzed using LSRFortessa flow cytometry (BD Biosciences, USA). The experiment was repeated three times.

### Cell Copper (Cu) Colorimetric Assay Kit

The Cell Copper (Cu) Colorimetric Assay Kit (Complexing Method) (#E‐BC‐K318‐M, Elabscience) was utilized to quantify the amount of Cu^2+^ present in the cells. A total of 2 × 106 cells were added to 200 uL of lysate. After sample processing, 100 uL of cell homogenate was taken for detection following the reagent's operation table.

### Human High‐Throughput Antibody Microarray Array

Rinse the cells with PBS and be sure to remove all PBS completely before adding the lysis buffer. A375 cells were dissolved with lysis buffer at a concentration of 1 × 107 cells mL^−1^. After resuspension, gently shake the lysate at 2–8 °C for 30 min. Microcentrifuge 14 000 × g for 5 min and transfer the supernatant to a clean test tube. It was recommended to use a total protein assay to determine the protein concentration of samples. Perform experiments following the instructions provided by the reagent manufacturer (Catalog #: ARY007, Catalog #: ARY0022B, R&D Systems Hong Kong Limited). For detailed procedures, refer to Section S3.6 (Supporting Information).

### Tumor Xenograft Model

BALB/c nude mice (4–5 weeks of age) were provided by HFK BIOSCIENCE CO., LTD (Beijing, China) in a pathogen‐free environment. 1 × 107 A375 cells were subcutaneously implanted into nude mice with 100 µL PBS. Detailed experimental procedures are in the Section S3.7 (Supporting Information). The calculation formula for tumor volume is as follows:length×width2/2, (Liaoning Province, China).Tumor growth inhibition (TGI) = [1‐ relative tumor volume (**GL**‐group)/relative tumor volume (control group)]*100%.

### Pharmacokinetic Studies

The pharmacokinetics of **GL‐1** were studied through both intravenous and intraperitoneal injection. The procedure and dosage details are provided in Section S3.8 (Supporting Information).

### Immunochemistry (IHC)

Melanoma tissue samples were fixed with formaldehyde, embedded in paraffin wax, sectioned, and immunostained with antibodies against target proteins. The primary antibodies were diluted at 1:2000, and their types were specified in Table S19 (Supporting Information). For IHC staining, streptavidin‐peroxidase complexes were used, and images were obtained with a light microscope.

### Statistical Analysis

Statistical analysis was conducted using GraphPad Prism 6 (GraphPad Inc, USA) following the method described in the literature with minor modifications.^[^ ^13^ ^]^

### Ethical Statement

The human tissue microarrays were purchased from Shanghai Outdo Biotech Company (Shanghai, China) and were reviewed by the Ethics Committee of the Shanghai Outdo Biotech Company Biobank (Ethical Number: SHYJS‐CP‐1910015). The animal experiment scheme was reviewed and approved by the Committee on the Use of Live Animals in Teaching and Research of Shengjing Hospital of China Medical University (Shenyang, Liaoning, China, Ethical Number: 2023PS1081K).

## Conflict of Interest

The authors declare no conflict of interest.

## Supporting information

Supporting Information

## Data Availability

The data that support the findings of this study are available in the supplementary material of this article.

## References

[1] C. Karimkhani , A. C. Green , T. Nijsten , M. A. Weinstock , R. P. Dellavalle , M. Naghavi , C. Fitzmaurice , Brit. J. Dermatol. 2015, 177, 134. 10.1111/bjd.15510 PMC5575560 28369739

[2] E. Linos , S. M. Swetter , M. G. Cockburn , G. A. Colditz , C. A. Clarke , J. Investigat. Dermatol. 2009, 129, 1666. 10.1038/jid.2008.423 PMC2866180 19131946

[3] Center for Disease Control and Prevention (CDC) , G. P. Guy Jr , C. C. Thomas , T. Thompson , M. Watson , G. M. Massetti , L. C. Richardson , MMWR. Morb. Mortal. Wkly Rep. 2015, 64, 591. 26042651 PMC4584771

[4] D. C. Whiteman , A. C. Green , C. M. Olsen , J. Invest. Dermatol. 2016, 136, 1161. 26902923 10.1016/j.jid.2016.01.035

[5] R. L. Siegel , K. D. Miller , N. S. Wagle , A. Jemal , CA: Cancer J. Clin. 2023, 73, 17. 36633525 10.3322/caac.21763

[6] D. B. Johnson , A. B. Daniels , JAMA Ophthalmol. 2018, 136, 986. 29955760 10.1001/jamaophthalmol.2018.1813

[7] M. F. Avril , S. Aamdal , J. J. Grob , A. Hauschild , P. Mohr , J. J. Bonerandi , M. Weichenthal , K. Neuber , T. Bieber , K. Gilde , V. Guillem Porta , J. Fra , J. Bonneterre , P. Saïag , D. Kamanabrou , H. Pehamberger , J. Sufliarsky , J. L. Gonzalez Larriba , A. Scherrer , Y. Menu , J. Clin. Oncol. 2004, 22, 1118. 15020614 10.1200/JCO.2004.04.165

[8] ESMO Guidelines Committee , O. Michielin , A. C. J. van Akkooi , P. A. Ascierto , R. Dummer , U. Keilholz , Ann. Oncol. 2019, 30, 1884. 31566661 10.1093/annonc/mdz411

[9] M. L. Hawkins , M. J. Rioth , M. M. Eguchi , M. Cockburn , J. Am. Acad. Dermatol. 2019, 80, 1640. 30654077 10.1016/j.jaad.2019.01.009 PMC7232854

[10] E. C. Wolff , K. R. Kang , Y. S. Kim , M. H. Park , Amino Acids 2007, 33, 341. 17476569 10.1007/s00726-007-0525-0 PMC2572820

[11] M. H. Park , E. C. Wolff , J. Biol. Chem. 2018, 293, 18710. 30257869 10.1074/jbc.TM118.003341 PMC6290153

[12] M. H. Park , K. Nishimura , C. F. Zanelli , S. R. Valentini , Amino Acids 2010, 38, 491. 19997760 10.1007/s00726-009-0408-7 PMC2829442

[13] K. L. Liu , X. Y. Li , D. P. Wang , W. H. Xue , X. H. Qian , Y. H. Li , Q. Q. Lin , S. Li , F. H. Meng , J. Med. Chem. 2021, 64, 13356. 34473510 10.1021/acs.jmedchem.1c00582

[14] S. Coni , R. Bordone , D. M. Ivy , Z. N. Yurtsever , L. Di Magno , R. D'Amico , B. Cesaro , A. Fatica , F. Belardinilli , F. Bufalieri , M. Maroder , E. De Smaele , L. Di Marcotullio , G. Giannini , E. Agostinelli , G. Canettieri , Cancer Lett. 2023, 559, 216120. 36893894 10.1016/j.canlet.2023.216120

[15] Z. Zhang , G. He , Y. Lv , Y. Liu , Z. Niu , Q. Feng , R. Hu , J. Xu , Cell Death Dis. 2022, 13, 74. 35064108 10.1038/s41419-022-04511-7 PMC8782983

[16] H. Zhang , G. Alsaleh , J. Feltham , Y. Sun , G. Napolitano , T. Riffelmacher , P. Charles , L. Frau , P. Hublitz , Z. Yu , S. Mohammed , A. Ballabio , S. Balabanov , J. Mellor , A. K. Simon , Mol. Cell. 2019, 76, 110. 31474573 10.1016/j.molcel.2019.08.005 PMC6863385

[17] V. Mudryi , F. Peske , M. Rodnina , BBA Adv. 2023, 3, 100074. 37082265 10.1016/j.bbadva.2023.100074 PMC10074943

[18] X. Wang , R. Wu , Y. Liu , Y. Zhao , Z. Bi , Y. Yao , Q. Liu , H. Shi , F. Wang , Y. Wang , Autophagy 2020, 16, 1221. 31451060 10.1080/15548627.2019.1659617 PMC7469583

[19] C. M. Wei , A. Gershowitz , B. Moss , Cell 1975, 4, 379. 164293 10.1016/0092-8674(75)90158-0

[20] J. A. Bokar , M. E. Shambaugh , D. Polayes , A. G. Matera , F. M. Rottman , RNA 1997, 3, 1233. 9409616 PMC1369564

[21] J. Yu , P. Chai , M. Xie , S. Ge , J. Ruan , X. Fan , R. Jia , Genome Biol. 2021, 22, 85. 33726814 10.1186/s13059-021-02308-z PMC7962360

[22] H. Z. Shi , J. S. Xiong , L. Gan , Y. Zhang , C. C. Zhang , Y. Q. Kong , Q. J. Miao , C. C. Tian , R. Li , J. Q. Liu , E. J. Zhang , W. B. Bu , Y. Wang , X. F. Cheng , J. F. Sun , H. Chen , Clin. Transl. Med. 2022, 12, e1075. 36324258 10.1002/ctm2.1075 PMC9630608

[23] G. Wang , D. Zeng , E. Sweren , Y. Miao , R. Chen , J. Chen , J. Wang , W. Liao , Z. Hu , S. Kang , L. A. Garza , J. Invest. Dermatol. 2023, 143, 1579. 36842525 10.1016/j.jid.2023.01.027 PMC10363194

[24] J. Meng , X. Huang , Y. Qiu , M. Yu , J. Lu , J. Yao , Int. J. Gen. Med. 2021, 14, 5345. 34522131 10.2147/IJGM.S328522 PMC8434882

[25] J. Tang , Q. Wan , J. Lu , BMC Cancer 2020, 20, 674 32682400 10.1186/s12885-020-07159-8 PMC7368742

[26] U. Dahal , K. Le , M. Gupta , Melan. Res. 2019, 29, 382. 10.1097/CMR.0000000000000580 30762711

[27] S. Chu , Y. Li , B. Wu , G. Rong , Q. Hou , Q. Zhou , D. Du , Y. Li , Cell Transplant. 2023, 32, 9636897231188300. 37606168 10.1177/09636897231188300 PMC10467386

[28] H. Wu , H. Xu , D. Jia , T. Li , L. Xia , Ann. Transl. Med. 2021, 9, 1155. 34430596 10.21037/atm-21-2906 PMC8350655

[29] S. Yang , J. Wei , Y. H. Cui , G. Park , P. Shah , Y. Deng , A. E. Aplin , Z. Lu , S. Hwang , C. He , Y. Y. He , Nat. Commun. 2019, 10, 2782. 31239444 10.1038/s41467-019-10669-0 PMC6592937

[30] N. Li , Y. Kang , L. Wang , S. Huff , R. Tang , H. Hui , K. Agrawal , G. M. Gonzalez , Y. Wang , S. P. Patel , T. M. Rana , Proc. Natl. Acad. Sci. USA 2020, 117, 20159. 32747553 10.1073/pnas.1918986117 PMC7443867

[31] R. Jia , P. Chai , S. Wang , B. Sun , Y. Xu , Y. Yang , S. Ge , R. Jia , Y. G. Yang , X. Fan , Mol. Cancer 2019, 18, 161. 31722709 10.1186/s12943-019-1088-x PMC6854757

[32] M. S. Cline , B. Craft , T. Swatloski , M. Goldman , S. Ma , D. Haussler , J. Zhu , Sci. Rep. 2013, 3, 2652. 24084870 10.1038/srep02652 PMC3788369

[33] K. D. Meyer , Y. Saletore , P. Zumbo , O. Elemento , C. E. Mason , S. R. Jaffrey , Cell 2012, 149, 1635. 22608085 10.1016/j.cell.2012.05.003 PMC3383396

[34] D. J. Puleston , F. Baixauli , D. E. Sanin , J. Edwards‐Hicks , M. Villa , A. M. Kabat , M. M. Kamiński , M. Stanckzak , H. J. Weiss , K. M. Grzes , K. Piletic , C. S. Field , M. Corrado , F. Haessler , C. Wang , Y. Musa , L. Schimmelpfennig , L. Flachsmann , G. Mittler , N. Yosef , E. L. Pearce , Cell 2021, 184, 4186. 34216540 10.1016/j.cell.2021.06.007 PMC8358979

[35] D. J. Puleston , M. D. Buck , R. I. Klein Geltink , R. L. Kyle , G. Caputa , D. O'Sullivan , A. M. Cameron , A. Castoldi , Y. Musa , A. M. Kabat , Y. Zhang , L. J. Flachsmann , C. S. Field , A. E. Patterson , S. Scherer , F. Alfei , F. Baixauli , S. K. Austin , B. Kelly , M. Matsushita , E. L. Pearce , Cell Metab. 2019, 30, 352. 31130465 10.1016/j.cmet.2019.05.003 PMC6688828

[36] P. Saini , D. E. Eyler , R. Green , T. E. Dever , Nature 2009, 459, 118. 19424157 10.1038/nature08034 PMC3140696

[37] A. P. Schuller , C. C. Wu , T. E. Dever , A. R. Buskirk , R. Green , Mol. Cell 2017, 66, 194. 28392174 10.1016/j.molcel.2017.03.003 PMC5414311

[38] Z. A. Jenkins , P. G. Hååg , H. E. Johansson , Genomics 2001, 71, 101. 11161802 10.1006/geno.2000.6418

[39] E. Gutierrez , B. S. Shin , C. J. Woolstenhulme , J. R. Kim , P. Saini , A. R. Buskirk , T. E. Dever , Mol. Cell 2013, 51, 35. 23727016 10.1016/j.molcel.2013.04.021 PMC3744875

[40] V. Pelechano , P. Alepuz , Nucleic Acids Res. 2017, 45, 7326. 28549188 10.1093/nar/gkx479 PMC5499558

[41] A. Uzonyi , D. Dierks , R. Nir , O. S. Kwon , U. Toth , I. Barbosa , C. Burel , A. Brandis , W. Rossmanith , H. Le Hir , B. Slobodin , S. Schwartz , Mol. Cell 2023, 83, 237. 36599352 10.1016/j.molcel.2022.12.026

[42] X. Wang , Z. Lu , A. Gomez , G. C. Hon , Y. Yue , D. Han , Y. Fu , M. Parisien , Q. Dai , G. Jia , B. Ren , T. Pan , C. He , Nature 2014, 505, 117. 24284625 10.1038/nature12730 PMC3877715

[43] L. Allegri , F. Baldan , E. Molteni , C. Mio , G. Damante , Int. J. Mol. Sci. 2022, 23, 11516. 36232810 10.3390/ijms231911516 PMC9569446

[44] B. P. Jansson , L. Malandrin , H. E. Johansson , J. Bacteriol. 2000, 182, 1158. 10648545 10.1128/jb.182.4.1158-1161.2000 PMC94395

[45] Y. Tanaka , O. Kurasawa , A. Yokota , M. G. Klein , K. Ono , B. Saito , S. Matsumoto , M. Okaniwa , G. Ambrus‐Aikelin , D. Morishita , S. Kitazawa , N. Uchiyama , K. Ogawa , H. Kimura , S. Imamura , J. Med. Chem. 2020, 63, 3215. 32142284 10.1021/acs.jmedchem.9b01979

[46] Y. Tanaka , O. Kurasawa , A. Yokota , M. G. Klein , B. Saito , S. Matsumoto , M. Okaniwa , G. Ambrus‐Aikelin , N. Uchiyama , D. Morishita , H. Kimura , S. Imamura , ACS Med. Chem. Lett. 2020, 11, 1645. 34345355 10.1021/acsmedchemlett.0c00331 PMC8323115

[47] G. Dong , Y. H. Li , J. S. Guo , Q. Q. Lin , M. Y. Deng , W. H. Xue , X. Y. Li , F. H. Meng , Eur. J. Med. Chem. 2023, 258, 115600. 37437348 10.1016/j.ejmech.2023.115600

