# Supplementary material for: DHPS‐Mediated Hypusination Regulates METTL3 Self‐m6A‐Methylation Modification to Promote Melanoma Proliferation and the Development of Novel Inhibitors
Source: Adv Sci (Weinh). 2024 Jul 1;11(33):2402450. doi: 10.1002/advs.202402450 (PMC11434010; doi:10.1002/advs.202402450)
Supplement: Supplementary file 1 — Supporting Information [file ADVS-11-2402450-s001.docx]

DHPS-mediated hypusination regulates METTL3 self-m6A-methylation modification to promote melanoma proliferation and the development of novel inhibitors

Jing-si Guo, Jian Ma, Xi-he Zhao, Ji-fang Zhang, Kai-li Liu, Long-tian Li, Yu-xi Qin, Fan-hao Meng, Ling-yan Jian, Yue-hui Yang, Xin-yang Li ^*^

A. Jing-si Guo, Ji-fang Zhang, Long-tian Li, Yu-xi Qin, Ling-yan Jian, Yue-hui Yang, Xin-yang Li

Department of Pharmacy, Shengjing Hospital of China Medical University, Shenyang, 1110004, P. R. China.
E-mail: xinyanglicmu@163.com/20202420@cmu.edu.cn

B. Jian Ma
Department of Obstetrics and Gynecology, Shengjing Hospital of China Medical University, Shenyang 110004, China

C. Xi-he Zhao
Department of Oncology, Shengjing Hospital of China Medical University, Shenyang 110004, China

D. Kai-li Liu

School of Pharmaceutical Engineering, Jining Medical College, University Park, No.16 Haichuan Road, Gaoxin District, Jining City, Shandong Province, China

E. Fan-hao Meng

School of Pharmacy, China Medical University, Shenyang 110122, P. R. China.


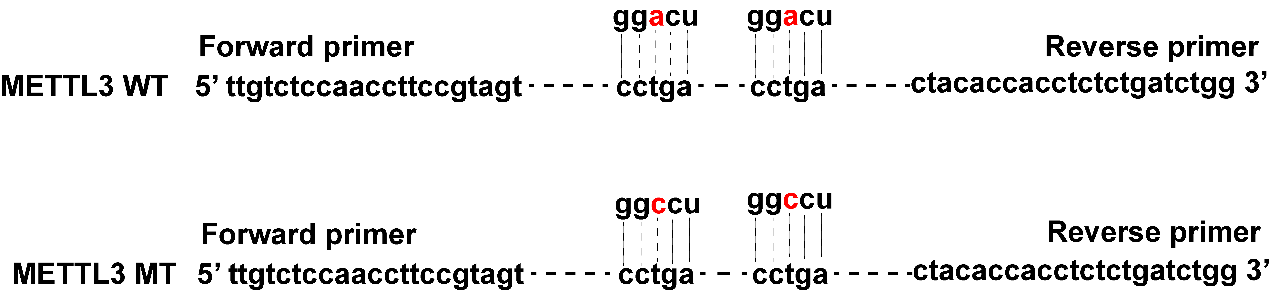


***Scheme 1.*** ***METTL3 mRNA adenylate (A) site mutated to cytosine (C).***

***1.*** ***Design and synthesis of target compounds***

*1.1 Schematic design of target compounds*

***­***

***Scheme 2. Design of target compounds.***

*1.2 Chemistry*

The synthetic pathway adopted to prepare target compounds was depicted in **Scheme 3**. By brominating the starting material Diethyl malonate (compound **1**), compound **2** was obtained which immediately reacted with guaiacol to obtain key intermediate **3** in high yield. Meanwhile, **5a-5j**were prepared from **4a-4j** via Pinner reaction at low temperatures and reacted with intermediate **3** through cyclization reaction to get target compounds **6a-6j**. Other target compounds **7a-7f** were prepared from the mixture of **6a-6f** and phosphorus oxychloride.

**Scheme 3.** Reagents and conditions: (i) NBS, H_2_SO_4_, CHCl_3_, 50^°^C, 8 h; (ii) guaiacol, K_2_CO_3_, acetonitrile, 60^°^C, overnight; (iii a) Na, CH_3_OH, CH_2_Cl_2_, rt, 48 h; NH_4_Cl, rt, overnight; or (iii b) ethanol, acetyl chloride, CH_2_Cl_2_, rt, 24 h; Na, NH_4_Cl, CH_3_OH, rt, overnight; (iv) Na, CH_3_OH, rt, 24 h; (v) POCl_3_, 100^°^C, overnight.

Unless otherwise indicated, reagents and solvents were purchased from commercial sources and used without further purification. NMR spectra were measured on a 600 MHz Bruker unit (500 MHz for ^1^H NMR, 126 MHz for ^13^C NMR) using DMSO-*d_6_* as the solvent at room temperature. APCI-HRMS data were gathered using a Bruker micro TOF-Q instrument. Analytical TLC was performed on silica gel 60 F254 plates (Qingdao Haiyang Chemcal Company, Ltd) and visualized by UV. Melting points were determined with an Electro thermal melting point apparatus, were uncorrected.

*1.3 The synthesis of diethyl 2-bromomalonate (****2****)*

To a solution of diethyl malonate (40.00 g, 249.74 mmol) in chloroform (150 mL), *N*-Bromosuccinimide (46.67 g, 262.22 mmol) and 2 mL of concentrated sulfuric acid were added successively. Then the mixture was stirred for 8 h at 50^°^C. After the completion of the reaction, the mixture was successively washed by water and brine, followed by drying with anhydrous sodium sulfate. Finally, the mixture was evaporated in vacuum to obtain diethyl 2-bromomalonate as a colorless transparent liquid, yield 98.2%. The product was used directly in the next step.

Small amount of the product was purified by silica gel column and verified by NMR spectra. ^1^H NMR (600 MHz, DMSO-*d_6_*) *δ* 5.56 (s, 1H), 4.22 (q, *J* = 7.1 Hz, 4H), 1.21 (t, *J* = 7.1 Hz, 6H). ^13^C NMR (151 MHz, DMSO-*d_6_*) *δ* 165.13 (2C), 63.17 (2C), 43.67, 14.17 (2C).

*1.4 The synthesis of diethyl 2-(2-methoxyphenoxy)malonate (****3****)*

To a solution of 2-bromomalonate (58.60 g, 245.12 mmol) in acetonitrile (150 mL), guaiacol (30.43 g, 245.12 mmol) and K_2_CO_3_ (47.43 g, 343.17 mmol) were added successively. Then the mixture was stirred at 60^°^C overnight and monitored by TLC. After the completion of the reaction, the formed solid was removed by filter, the filtrate evaporated in vacuum and dissolved with dichloromethane, then the solution was successively washed by water and brine, followed by drying with anhydrous sodium sulfate. Finally, the mixture was evaporated in vacuum to obtain 2-(2-methoxyphenoxy)malonate as a pale yellow liquid. The product was used directly in the next step.

Small amount of the product was purified by silica gel column and verified by NMR spectra. ^13^C NMR (151 MHz, DMSO-*d_6_*) *δ* 165.99 (2C), 150.14, 146.23, 124.02, 121.09, 117.28, 113.56, 77.63, 62.19 (2C), 56.16, 14.31 (2C).

*1.5 General procedure for preparation of compounds* ***5a-5j***

*1.5.1 General procedure for preparation of compounds* ***5a-5f***

Small pieces of metal sodium (1.34 g, 58.15 mmol) were slowly added in methanol (100 mL) at 0^°^C, after no more gas was generated, 3-chlorobenzonitrile (**4a**) (10.00 g, 72.69 mmol) and dichloromethane (50 mL) were added into the mixture. Then the mixture was stirred for 48 h at room temperature and monitored by TLC. Next, ammonium chloride (6.22 g, 116.31 mmol) was added into the mixture which was stirred at room temperature overnight and monitored by TLC. After the completion of the reaction, the insoluble solid was removed by filter, the filtrate evaporated in vacuum to obtain 3-chlorobenzimidamide (**5a**) as white solid. Although the product contained little unreacted raw materials, it could be used directly in the next step.

Compounds **5b-5f** were synthesized in the same method from compounds **4b-4f**.

*1.5.2 General procedure for preparation of compounds* ***5g-5j***

Acetyl chloride (7.77 g, 98.95 mmol) was slowly and dropwise added in the solution of ethanol (7.60 g, 164.92 mmol) in dichloromethane (100 mL) at 0^°^C. The mixture was stirred for 0.5 h after the addition was complete. Then 2-(3-chlorophenyl)acetonitrile (**4g**) (10.00 g, 65.97 mmol) was added in the mixture that was stirred for 24 h at 0^°^C and monitored by TLC. Next, methanol solution of ammonia, obtained from the mixture of metal sodium (4.55 g, 197.90 mmol), ammonium chloride (10.59 g, 197.90 mmol) and 100 mL methanol, was slowly and dropwise added into the above mixture that was subsequently stirred at room temperature overnight and monitored by TLC. After the completion of the reaction, the insoluble solid was removed by filter, the filtrate evaporated in vacuum to obtain 3-chlorobenzimidamide (**5g**) as white solid. Although the product contained little unreacted raw materials, it could be used directly in the next step.

Compounds **5g-5j** were synthesized in the same method from compounds **4g-4j**.

*1.6 General procedure for preparation of compounds* ***6a-6j***

The small pieces of Na (3.34 g, 145.41 mmol) were slowly added into methanol (150 mL) at 0^°^C, after no more gas was generated, 3-chlorobenzimidamide (**5a**) (11.24 g, 72.71 mmol) and 2-(2-methoxyphenoxy)malonate (**3**) (20.52 g, 72.71 mmol) was added into the mixture that was subsequently stirred for 24 h at room temperature. After the completion of the reaction, the mixture was poured into water and adjusted pH to 10 with sodium hydroxide, the insoluble solid was removed by filter, the filtrate was adjusted pH to 2 with hydrochloric acid, the formed precipitate was collected by filtration, washed with water, dried and purified by beating with methanol to obtain 2-(3-chlorophenyl)-5-(2-methoxyphenoxy)pyrimidine-4,6-diol (**6a**) as a white solid.

Compounds **6b-6j** was synthesized in the same method from compounds **5b-5j** with compound **3**.

*1.6.1 2-(3-chlorophenyl)-5-(2-methoxyphenoxy)pyrimidine-4,6-diol (****6a****)*

White powder, yield: 52.9%, mp: 183.5-185.1^°^C. HPLC: 99.34%. ^1^H NMR (600 MHz, DMSO-*d_6_*) *δ* 12.17 (s, 2H), 8.18 (t, *J* = 1.7 Hz, 1H), 8.07 (d, *J* = 7.9 Hz, 1H), 7.65 (dd, *J* = 8.1, 1.6 Hz, 1H), 7.58 (t, *J* = 7.9 Hz, 1H), 7.03 (dd, *J* = 8.1, 1.1 Hz, 1H), 6.93 (td, *J* = 7.9, 1.3 Hz, 1H), 6.79 (t, *J* = 7.7 Hz, 1H), 6.67 (dd, *J* = 8.0, 1.2 Hz, 1H), 3.83 (s, 3H). ^13^C NMR (151 MHz, DMSO-*d_6_*) *δ* 160.94 (2C), 152.07, 148.80, 146.99, 134.56, 133.92, 131.67, 131.04, 128.07, 126.78, 122.21, 120.87, 119.61, 113.56, 113.20, 56.08. ESI-HRMS: calcd. for C_17_H_13_ClN_2_O_4_ [M+H]^+^ 345.0642, found: 345.0623.

*1.6.2 2-(4-chlorophenyl)-5-(2-methoxyphenoxy)pyrimidine-4,6-diol (****6b****)*

White powder, yield: 52.2%, mp: 171.3-172.3^°^C. HPLC: 98.16%. ^1^H NMR (600 MHz, DMSO-*d_6_*) *δ* 12.23 (s, 2H), 8.11 (d, *J* = 8.6 Hz, 2H), 7.62 (d, *J* = 8.6 Hz, 2H), 7.03 (d, *J* = 8.1 Hz, 1H), 6.92 (t, *J* = 7.7 Hz, 1H), 6.78 (t, *J* = 7.7 Hz, 1H), 6.67 (dd, *J* = 8.0, 1.2 Hz, 1H), 3.83 (s, 3H). ^13^C NMR (151 MHz, DMSO-*d_6_*) *δ* 160.94 (2C), 152.40, 148.80, 147.03, 136.88, 131.32, 130.06 (2C), 129.21 (2C), 122.15, 120.87, 119.40, 113.53, 113.18, 56.07. ESI-HRMS: calcd. for C_17_H_13_ClN_2_O_4_ [M+H]^+^ 345.0642, found: 345.0623.

*1.6.3 2-(3-bromophenyl)-5-(2-methoxyphenoxy)pyrimidine-4,6-diol (****6c****)*

White powder, yield: 63.4%, mp: 162.0-164.4^°^C. HPLC: 99.99%. ^1^H NMR (600 MHz, DMSO-*d_6_*) *δ* 12.18 (s, 2H), 8.32 (t, *J* = 1.7 Hz, 1H), 8.11 (d, *J* = 7.9 Hz, 1H), 7.78 (dd, *J* = 8.1, 1.5 Hz, 1H), 7.51 (t, *J* = 7.9 Hz, 1H), 7.03 (dd, *J* = 8.1, 1.3 Hz, 1H), 6.93 (td, *J* = 7.8, 1.5 Hz, 1H), 6.79 (td, *J* = 7.9, 1.4 Hz, 1H), 6.67 (dd, *J* = 8.0, 1.4 Hz, 1H), 3.83 (s, 3H). ^13^C NMR (151 MHz, DMSO-*d_6_*) *δ* 160.94 (2C), 151.98, 148.80, 147.00, 134.76, 134.53, 131.26, 130.97, 127.11, 122.34, 122.20, 120.87, 119.59, 113.56, 113.20, 56.09. ESI-HRMS: calcd. for C_17_H_13_BrN_2_O_4_ [M+H]^+^ 389.0137, found: 389.0121, 391.0081.

*1.6.4 2-(4-bromophenyl)-5-(2-methoxyphenoxy)pyrimidine-4,6-diol (****6d****)*

White powder, yield: 61.7%, mp: 199.5-200.6^°^C. HPLC: 95.23%. ^1^H NMR (600 MHz, DMSO-*d_6_*) *δ* 12.27 (s, 2H), 8.04 (d, *J* = 8.6 Hz, 2H), 7.76 (d, *J* = 8.6 Hz, 2H), 7.03 (dd, *J* = 8.1, 1.3 Hz, 1H), 6.93 (td, *J* = 7.9, 1.4 Hz, 1H), 6.78 (td, *J* = 7.9, 1.4 Hz, 1H), 6.67 (dd, *J* = 8.0, 1.4 Hz, 1H), 3.83 (s, 3H). ^13^C NMR (151 MHz, DMSO-*d_6_*) *δ* 160.95 (2C), 152.54, 148.79, 147.01, 132.14 (2C), 131.70, 130.24 (2C), 125.84, 122.16, 120.87, 119.41, 113.53, 113.17, 56.07. ESI-HRMS: calcd. for C_17_H_13_BrN_2_O_4_ [M+H]^+^ 389.0137, found: 389.0120, 391.0082.

*1.6.5 2-(3-iodophenyl)-5-(2-methoxyphenoxy)pyrimidine-4,6-diol (****6e****)*

White powder, yield: 58.0%, mp: 168.4-169.3^°^C. HPLC: 99.78%. ^1^H NMR (600 MHz, DMSO-*d_6_*) *δ* 12.11 (s, 2H), 8.49 (s, 1H), 8.11 (d, *J* = 7.9 Hz, 1H), 7.94 (d, *J* = 7.9 Hz, 1H), 7.34 (t, *J* = 7.9 Hz, 1H), 7.03 (dd, *J* = 8.1, 1.1 Hz, 1H), 6.93 (td, *J* = 7.9, 1.3 Hz, 1H), 6.79 (td, *J* = 7.7, 1.3 Hz, 1H), 6.66 (dd, *J* = 8.0, 1.3 Hz, 1H), 3.83 (s, 3H). ^13^C NMR (151 MHz, DMSO-*d_6_*) *δ* 160.90 (2C), 151.95, 148.80, 147.01, 140.32, 136.77, 134.50, 131.15, 127.42, 122.19, 120.87, 119.52, 113.55, 113.20, 95.28, 56.09. ESI-HRMS: calcd. for C_17_H_13_IN_2_O_4_ [M+H]^+^ 436.9998, found: 436.9970.

*1.6.6 2-(4-iodophenyl)-5-(2-methoxyphenoxy)pyrimidine-4,6-diol (****6f****)*

White powder, yield: 61.8%, mp: 175.4-178.2^°^C. HPLC: 95.98%. ^1^H NMR (600 MHz, DMSO-*d_6_*) *δ* 12.27 (s, 2H), 7.93 (d, *J* = 8.5 Hz, 2H), 7.87 (d, *J* = 8.5 Hz, 2H), 7.03 (dd, *J* = 8.2, 1.1 Hz, 1H), 6.92 (td, *J* = 7.8, 1.3 Hz, 1H), 6.78 (td, *J* = 7.8, 1.2 Hz, 1H), 6.66 (dd, *J* = 8.0, 1.3 Hz, 1H), 3.83 (s, 3H). ^13^C NMR (151 MHz, DMSO-*d_6_*) *δ* 160.94 (2C), 152.77, 148.79, 147.01, 137.99 (2C), 131.96, 130.06 (2C), 122.15, 120.88, 119.41, 113.53, 113.17, 99.84, 56.08. ESI-HRMS: calcd. for C_17_H_13_IN_2_O_4_ [M+H]^+^ 436.9998, found: 436.9972.

*1.6.7 2-(3-chlorobenzyl)-5-(2-methoxyphenoxy)pyrimidine-4,6-diol (****6g****)*

White powder, yield: 61.5%, mp: 186.4-188.4^°^C. HPLC: 98.96%. ^1^H NMR (600 MHz, DMSO-*d_6_*) *δ* 12.09 (s, 2H), 7.46 (s, 1H), 7.39 (t, *J* = 7.7 Hz, 1H), 7.35 (d, *J* = 8.2 Hz, 1H), 7.32 (d, *J* = 7.5 Hz, 1H), 6.99 (dd, *J* = 8.2, 1.1 Hz, 1H), 6.89 (td, *J* = 7.8, 1.3 Hz, 1H), 6.76 (td, *J* = 7.7, 1.2 Hz, 1H), 6.56 (dd, *J* = 8.0, 1.3 Hz, 1H), 3.88 (s, 2H), 3.79 (s, 3H). ^13^C NMR (151 MHz, DMSO-*d_6_*) *δ* 160.55 (2C), 156.57, 148.76, 147.11, 138.74, 133.54, 130.94, 129.47, 128.25, 127.50, 121.96, 120.86, 118.63, 113.36, 113.18, 56.07, 39.64. ESI-HRMS: calcd. for C_18_H_15_ClN_2_O_4_ [M+H]^+^ 359.0799, found: 359.0779.

*1.6.8 2-(4-chlorobenzyl)-5-(2-methoxyphenoxy)pyrimidine-4,6-diol (****6h****)*

White powder, yield: 64.1%, mp: 199.7-201.4^°^C. HPLC: 97.68%. ^1^H NMR (600 MHz, DMSO-*d_6_*) *δ* 12.33 (s, 2H), 7.43 (d, *J* = 8.5 Hz, 2H), 7.38 (d, *J* = 8.5 Hz, 2H), 6.99 (dd, *J* = 8.1, 1.3 Hz, 1H), 6.89 (td, *J* = 7.8, 1.4 Hz, 1H), 6.76 (td, *J* = 7.8, 1.4 Hz, 1H), 6.55 (dd, *J* = 8.0, 1.4 Hz, 1H), 3.86 (s, 2H), 3.80 (s, 3H). ^13^C NMR (151 MHz, DMSO-*d_6_*) *δ* 160.53 (2C), 156.78, 148.75, 147.11, 135.37, 132.22, 131.36 (2C), 129.03 (2C), 121.95, 120.85, 118.55, 113.31, 113.17, 56.07, 39.38. ESI-HRMS: calcd. for C_18_H_15_ClN_2_O_4_ [M+H]^+^ 359.0799, found: 359.0781.

*1.6.9 2-(3-bromobenzyl)-5-(2-methoxyphenoxy)pyrimidine-4,6-diol (****6i****)*

White powder, yield: 68.6%, mp: 164.7-165.9^°^C. HPLC: 98.70%. ^1^H NMR (600 MHz, DMSO-*d_6_*) *δ* 12.15 (s, 2H), 7.60 (s, 1H), 7.49 (d, *J* = 7.7 Hz, 1H), 7.40 – 7.29 (m, 2H), 6.99 (dd, *J* = 8.1, 1.2 Hz, 1H), 6.89 (td, *J* = 7.8, 1.4 Hz, 1H), 6.76 (td, *J* = 7.8, 1.4 Hz, 1H), 6.55 (dd, *J* = 8.0, 1.4 Hz, 1H), 3.87 (s, 2H), 3.79 (s, 3H). ^13^C NMR (151 MHz, DMSO-*d*_6_) δ 160.57 (2C), 156.59, 148.75, 147.10, 139.01, 132.34, 131.24, 130.39, 128.62, 122.19, 121.96, 120.86, 118.62, 113.36, 113.18, 56.08, 40.52. ESI-HRMS: calcd. for C_18_H_15_BrN_2_O_4_ [M+H]^+^ 403.0293, found: 403.0278, 405.0236.

*1.6.10 2-(4-bromobenzyl)-5-(2-methoxyphenoxy)pyrimidine-4,6-diol (****6j****)*

White powder, yield: 65.0%, mp: 181.2-183.8^°^C. HPLC: 99.64%. ^1^H NMR (600 MHz, DMSO-*d_6_*) *δ* 12.18 (s, 2H), 7.56 (d, *J* = 8.4 Hz, 2H), 7.32 (d, *J* = 8.4 Hz, 2H), 6.99 (dd, *J* = 8.1, 1.3 Hz, 1H), 6.89 (td, *J* = 7.8, 1.4 Hz, 1H), 6.75 (td, *J* = 7.9, 1.4 Hz, 1H), 6.55 (dd, *J* = 8.0, 1.4 Hz, 1H), 3.84 (s, 2H), 3.79 (s, 3H). ^13^C NMR (101 MHz, DMSO-*d_6_*) *δ* 160.51 (2C), 156.71, 148.75, 147.11, 135.79, 131.96 (2C), 131.74 (2C), 121.95, 120.86, 120.73, 118.56, 113.32, 113.18, 56.07, 39.45. ESI-HRMS: calcd. for C_18_H_15_BrN_2_O_4_ [M+H]^+^ 403.0293, found: 403.0278, 405.0238.

*1.7 General procedure for preparation of compounds* ***7a-7f***

To the solution of 2-(3-chlorophenyl)-5-(2-methoxyphenoxy)pyrimidine-4,6-diol (**6a**) in phosphorus oxychloride (20 mL), two drops of N,N-dimethylformamide were added and the mixture was stirred at 80^°^C overnight. After the completion of the reaction, the mixture was slowly added into ice water and extracted with ethyl acetate (100 mL × 3). Then, ethyl acetate solution was dried with anhydrous sodium sulfate and purified by silica gel column to obtain 4,6-dichloro-2-(3-chlorophenyl)-5-(2-methoxyphenoxy)pyrimidine (**7a**).

*1.7.1 4,6-dichloro-2-(3-chlorophenyl)-5-(2-methoxyphenoxy)pyrimidine (****7a****)*

White powder, yield: 83.7%, mp: 127.4-128.6^°^C. HPLC: 99.44%. ^1^H NMR (600 MHz, DMSO-*d_6_*) *δ* 8.27 – 8.22 (m, 2H), 7.72 – 7.67 (m, 1H), 7.62 (t, *J* = 7.9 Hz, 1H), 7.18 (d, *J* = 8.2 Hz, 1H), 7.12 (t, *J* = 7.7 Hz, 1H), 6.92 (d, *J* = 8.1 Hz, 1H), 6.87 (t, *J* = 7.7 Hz, 1H), 3.86 (s, 3H). ^13^C NMR (151 MHz, DMSO-*d_6_*) *δ* 158.14, 155.06 (2C), 148.96, 145.11, 142.18, 136.83, 134.34, 132.12, 131.56, 127.93, 127.13, 124.88, 121.18, 115.65, 113.88, 56.41. ESI-HRMS: calcd. for C_17_H_11_Cl_3_N_2_O_2_ [M+H]^+^ 380.9964, found: 380.9948.

*1.7.2 4,6-dichloro-2-(4-chlorophenyl)-5-(2-methoxyphenoxy)pyrimidine (****7b****)*

White powder, yield: 81.4%, mp: 116.7-117.7^°^C. HPLC: 96.83%. ^1^H NMR (600 MHz, DMSO-*d_6_*) *δ* 8.30 (d, *J* = 8.6 Hz, 2H), 7.64 (d, *J* = 8.6 Hz, 2H), 7.18 (dd, *J* = 8.2, 1.1 Hz, 1H), 7.12 (td, *J* = 8.6, 8.0, 1.4 Hz, 1H), 6.92 (dd, *J* = 8.1, 1.4 Hz, 1H), 6.86 (t, *J* = 7.7 Hz, 1H), 3.86 (s, 3H). ^13^C NMR (151 MHz, DMSO-*d_6_*) *δ* 158.65, 155.05 (2C), 148.95, 145.15, 141.81, 137.33, 133.65, 130.31 (2C), 129.67 (2C), 124.82, 121.18, 115.57, 113.85, 56.39. ESI-HRMS: calcd. for C_17_H_11_Cl_3_N_2_O_2_ [M+H]^+^ 380.9964, found: 380.9946.

*1.7.3 2-(3-bromophenyl)-4,6-dichloro-5-(2-methoxyphenoxy)pyrimidine (****7c****)*

Light red powder, yield: 78.0%, mp: 101.6-102.9^°^C. HPLC: 97.67%. ^1^H NMR (600 MHz, DMSO-*d_6_*) *δ* 8.38 (t, *J* = 1.7 Hz, 1H), 8.29 (d, *J* = 7.9 Hz, 1H), 7.82 (dd, *J* = 8.0, 1.1 Hz, 1H), 7.56 (t, *J* = 8.0 Hz, 1H), 7.18 (dd, *J* = 8.2, 1.3 Hz, 1H), 7.12 (td, *J* = 8.2, 7.8, 1.5 Hz, 1H), 6.92 (dd, *J* = 8.1, 1.4 Hz, 1H), 6.86 (td, *J* = 7.8, 1.4 Hz, 1H), 3.86 (s, 3H). ^13^C NMR (151 MHz, DMSO-*d_6_*) *δ* 158.04, 155.05 (2C), 148.96, 145.11, 142.17, 137.01, 135.00, 131.80, 130.86, 127.50, 124.89, 122.75, 121.18, 115.65, 113.88, 56.41. ESI-HRMS: calcd. for C_17_H_11_BrCl_2_N_2_O_2_ [M+H]^+^ 424.9459, found: 424.9441, 426.9403.

*1.7.4 2-(4-bromophenyl)-4,6-dichloro-5-(2-methoxyphenoxy)pyrimidine (****7d****)*

White powder, yield: 84.9%, mp: 103.0-105.7^°^C. HPLC: 98.39%. ^1^H NMR (600 MHz, DMSO-*d_6_*) *δ* 8.22 (d, *J* = 8.6 Hz, 2H), 7.79 (d, *J* = 8.6 Hz, 2H), 7.17 (dd, *J* = 8.2, 1.4 Hz, 1H), 7.11 (td, *J* = 7.8, 1.5 Hz, 1H), 6.92 (dd, *J* = 8.1, 1.5 Hz, 1H), 6.85 (t, *J* = 7.7 Hz, 1H), 3.86 (s, 3H). ^13^C NMR (151 MHz, DMSO-*d_6_*) *δ* 158.78, 155.07 (2C), 148.94, 145.13, 141.84, 134.01, 132.63 (2C), 130.50 (2C), 126.38, 124.82, 121.18, 115.56, 113.85, 56.39. ESI-HRMS: calcd. for C_17_H_11_BrCl_2_N_2_O_2_ [M+H]^+^ 424.9459, found: 424.9442, 426.9404.

*1.7.5 4,6-dichloro-2-(3-iodophenyl)-5-(2-methoxyphenoxy)pyrimidine (****7e****)*

Light red powder, yield: 67.2%, mp: 151.7-153.1^°^C. HPLC: 97.38%. ^1^H NMR (600 MHz, DMSO-*d_6_*) *δ* 8.58 (t, *J* = 1.6 Hz, 1H), 8.30 (d, *J* = 7.9 Hz, 1H), 7.98 (d, *J* = 8.4 Hz, 1H), 7.39 (t, *J* = 7.9 Hz, 1H), 7.18 (dd, *J* = 8.2, 1.4 Hz, 1H), 7.12 (td, *J* = 8.2, 7.8, 1.5 Hz, 1H), 6.92 (dd, *J* = 8.1, 1.5 Hz, 1H), 6.86 (td, *J* = 7.8, 1.5 Hz, 1H), 3.86 (s, 3H). ^13^C NMR (151 MHz, DMSO-*d_6_*) *δ* 158.01, 155.01 (2C), 148.95, 145.12, 142.06, 140.79, 136.81, 136.77, 131.69, 127.83, 124.87, 121.19, 115.63, 113.88, 95.73, 56.41. ESI-HRMS: calcd. for C_17_H_11_Cl_2_IN_2_O_2_ [M+H]^+^ 472.9321, found: 472.9298.

*1.7.6 4,6-dichloro-2-(4-iodophenyl)-5-(2-methoxyphenoxy)pyrimidine (****7f****)*

Light red powder, yield: 71.1%, mp: 138.6-139.9^°^C. HPLC: 99.85%. ^1^H NMR (600 MHz, DMSO-*d_6_*) *δ* 8.06 (d, *J* = 8.6 Hz, 2H), 7.96 (d, *J* = 8.6 Hz, 2H), 7.17 (dd, *J* = 8.2, 1.4 Hz, 1H), 7.11 (td, *J* = 7.8, 1.5 Hz, 1H), 6.91 (dd, *J* = 8.1, 1.5 Hz, 1H), 6.85 (td, *J* = 7.8, 1.5 Hz, 1H), 3.86 (s, 3H). ^13^C NMR (151 MHz, DMSO-*d_6_*) *δ* 159.06, 155.05 (2C), 148.94, 145.13, 141.83, 138.50 (2C), 134.32, 130.33 (2C), 124.82, 121.18, 115.56, 113.85, 100.50, 56.40. ESI-HRMS: calcd. for C_17_H_11_Cl_2_IN_2_O_2_ [M+H]^+^ 472.9321, found: 472.9298.

***2. NMR spectra and mass spectrum***

*2.1 2-(3-chlorophenyl)-5-(2-methoxyphenoxy)pyrimidine-4,6-diol (****6a****)*


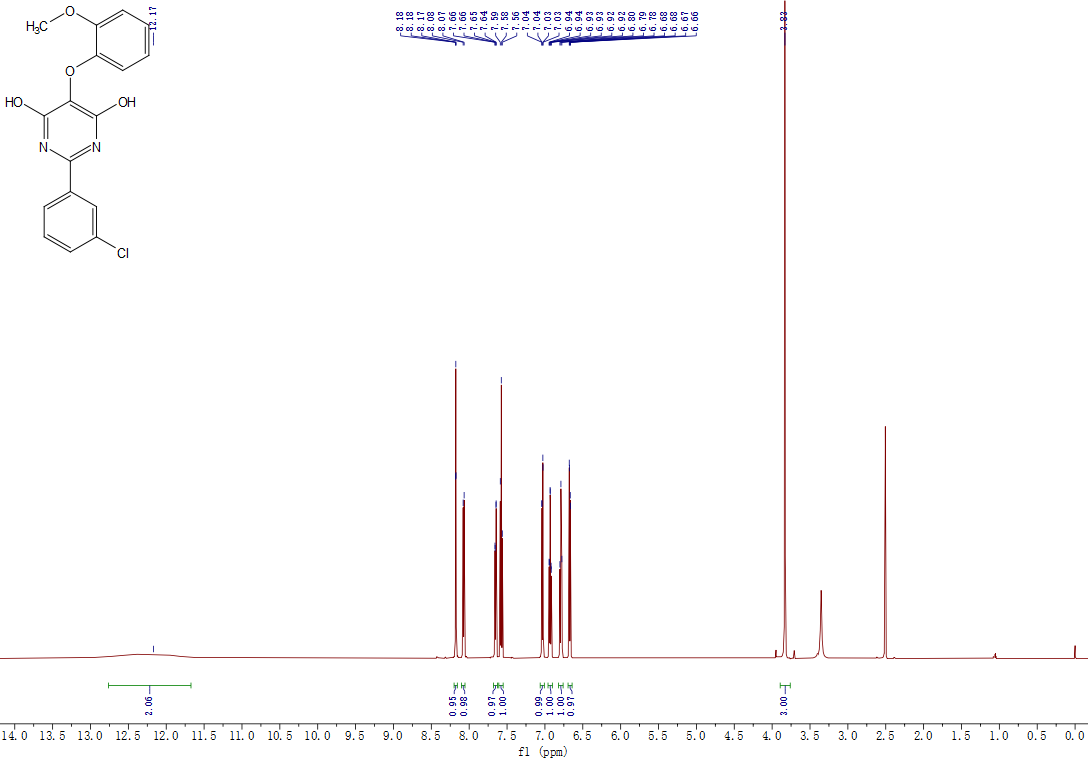


**Figure S1. ^1^H-NMR spectra of 6a**


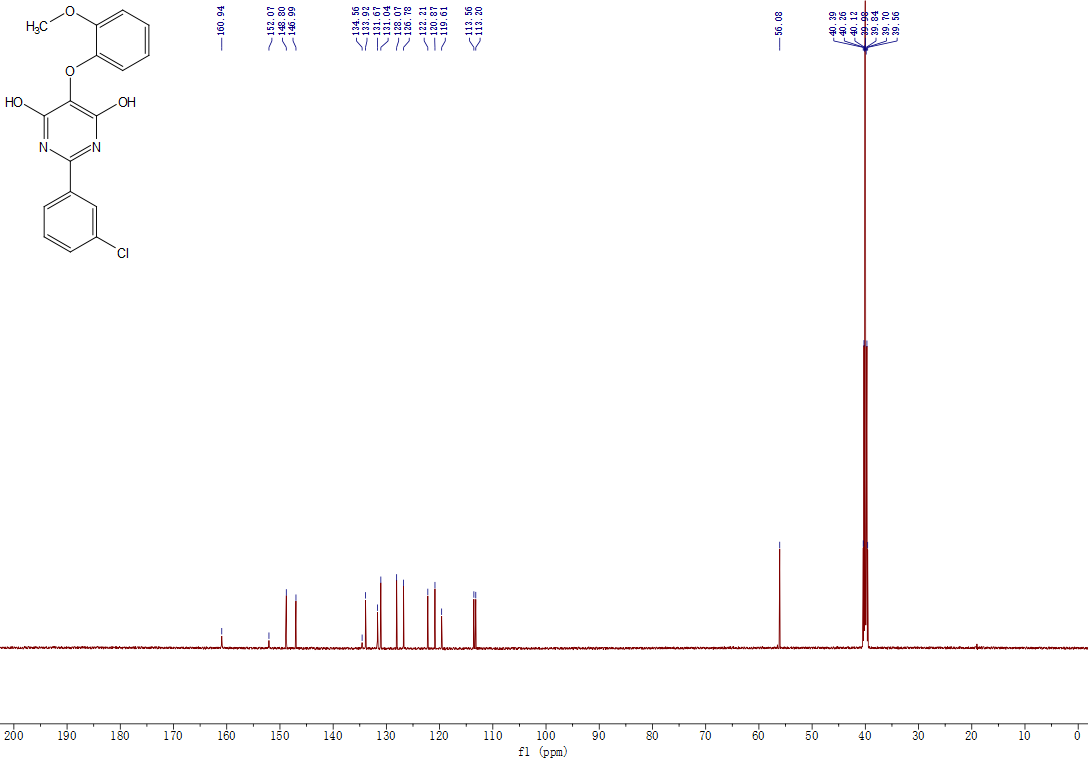


**Figure S2. ^13^C-NMR spectra of 6a**


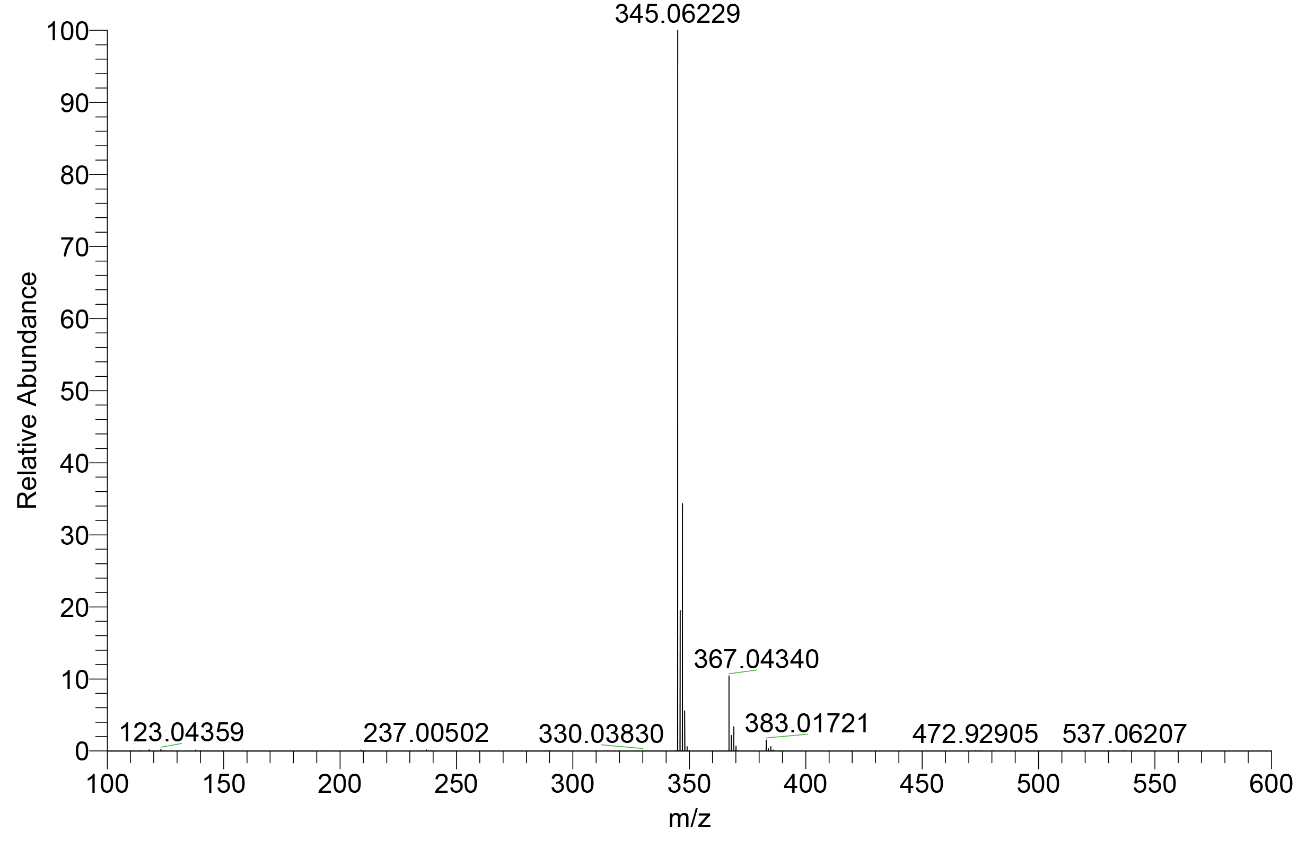


**Figure S3. Mass spectrum of 6a**

*
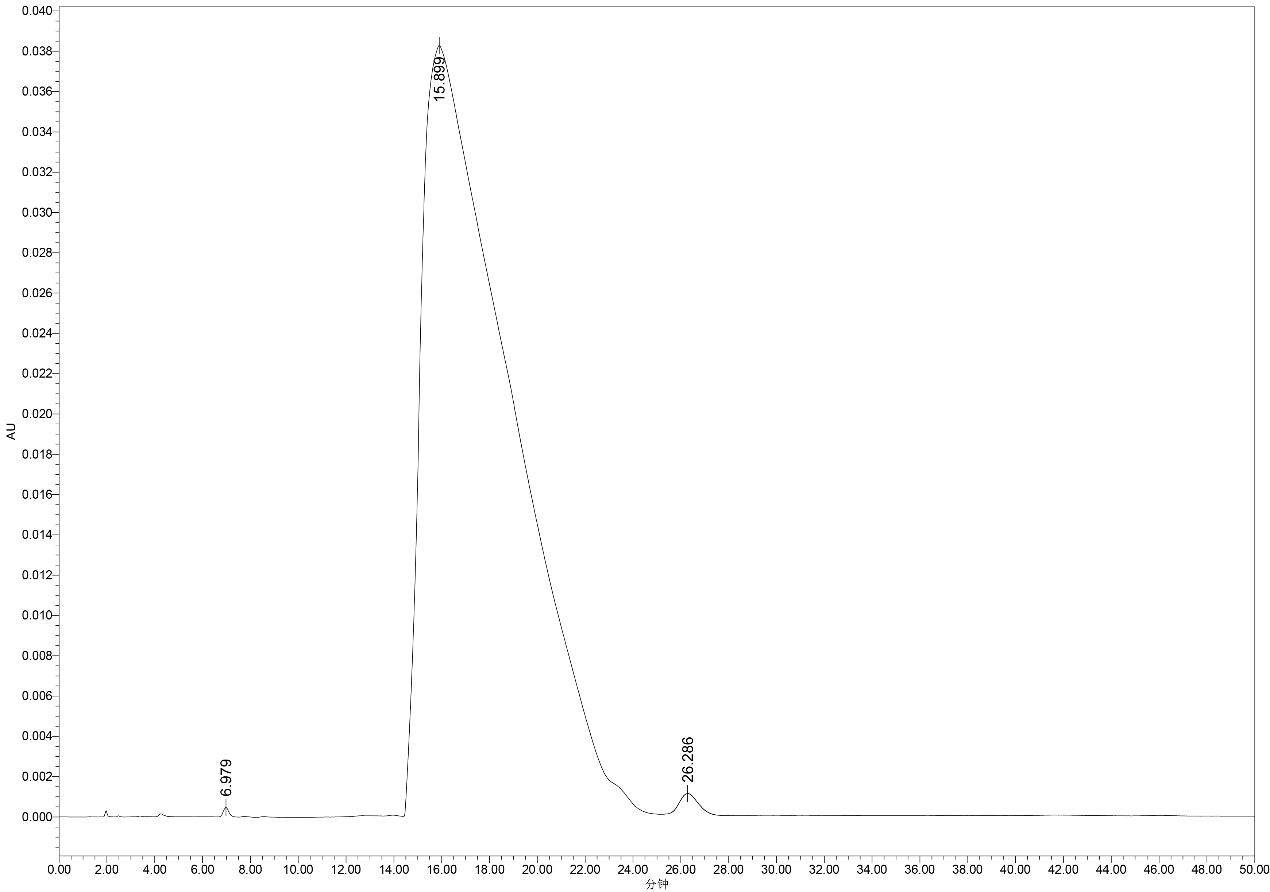
*

**Table S1.** HPLC analysis of **6a** (1.0mL/min, MeOH: Water = 60:40 v/v).

| Peak | Retention time | Peak area | Peak area % | Peak start | Peak end |
| --- | --- | --- | --- | --- | --- |
| 1 | 6.979 | 16575 | 0.17 | 2.75 | 8.217 |
| 2 | 15.899 | 9881530 | 99.34 | 14.417 | 24.283 |
| 3 | 26.286 | 49296 | 0.5 | 25.633 | 27.417 |

*2.2 2-(4-chlorophenyl)-5-(2-methoxyphenoxy)pyrimidine-4,6-diol (****6b****)*


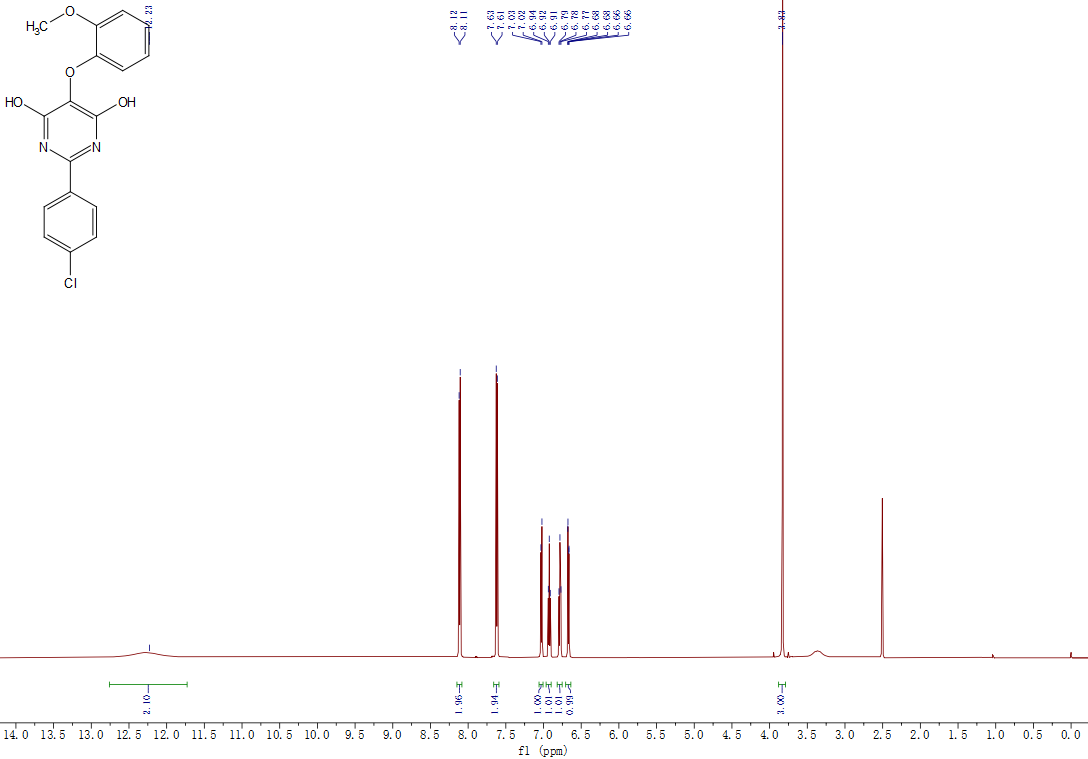


**Figure S4. ^1^H-NMR spectra of 6b**


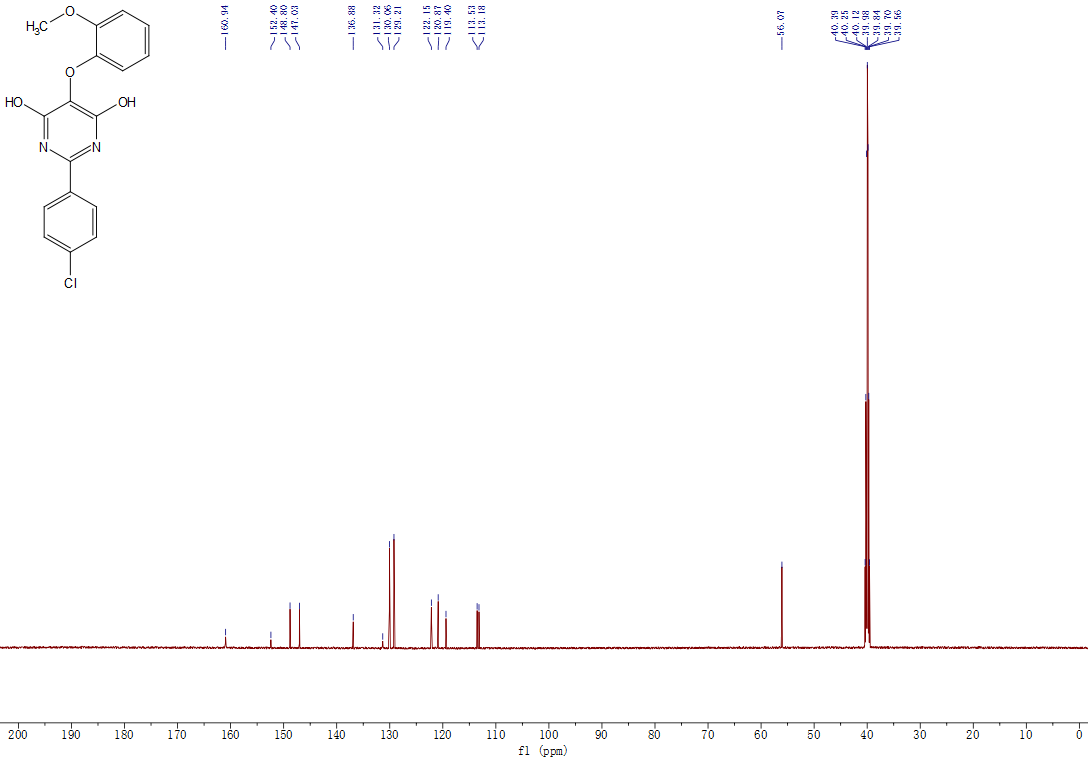


**Figure S5. ^13^C-NMR spectra of 6b**


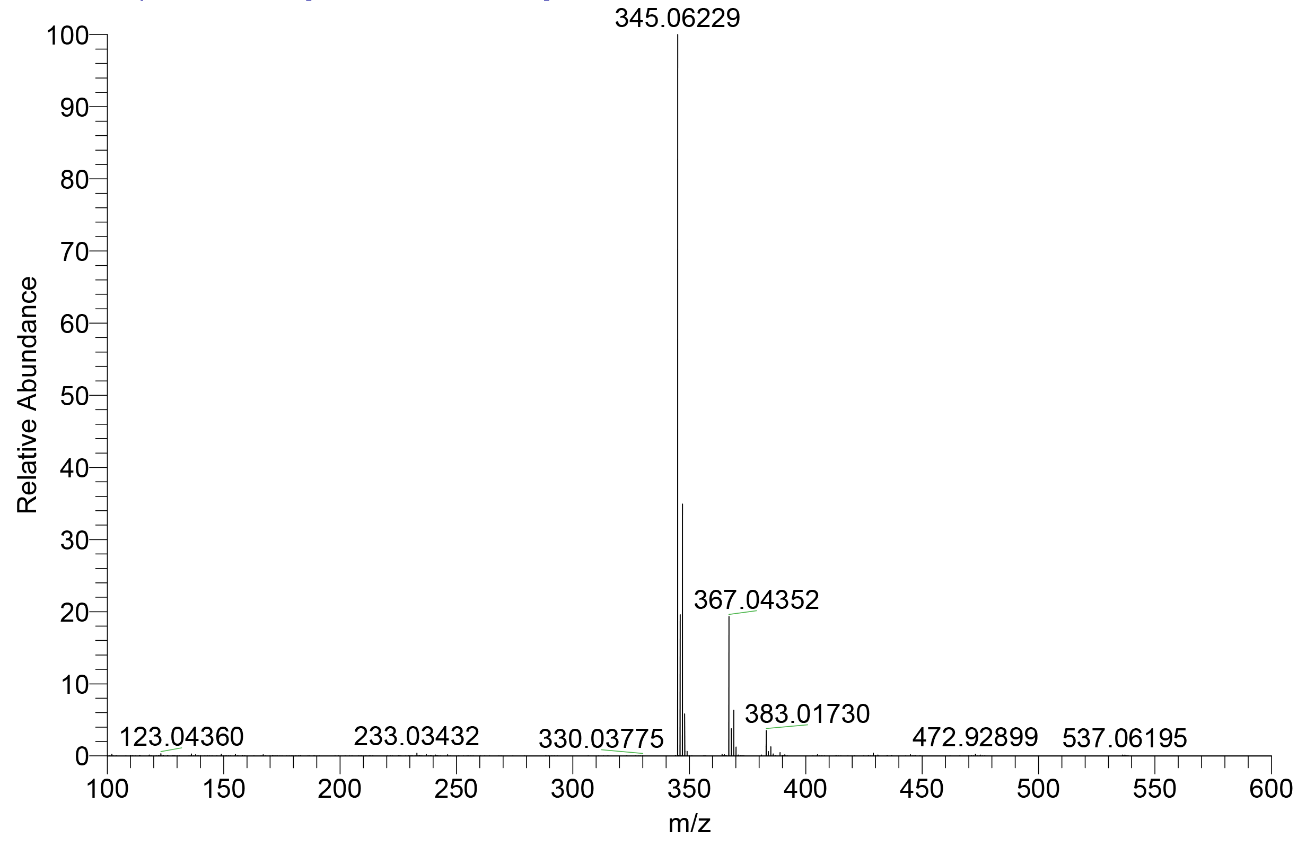


**Figure S6. Mass spectrum of 6b**

*
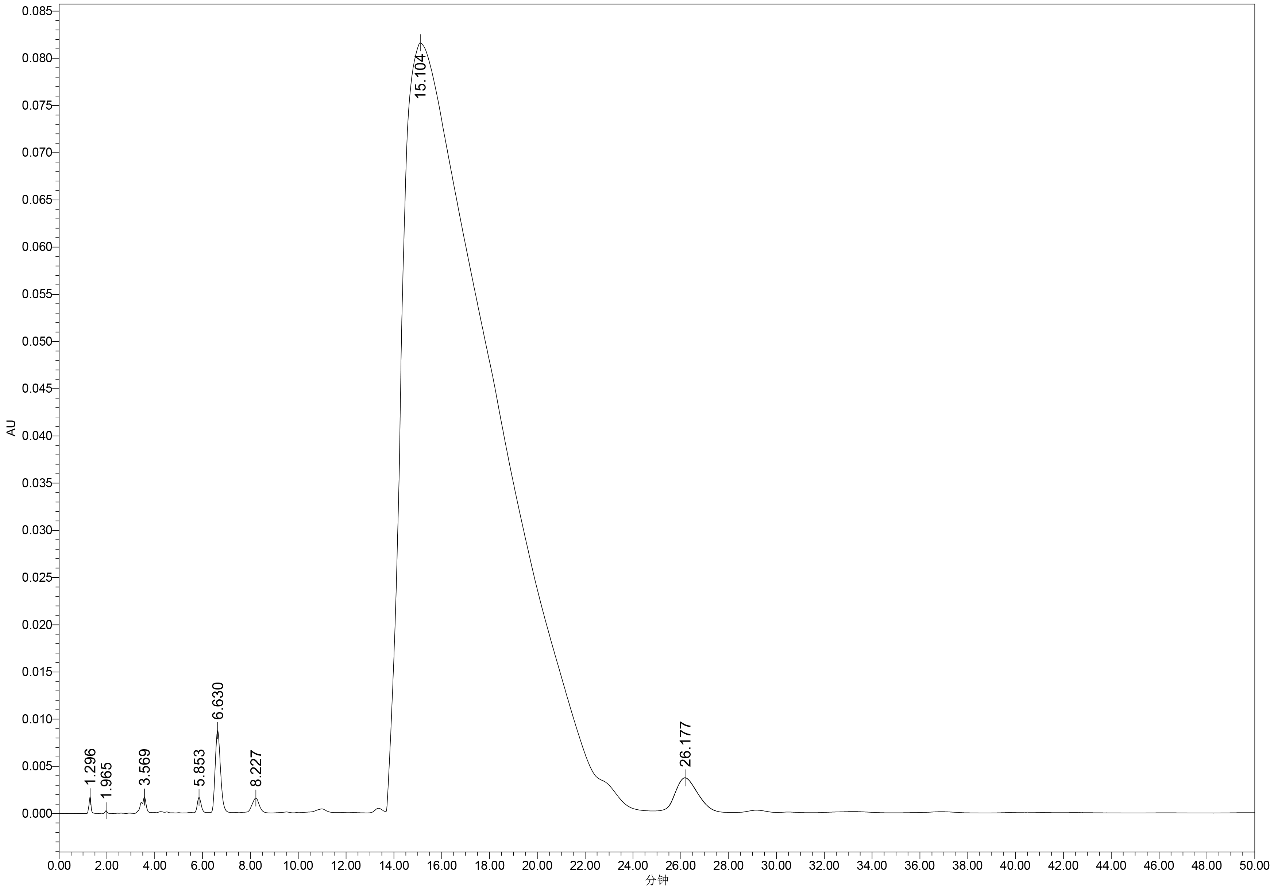
*

**Table S2.** HPLC analysis of **6b** (1.0mL/min, MeOH: Water = 60:40 v/v).

| Peak | Retention time | Peak area | Peak area % | Peak start | Peak end |
| --- | --- | --- | --- | --- | --- |
| 1 | 1.296 | 10372 | 0.05 | 1.15 | 1.483 |
| 2 | 1.965 | 1697 | 0.01 | 1.85 | 2.25 |
| 3 | 3.569 | 23907 | 0.11 | 3.15 | 3.817 |
| 4 | 5.853 | 17941 | 0.08 | 5.633 | 6.3 |
| 5 | 6.63 | 131937 | 0.59 | 6.317 | 7.05 |
| 6 | 8.227 | 27842 | 0.13 | 7.867 | 8.55 |
| 7 | 15.104 | 21780684 | 98.16 | 13.667 | 24.033 |
| 8 | 26.177 | 194529 | 0.88 | 25.433 | 27.45 |

*2.3 2-(3-bromophenyl)-5-(2-methoxyphenoxy)pyrimidine-4,6-diol (****6c****)*


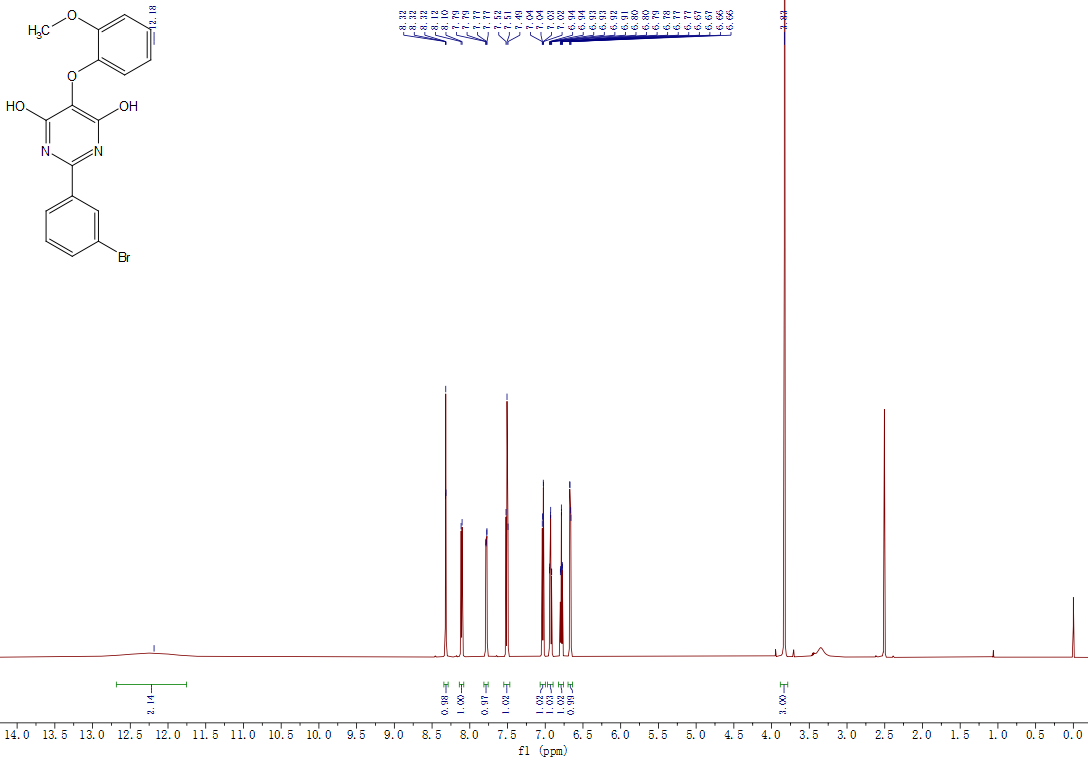


**Figure S7. ^1^H-NMR spectra of 6c**


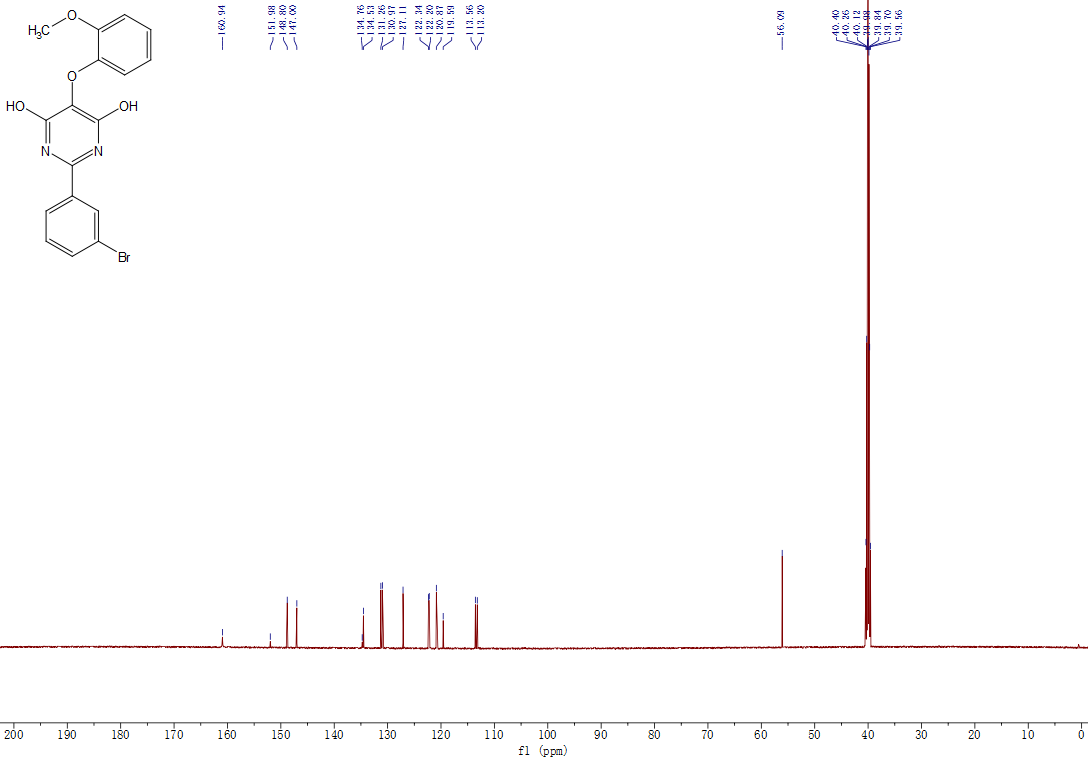


**Figure S8.** **^13^C-NMR spectra of 6c**


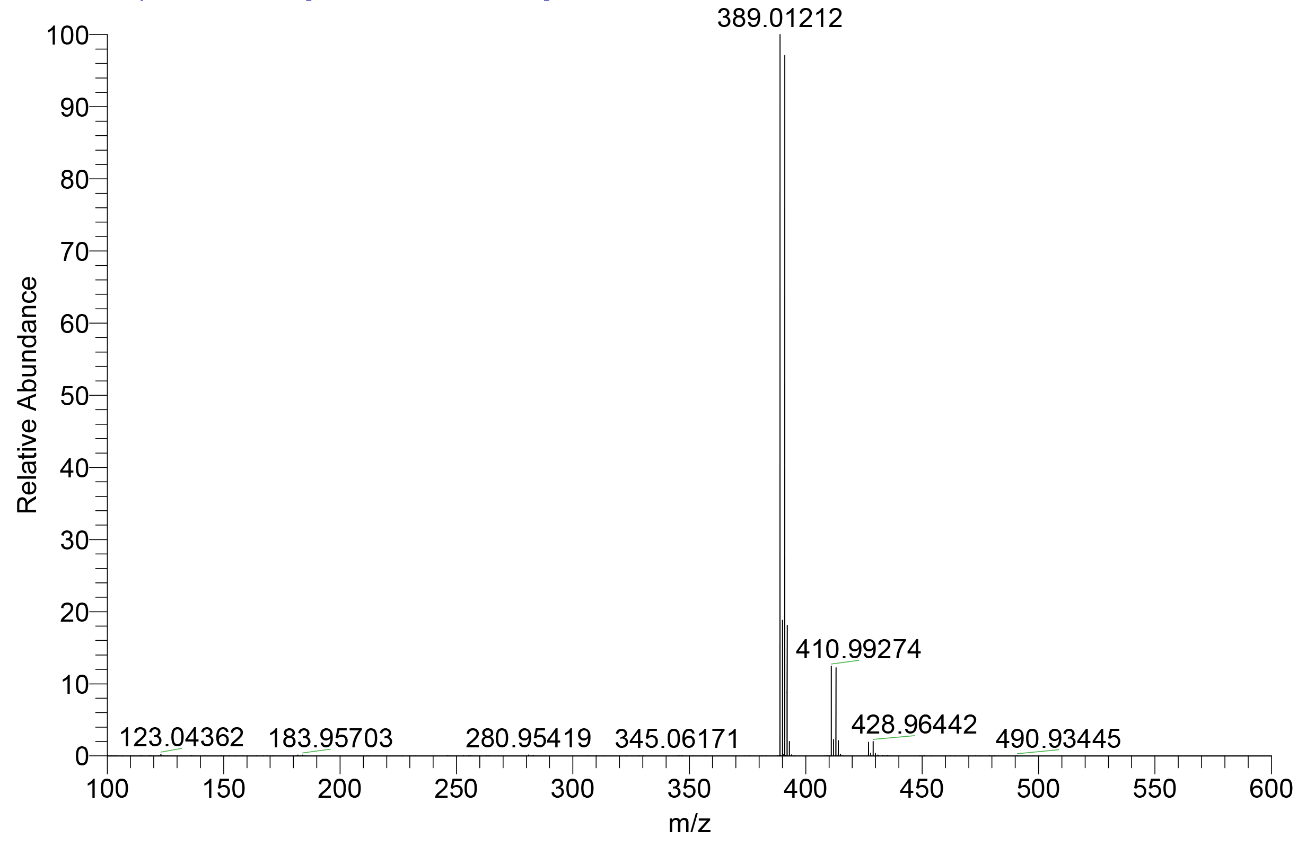


**Figure S9. Mass spectrum of 6c**

*
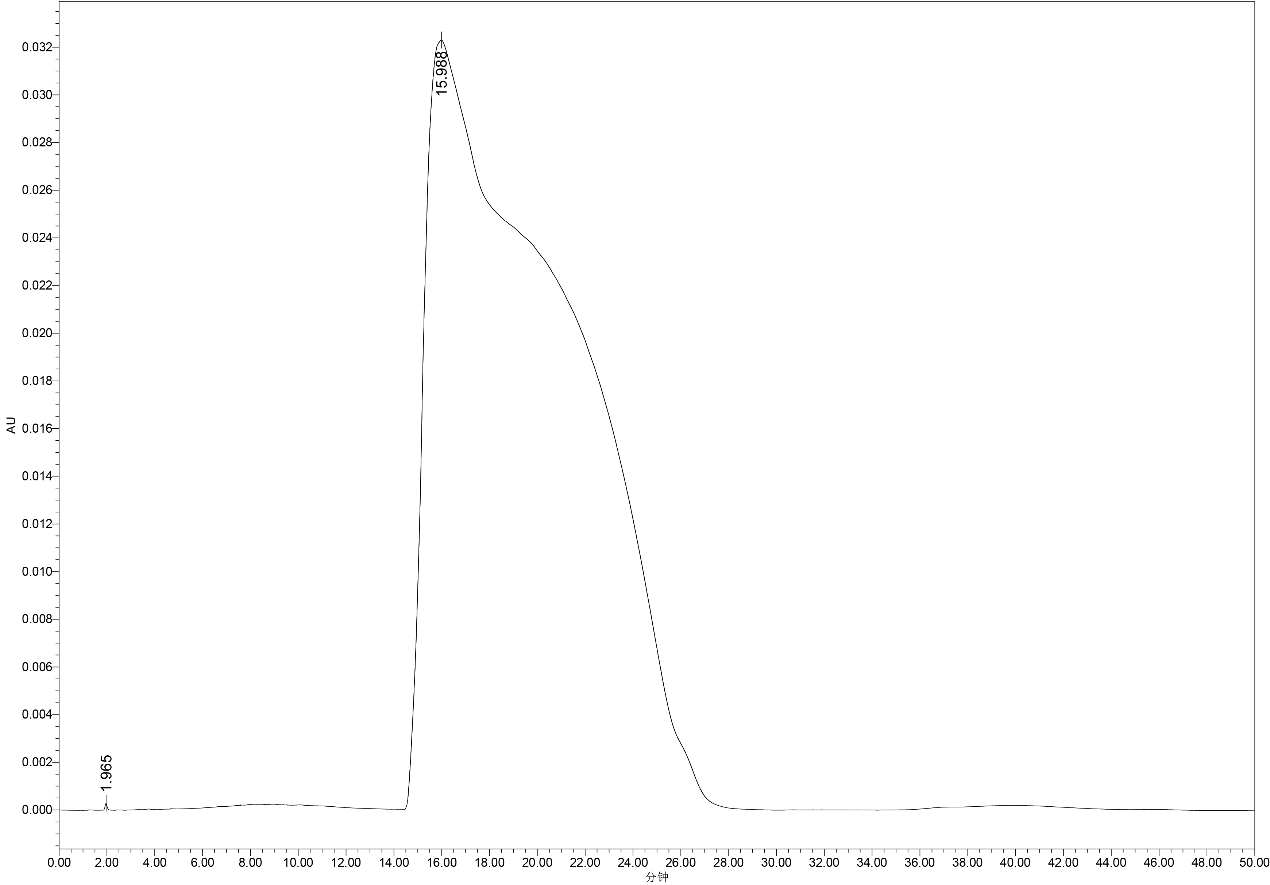
*

**Table S3.** HPLC analysis of **6c** (1.0mL/min, MeOH: Water = 60:40 v/v).

| Peak | Retention time | Peak area | Peak area % | Peak start | Peak end |
| --- | --- | --- | --- | --- | --- |
| 1 | 1.965 | 1808 | 0.01 | 1.833 | 2.317 |
| 2 | 15.988 | 13223548 | 99.99 | 1283 | 27.317 |

*2.4 2-(4-bromophenyl)-5-(2-methoxyphenoxy)pyrimidine-4,6-diol (****6d****)*


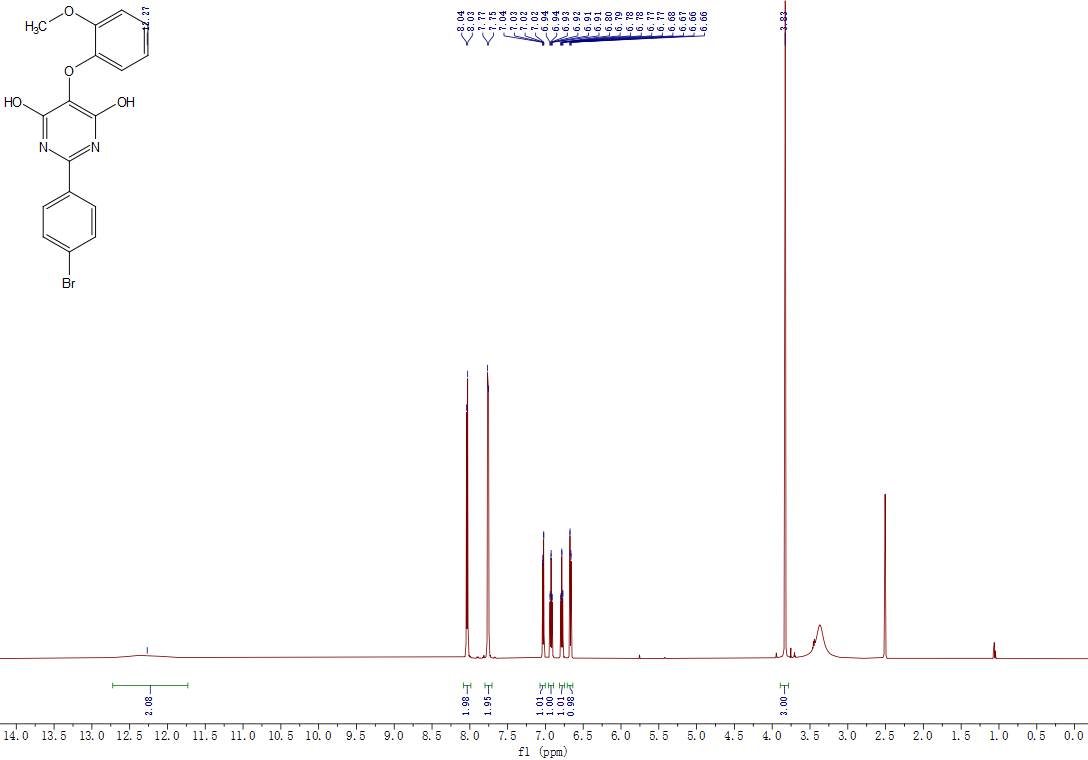


**Figure S10. ^1^H-NMR spectra of 6d**


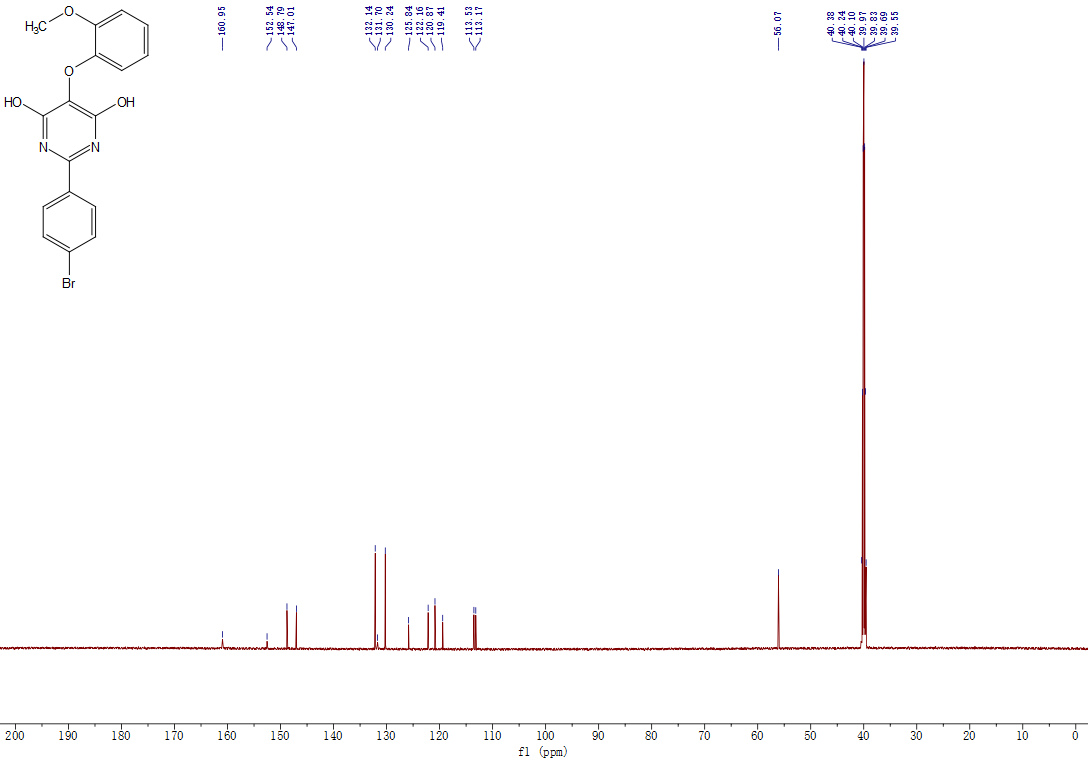


**Figure S11.^13^** **C-NMR spectra of 6d**


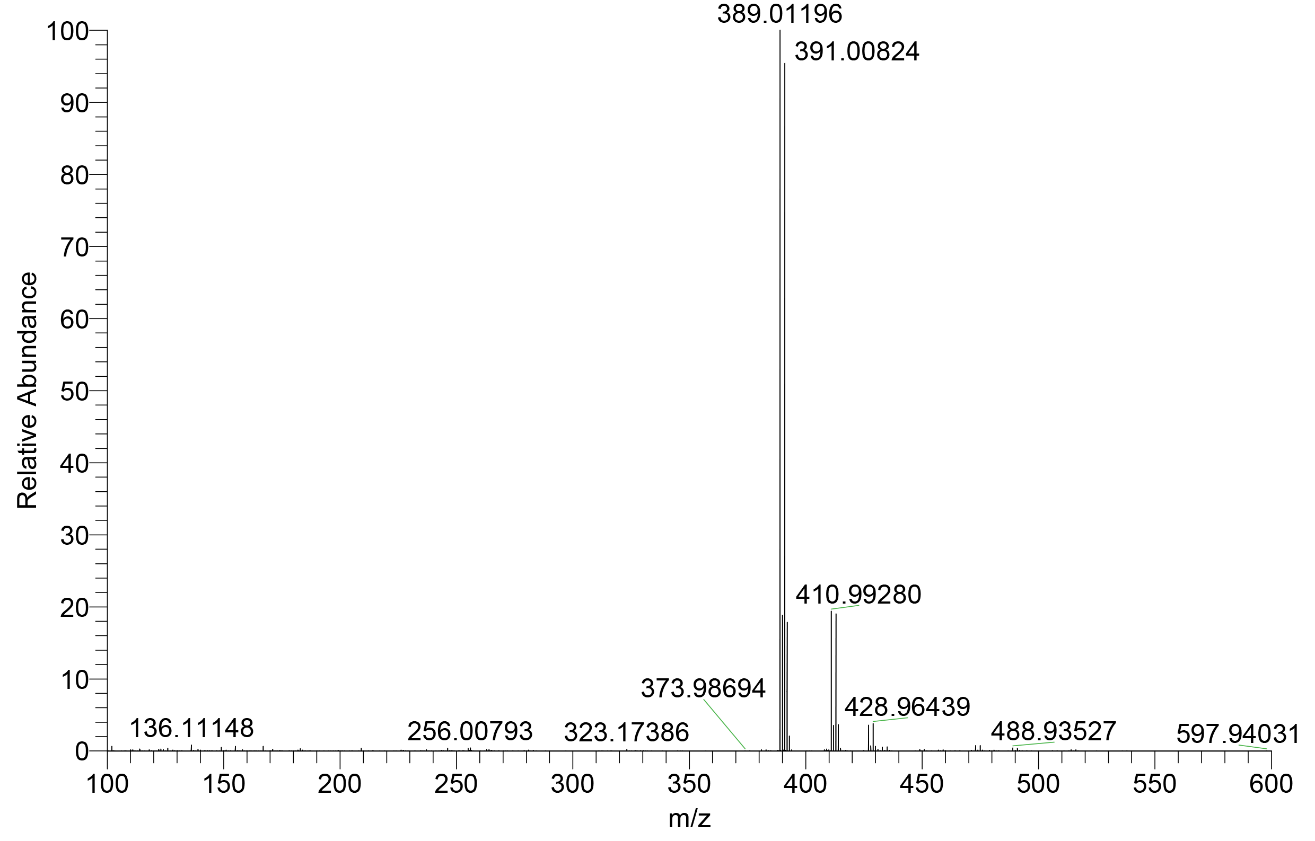


**Figure S12. Mass spectrum of 6d**

*
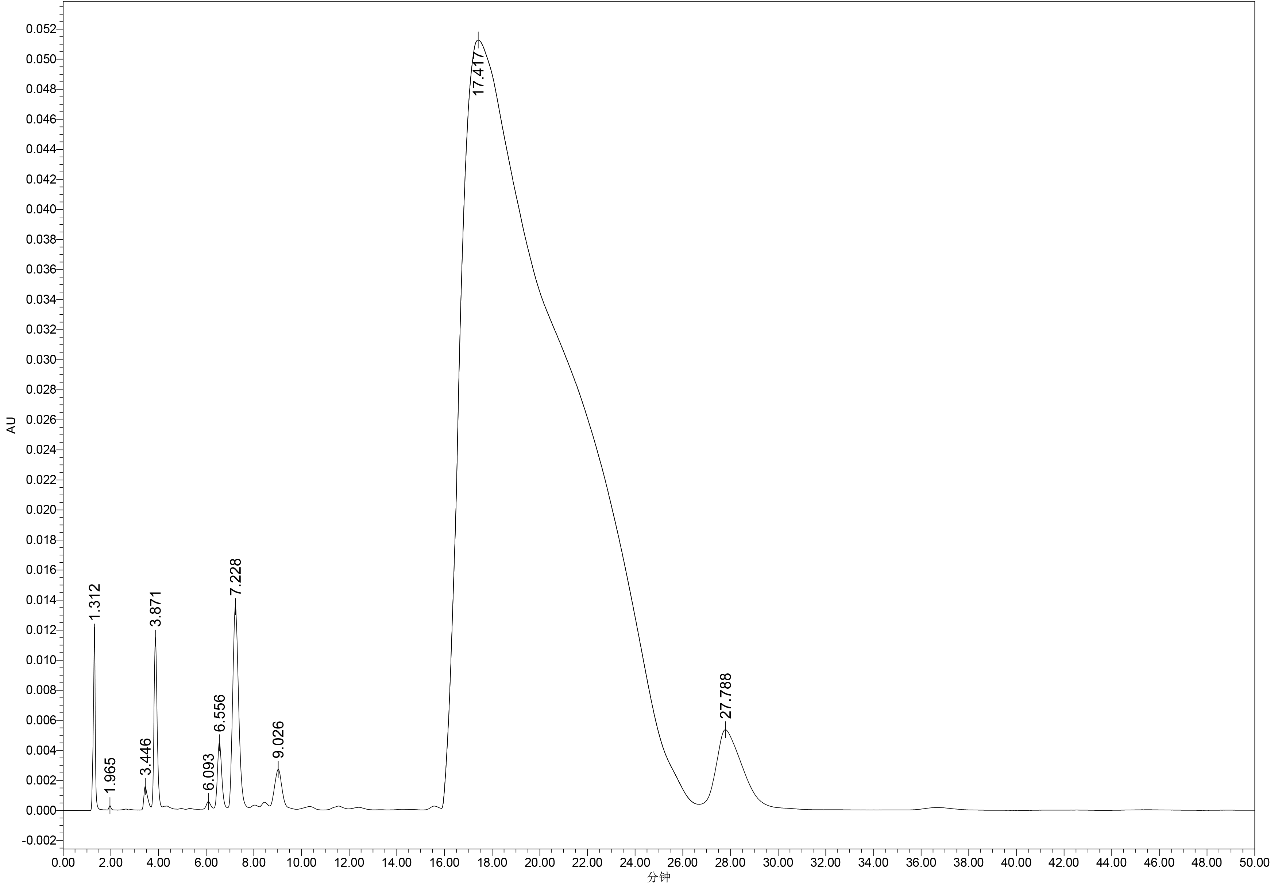
*

**Table S4.** HPLC analysis of **6d** (1.0mL/min, MeOH: Water = 60:40 v/v).

| Peak | Retention time | Peak area | Peak area % | Peak start | Peak end |
| --- | --- | --- | --- | --- | --- |
| 1 | 1.312 | 67815 | 0.4 | 1.15 | 1.55 |
| 2 | 1.965 | 1820 | 0.01 | 1.85 | 2.283 |
| 3 | 3.446 | 16110 | 0.1 | 3.267 | 3.683 |
| 4 | 3.871 | 101951 | 0.61 | 3.683 | 4.167 |
| 5 | 6.093 | 5495 | 0.03 | 5.9 | 6.317 |
| 6 | 6.556 | 53638 | 0.32 | 6.317 | 6.9 |
| 7 | 7.228 | 228198 | 1.36 | 6.9 | 7.717 |
| 8 | 9.026 | 49121 | 0.29 | 8.667 | 9.417 |
| 9 | 17.417 | 15947223 | 95.23 | 15.867 | 26.533 |
| 10 | 27.788 | 274930 | 1.64 | 27 | 28.983 |

*2.5 2-(3-iodophenyl)-5-(2-methoxyphenoxy)pyrimidine-4,6-diol (****6e****)*


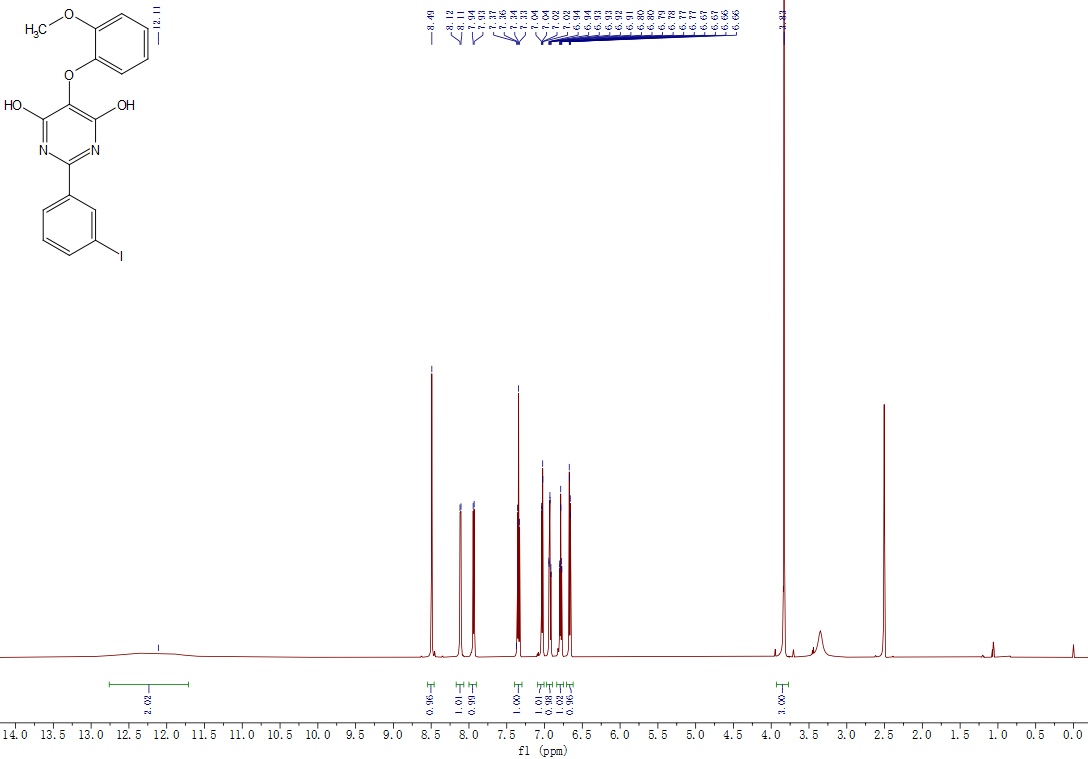


**Figure S13.** **^1^H-NMR spectra of 6e**


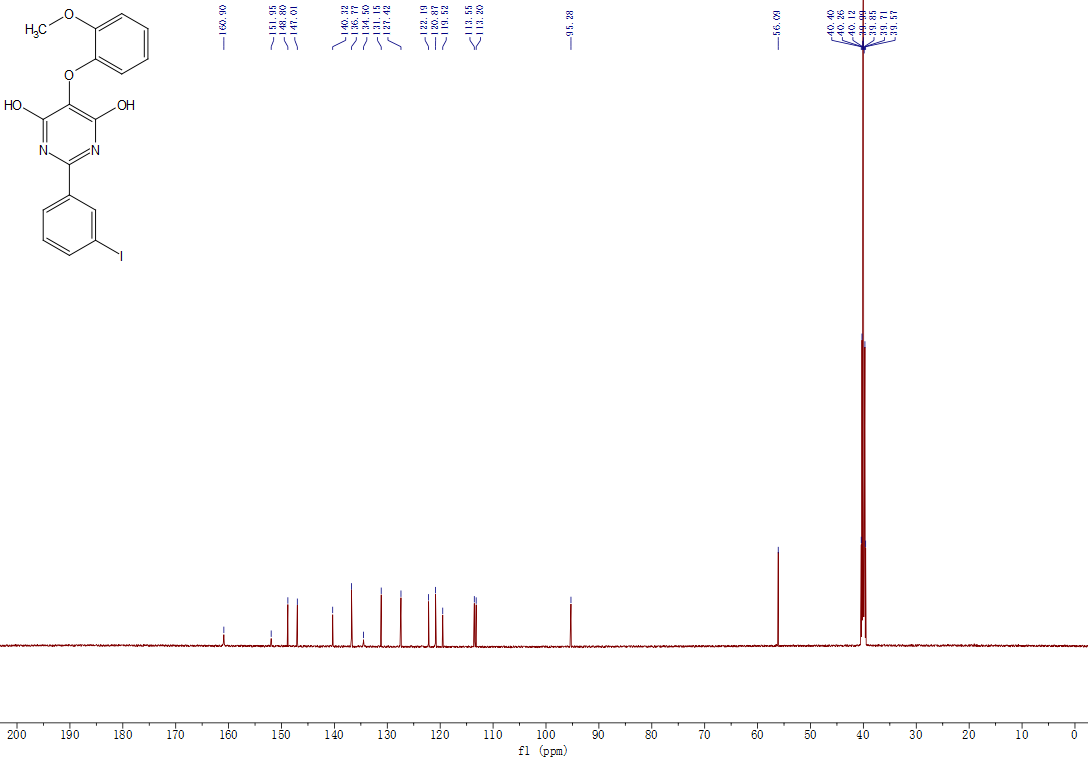


**Figure S14. ^13^C-NMR spectra of 6e**


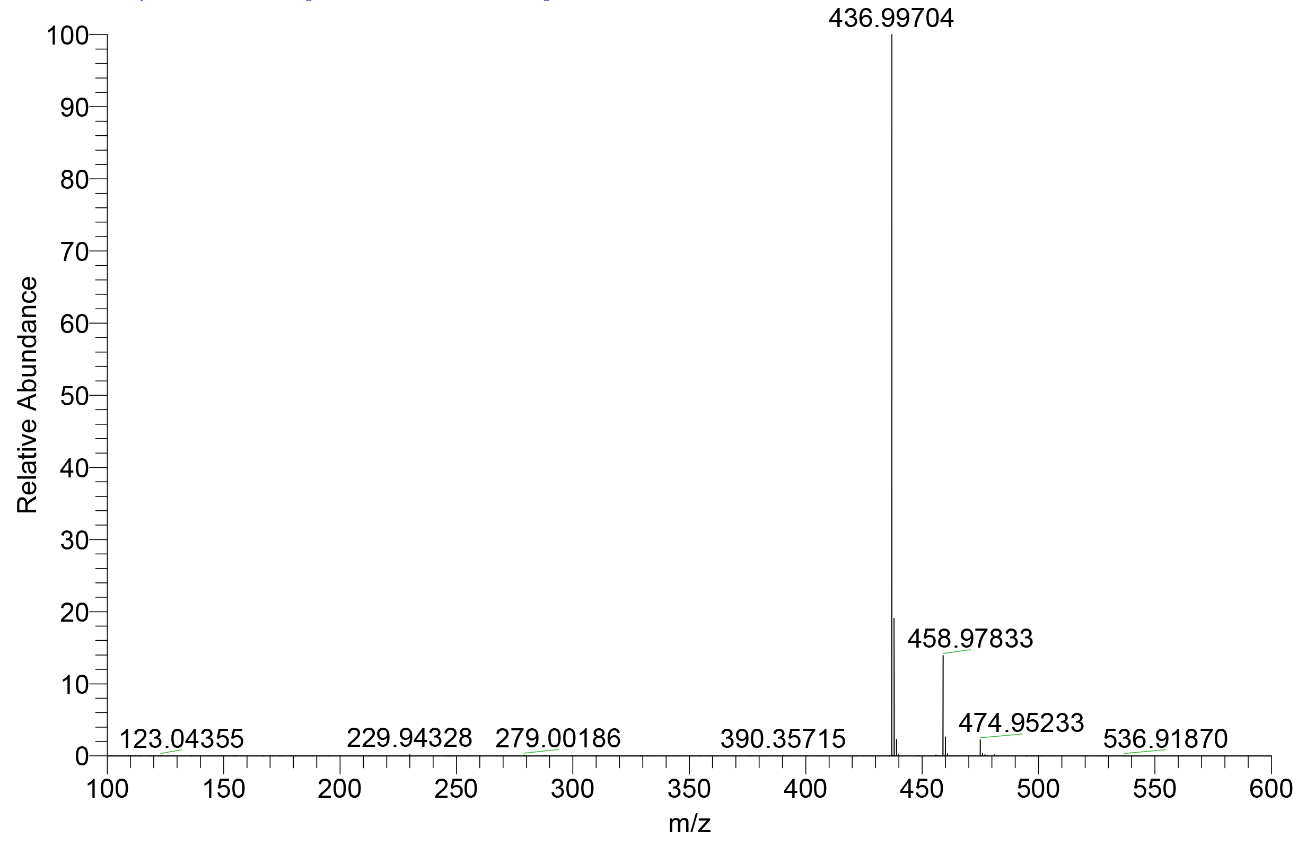


**Figure S15. Mass spectrum of 6e**

*
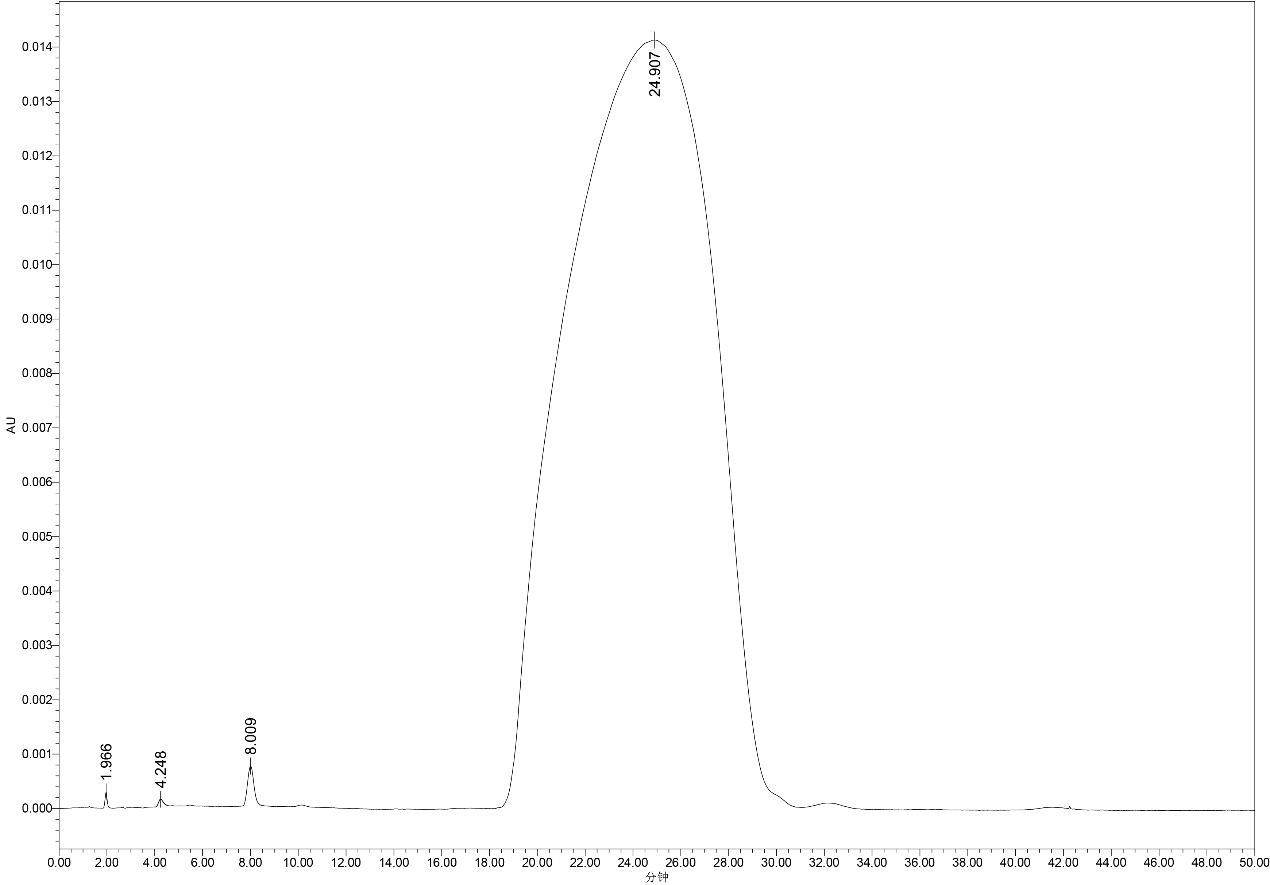
*

**Table S5.** HPLC analysis of **6e** (1.0mL/min, MeOH: Water = 60:40 v/v).

| Peak | Retention time | Peak area | Peak area % | Peak start | Peak end |
| --- | --- | --- | --- | --- | --- |
| 1 | 1.966 | 1718 | 0.03 | 1.85 | 2.117 |
| 2 | 4.248 | 1601 | 0.03 | 4.083 | 217 |
| 3 | 8.009 | 9960 | 0.17 | 7.767 | 8.25 |
| 4 | 24.907 | 5983372 | 99.78 | 18.3 | 30.667 |

*2.6 2-(4-iodophenyl)-5-(2-methoxyphenoxy)pyrimidine-4,6-diol (****6f****)*


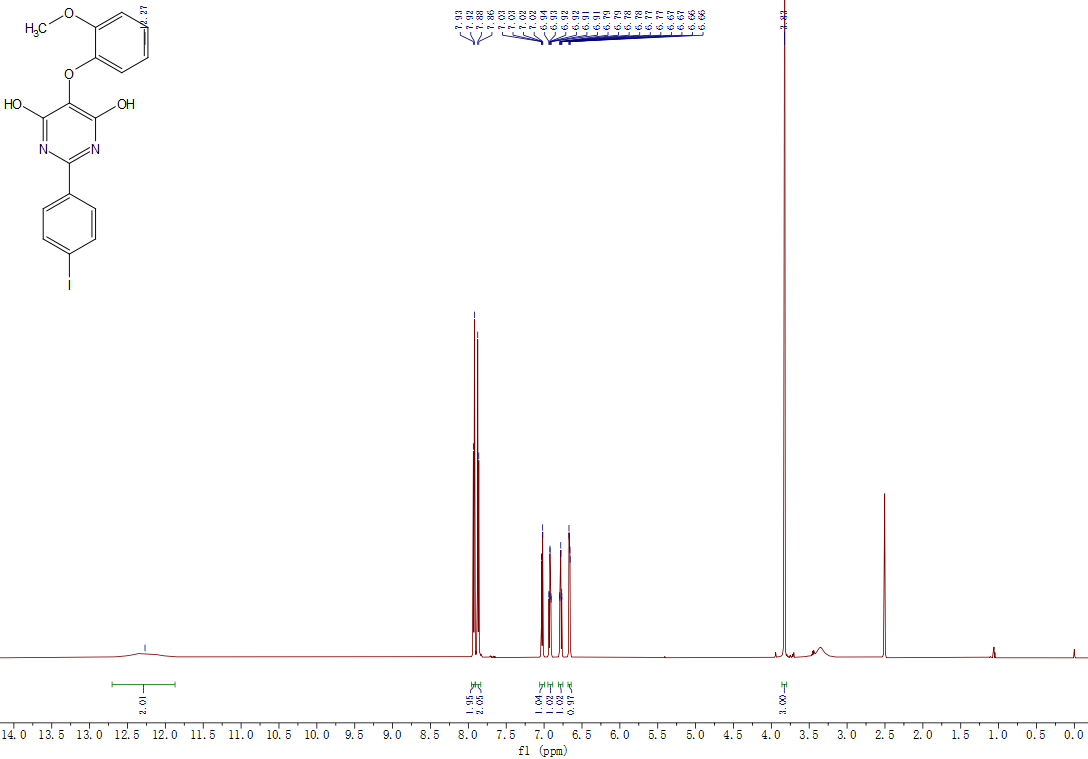


**Figure S16.** **^1^H-NMR spectra of 6f**


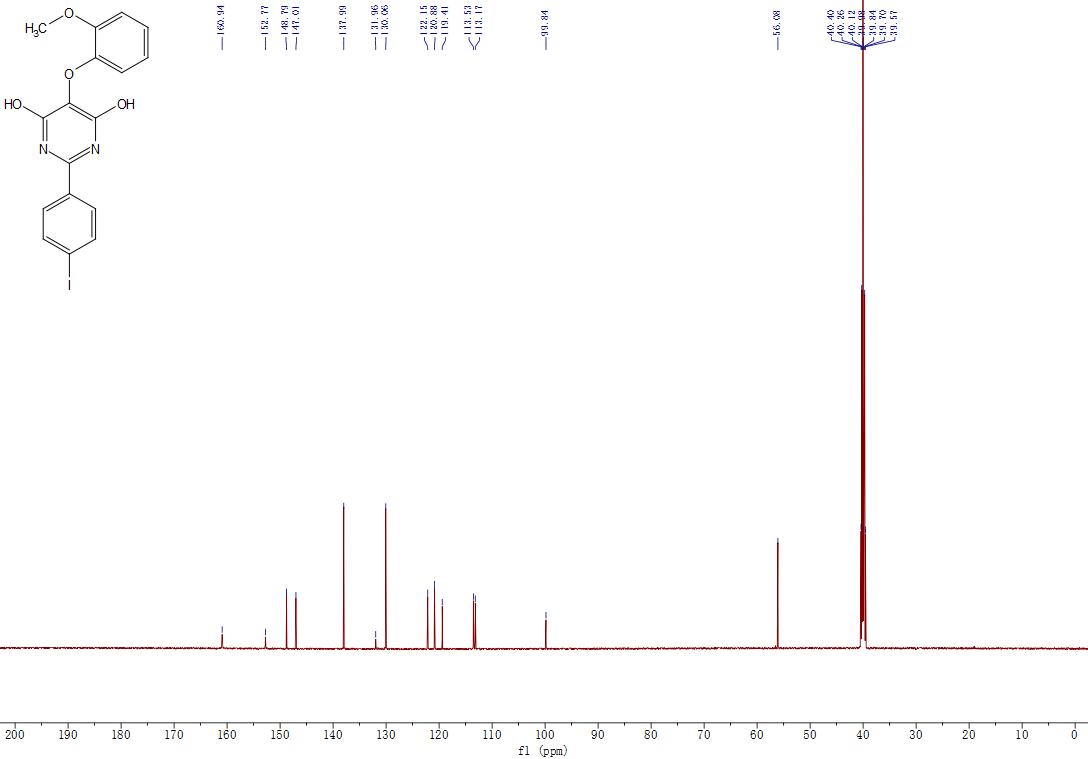


**Figure S17.** **^13^C-NMR spectra of 6f**


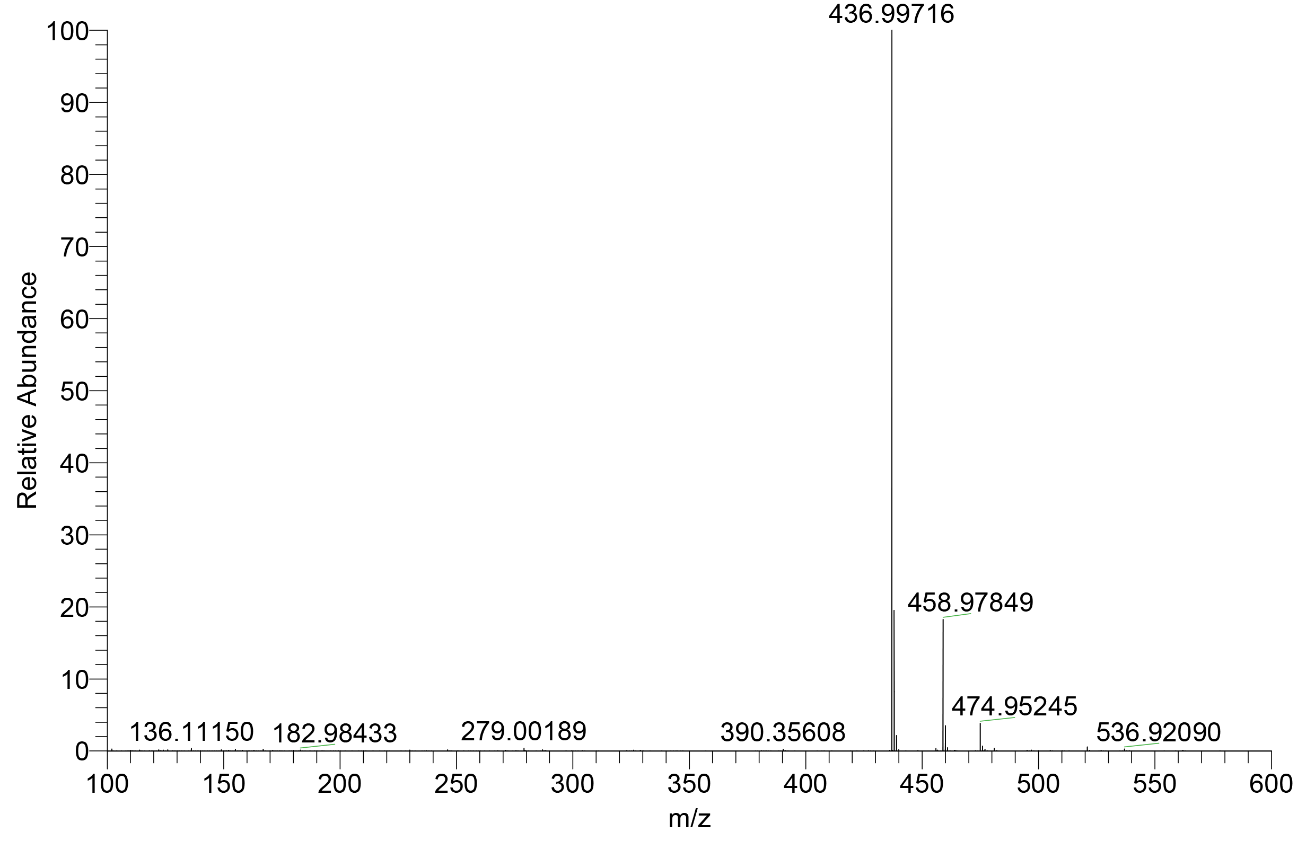
**Figure S18. Mass spectrum of 6f**

*
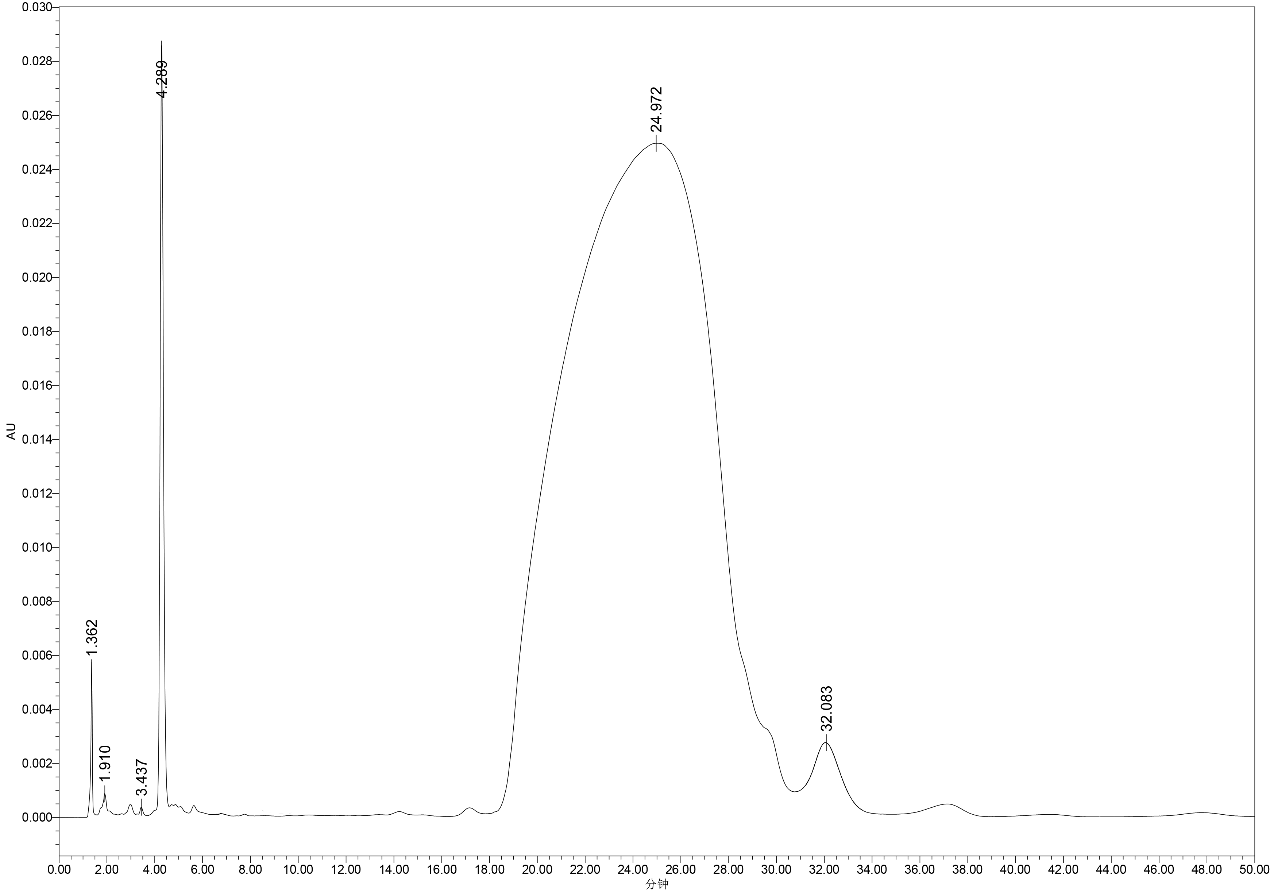
*

**Table S6.** HPLC analysis of **6f** (1.0mL/min, MeOH: Water = 60:40 v/v).

| Peak | Retention time | Peak area | Peak area % | Peak start | Peak end |
| --- | --- | --- | --- | --- | --- |
| 1 | 1.362 | 24776 | 0.23 | 1.167 | 1.517 |
| 2 | 1.91 | 4123 | 0.04 | 1.767 | 2.05 |
| 3 | 3.437 | 2132 | 0.02 | 3.317 | 3.65 |
| 4 | 4.289 | 274355 | 2.53 | 3.75 | 4.617 |
| 5 | 24.972 | 10408583 | 95.98 | 18.533 | 30.667 |
| 6 | 32.083 | 131011 | 1.21 | 31.183 | 33.6 |

*2.7 2-(3-chlorobenzyl)-5-(2-methoxyphenoxy)pyrimidine-4,6-diol (****6g****)*


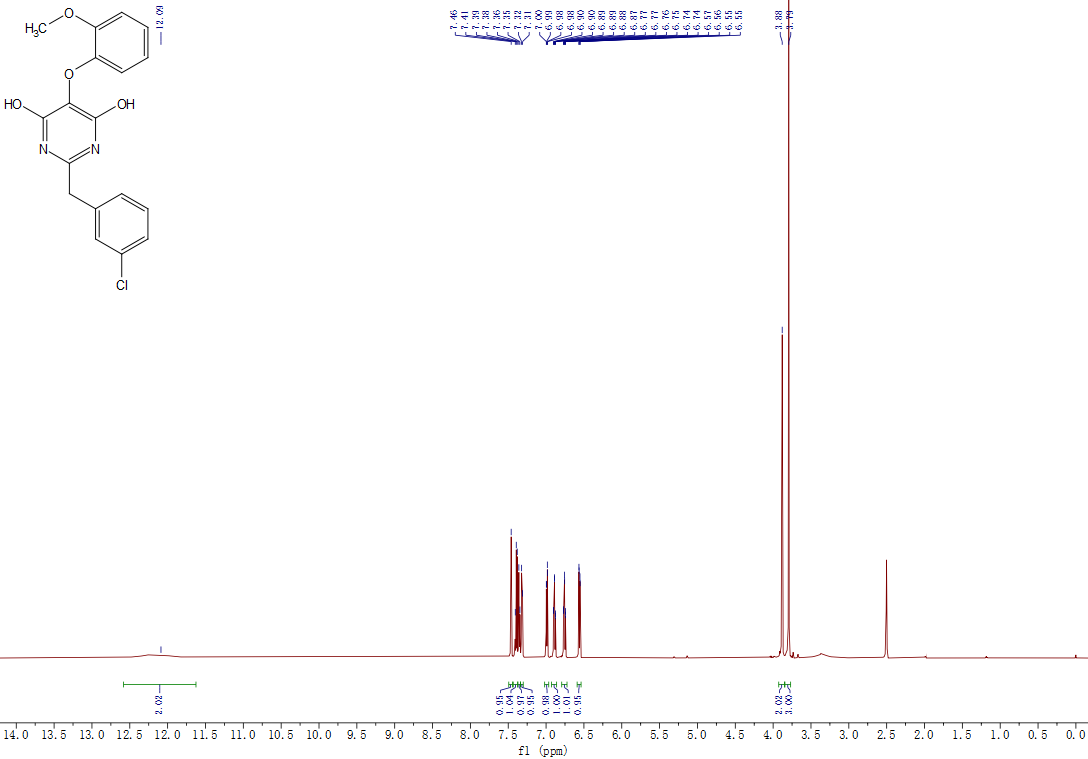


**Figure S19.** **^1^H-NMR spectra of 6g**


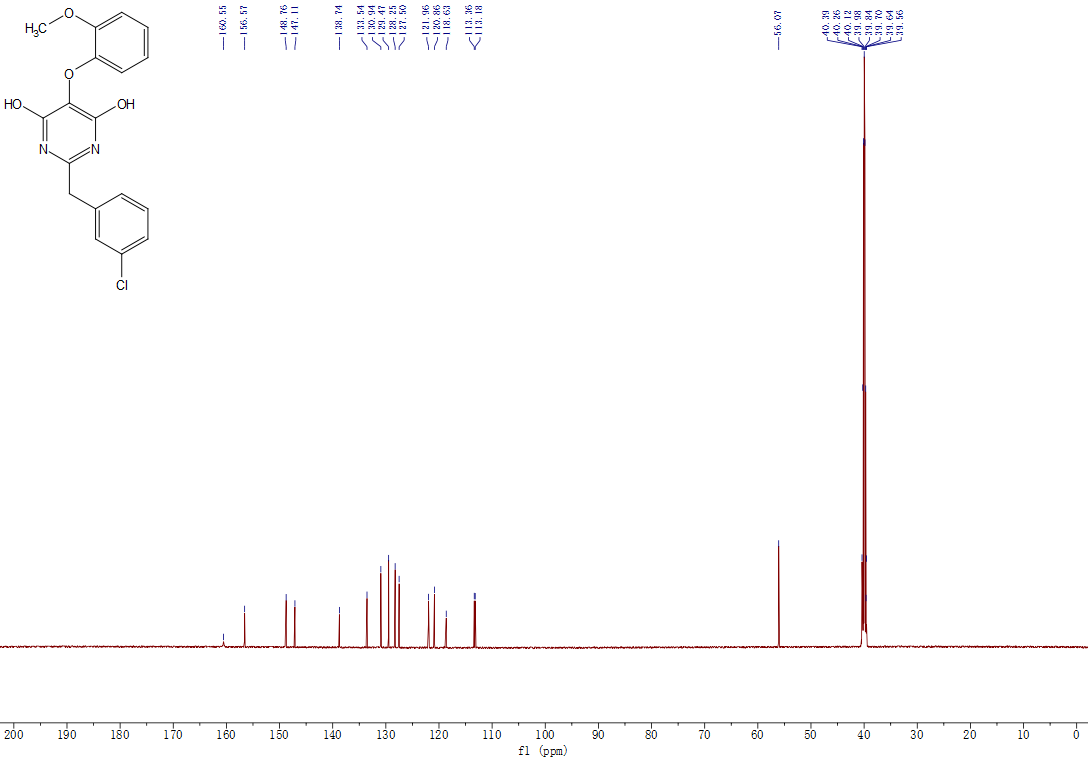


**Figure S20.** **^13^C-NMR spectra of 6g**


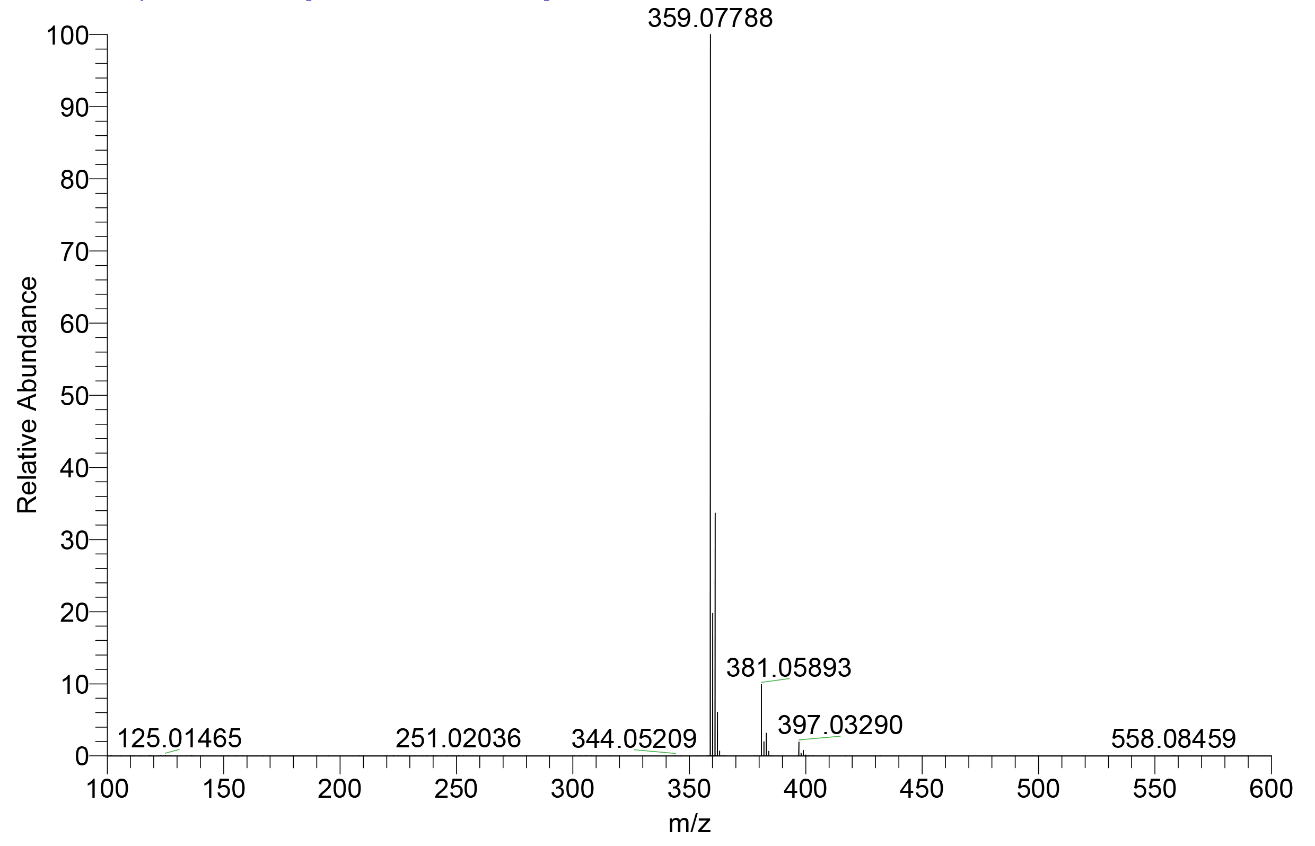


**Figure S21. Mass spectrum of 6g**

*
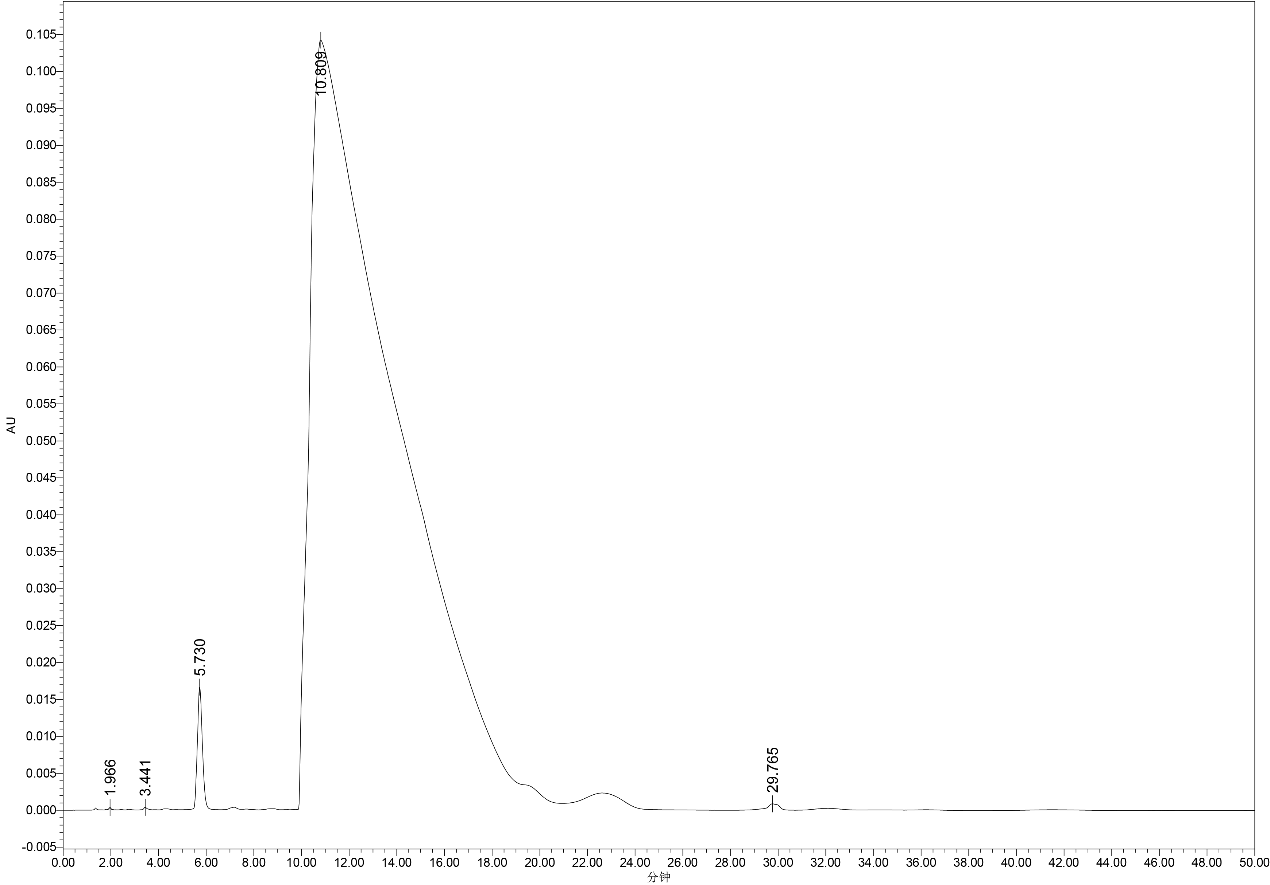
*

**Table S7.** HPLC analysis of **6g** (1.0mL/min, MeOH: Water = 60:40 v/v).

| Peak | Retention time | Peak area | Peak area % | Peak start | Peak end |
| --- | --- | --- | --- | --- | --- |
| 1 | 1.966 | 1657 | 0.01 | 1.867 | 2.117 |
| 2 | 3.441 | 3181 | 0.01 | 3.3 | 4.033 |
| 3 | 5.73 | 240528 | 0.96 | 4.95 | 6.183 |
| 4 | 10.809 | 24740778 | 98.96 | 9.867 | 18.95 |
| 5 | 29.765 | 15114 | 0.06 | 29.533 | 30.183 |

*2.8 2-(4-chlorobenzyl)-5-(2-methoxyphenoxy)pyrimidine-4,6-diol (****6h****)*


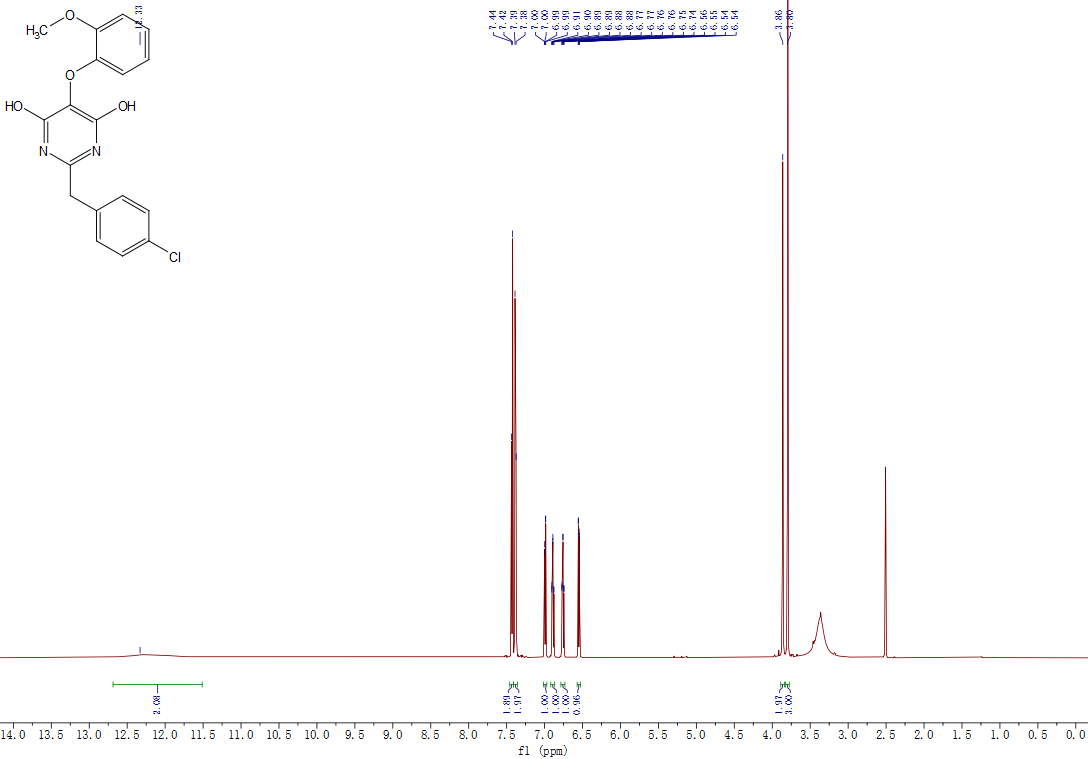


**Figure S22.** **^1^H-NMR spectra of 6h**


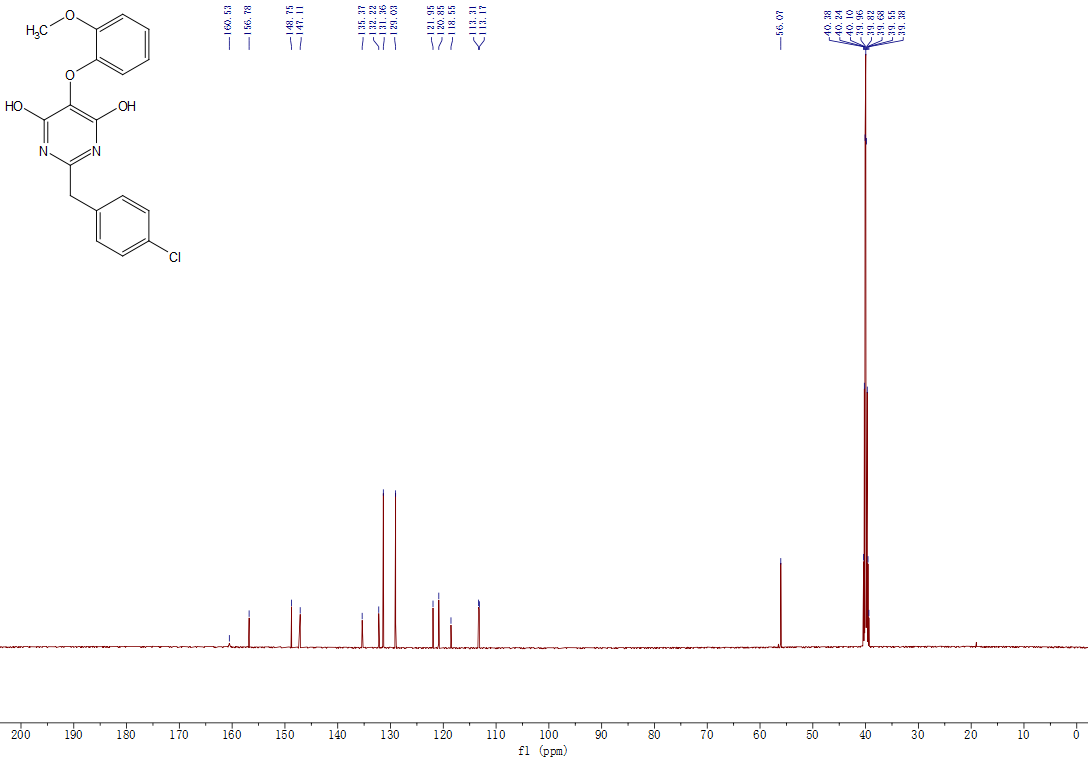


**Figure S23. ^13^C-NMR spectra of 6h**


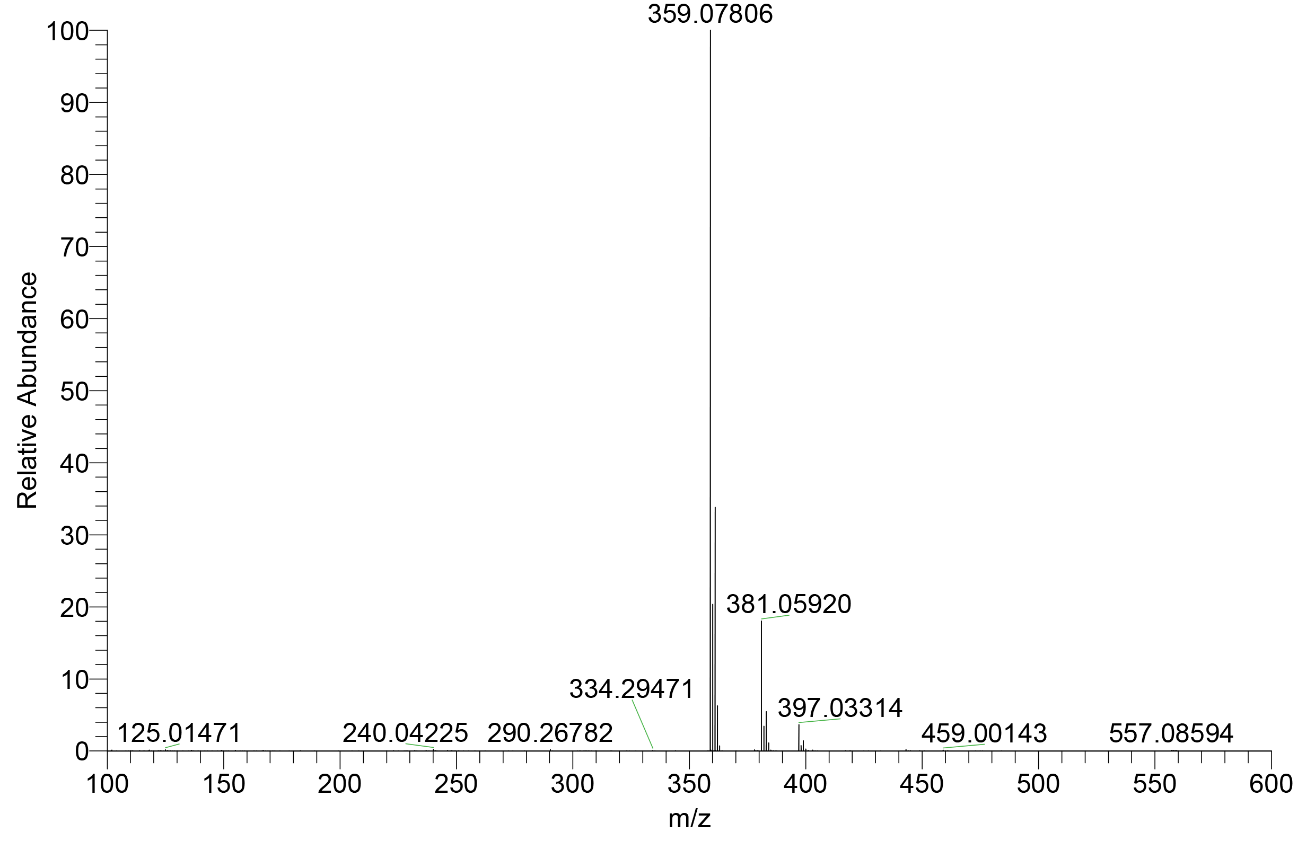


**Figure S24 Mass spectrum of 6h.**

*
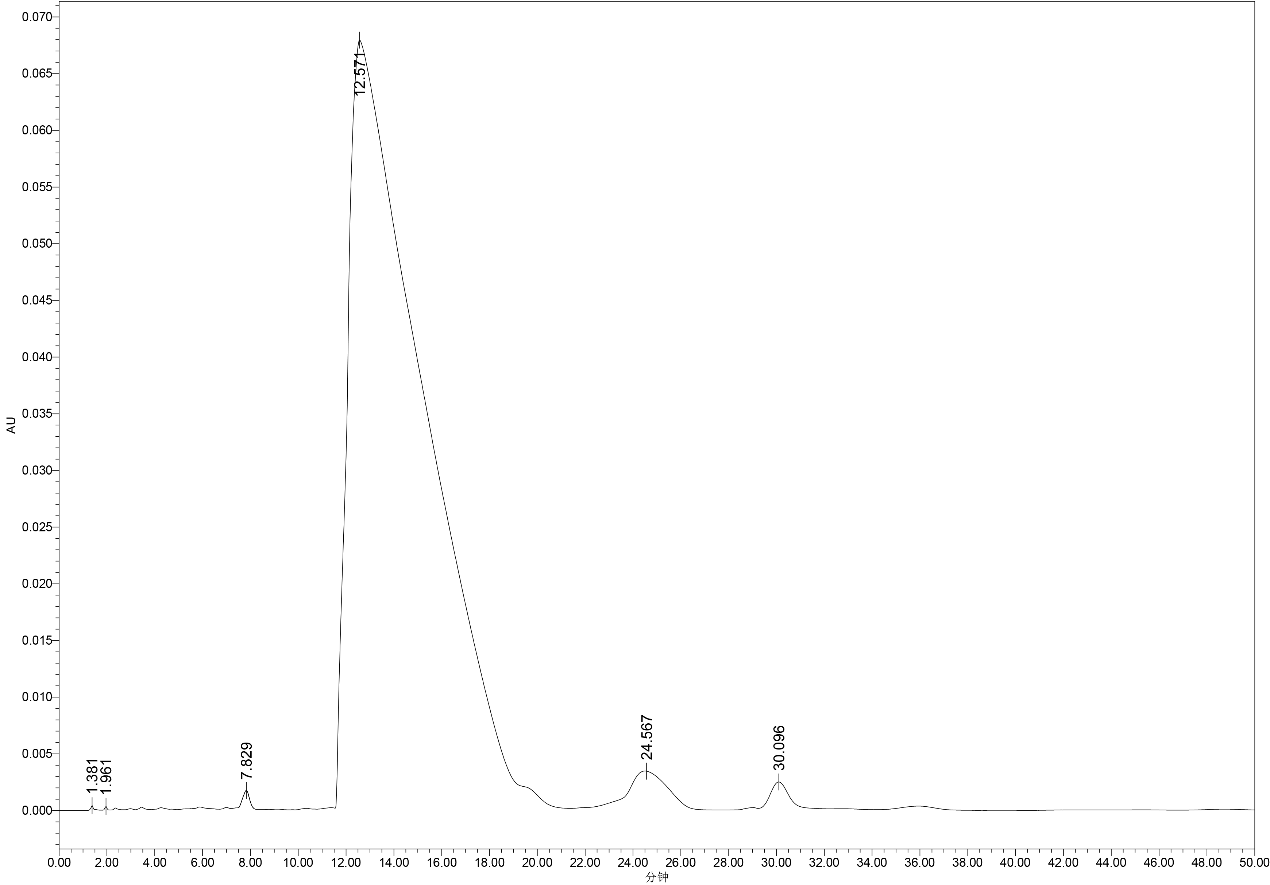
*

**Table S8.** HPLC analysis of **6h** (1.0mL/min, MeOH: Water = 60:40 v/v).

| Peak | Retention time | Peak area | Peak area % | Peak start | Peak end |
| --- | --- | --- | --- | --- | --- |
| 1 | 1.381 | 3286 | 0.02 | 1.233 | 1.8 |
| 2 | 1.961 | 1983 | 0.01 | 1.8 | 2.117 |
| 3 | 7.829 | 30573 | 0.2 | 7.467 | 8.183 |
| 4 | 12.571 | 14819021 | 97.68 | 11.55 | 20.683 |
| 5 | 2267 | 228742 | 1.51 | 23.7 | 26.533 |
| 6 | 30.096 | 87415 | 0.58 | 29.283 | 30.717 |

*2.9 2-(3-bromobenzyl)-5-(2-methoxyphenoxy)pyrimidine-4,6-diol (****6i****)*


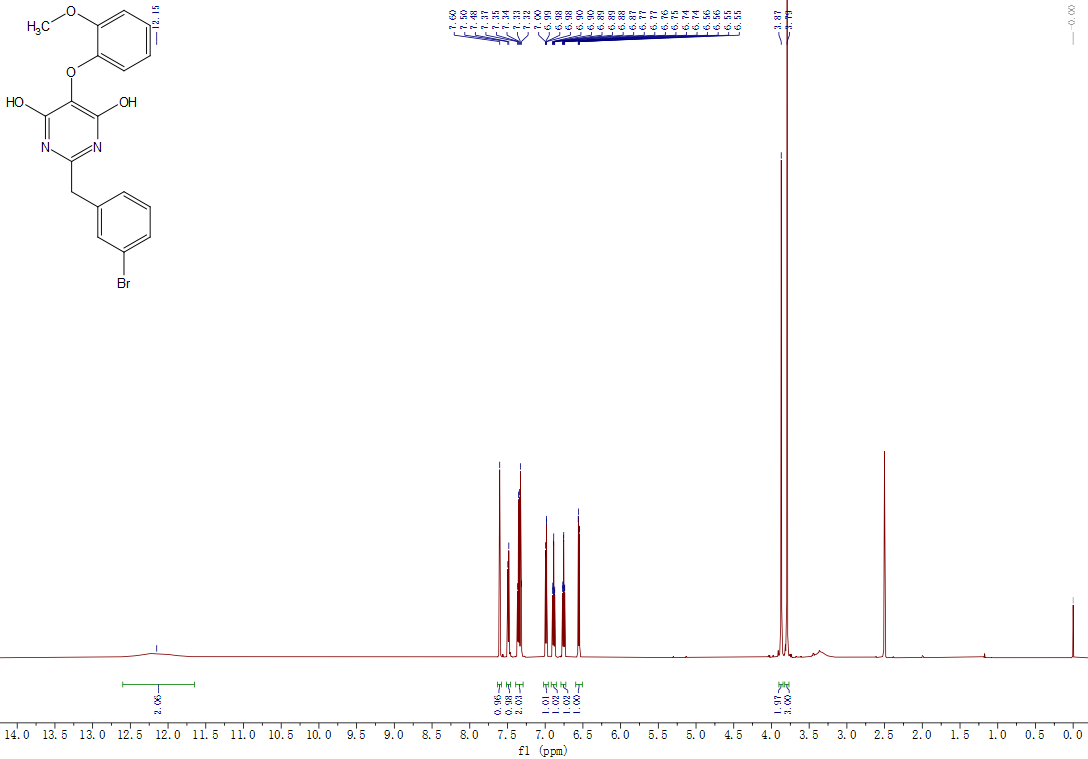


**Figure S25. ^1^H-NMR spectra of 6i**


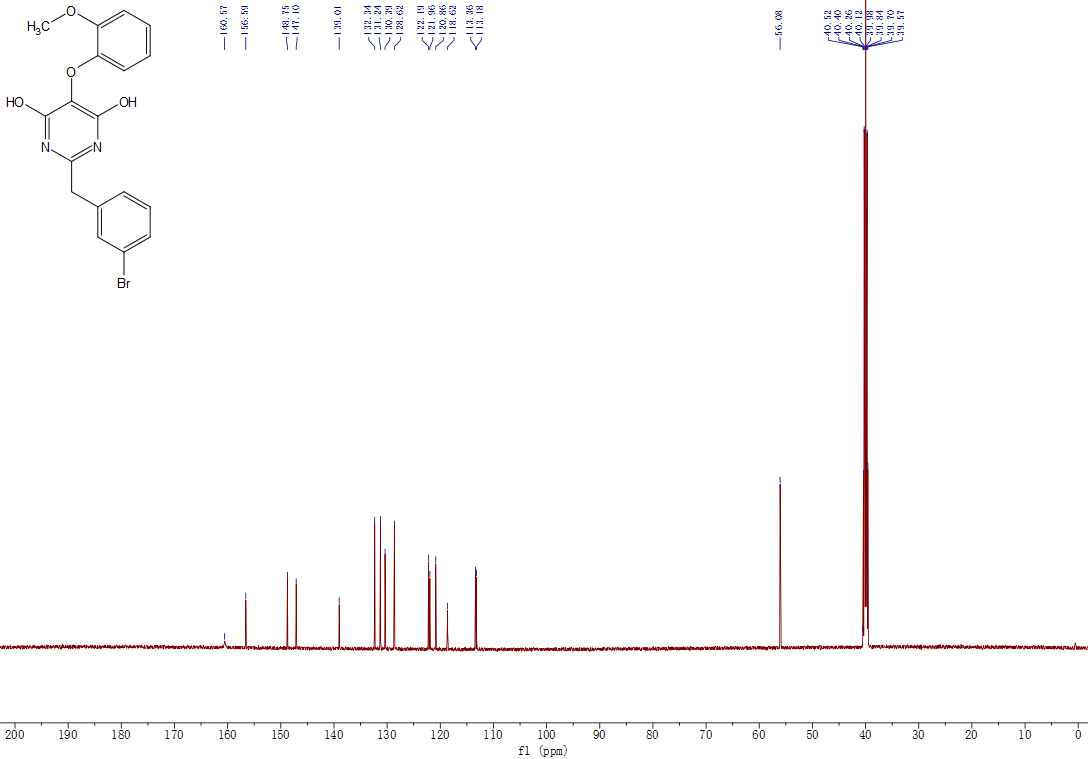


**Figure S26. ^13^C-NMR spectra of 6i**


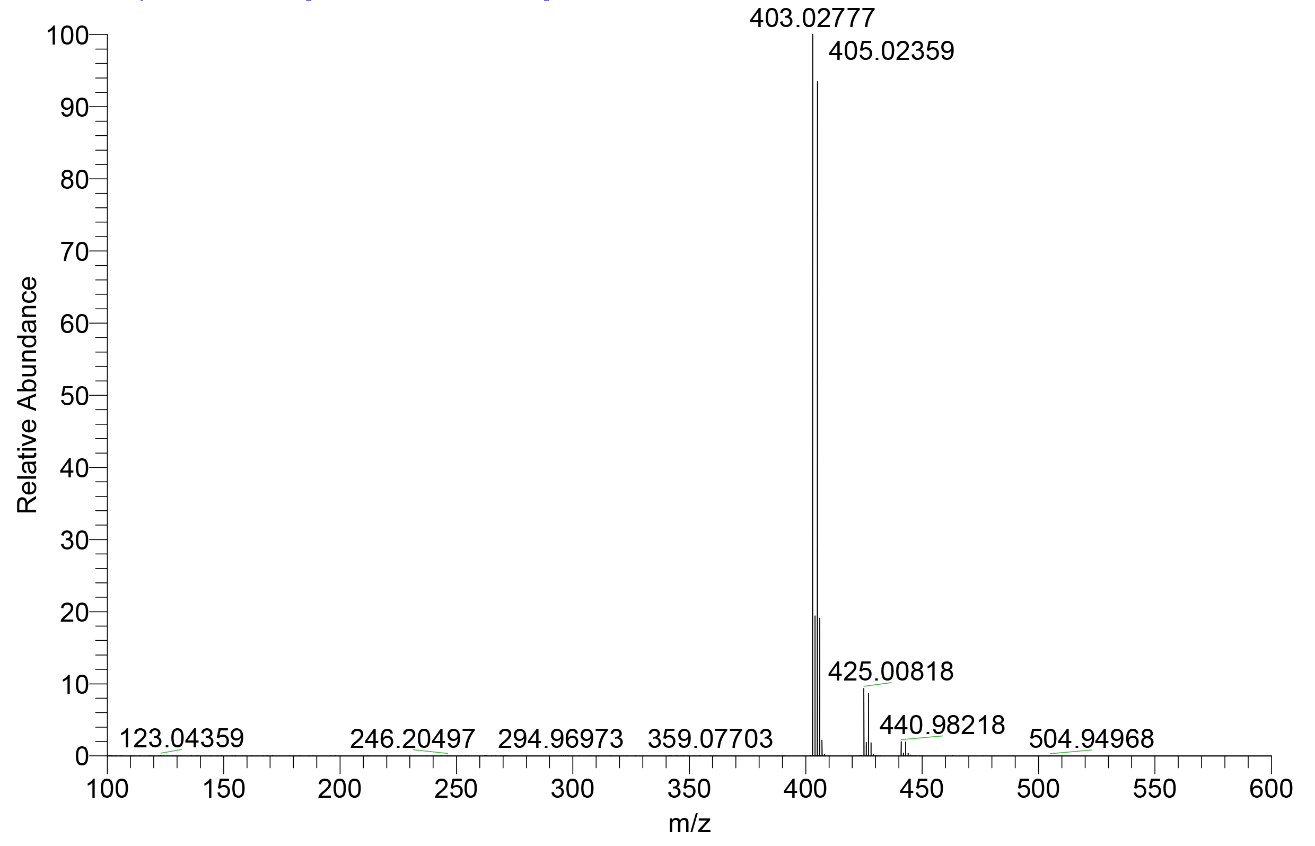


**Figure S27. Mass spectrum of 6i**

*
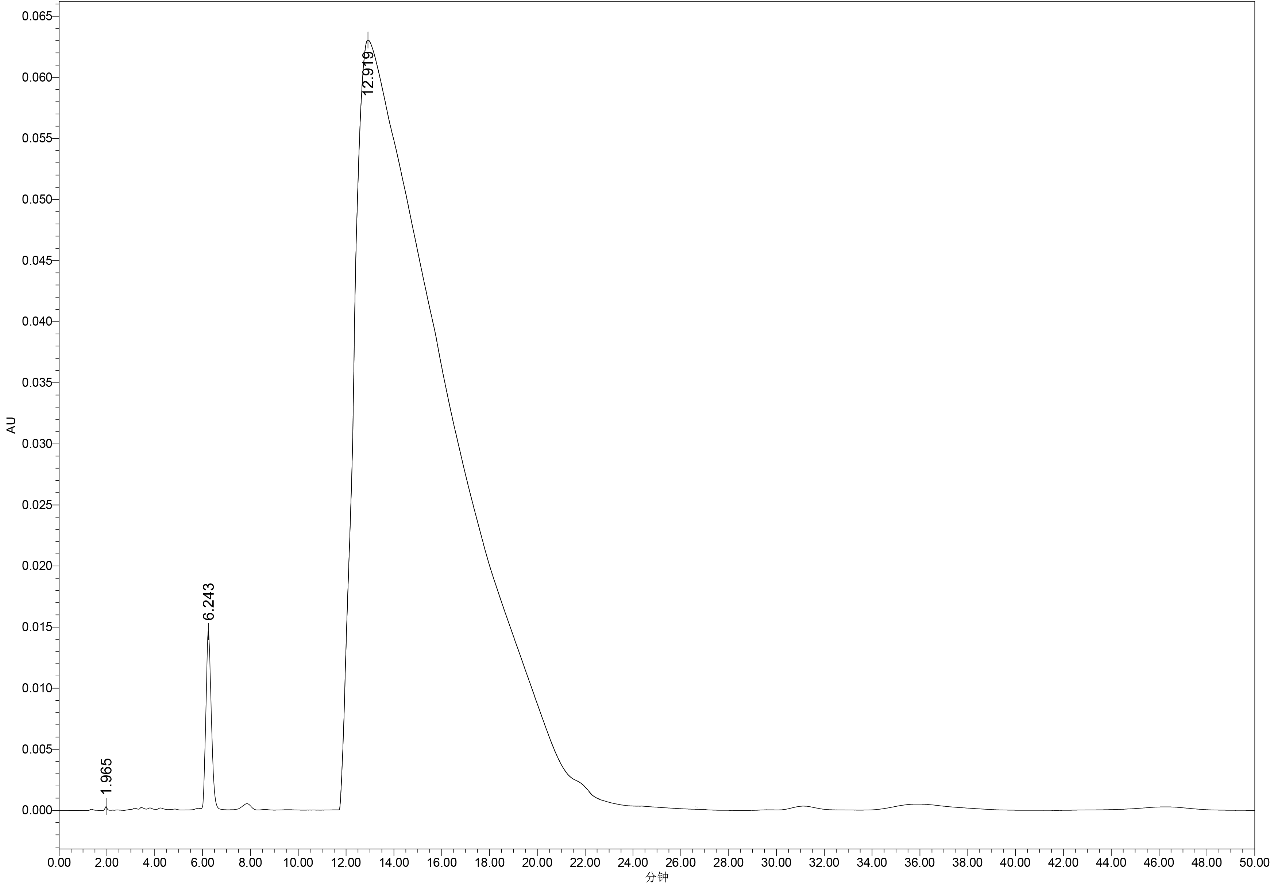
*

**Table S9.** HPLC analysis of **6i** (1.0mL/min, MeOH: Water = 60:40 v/v).

| Peak | Retention time | Peak area | Peak area % | Peak start | Peak end |
| --- | --- | --- | --- | --- | --- |
| 1 | 1.965 | 1852 | 0.01 | 1.833 | 2.117 |
| 2 | 6.243 | 225507 | 1.29 | 5.917 | 6.717 |
| 3 | 12.919 | 17252638 | 98.7 | 11.7 | 22.667 |

*2.10 2-(4-bromobenzyl)-5-(2-methoxyphenoxy)pyrimidine-4,6-diol (****6j****)*


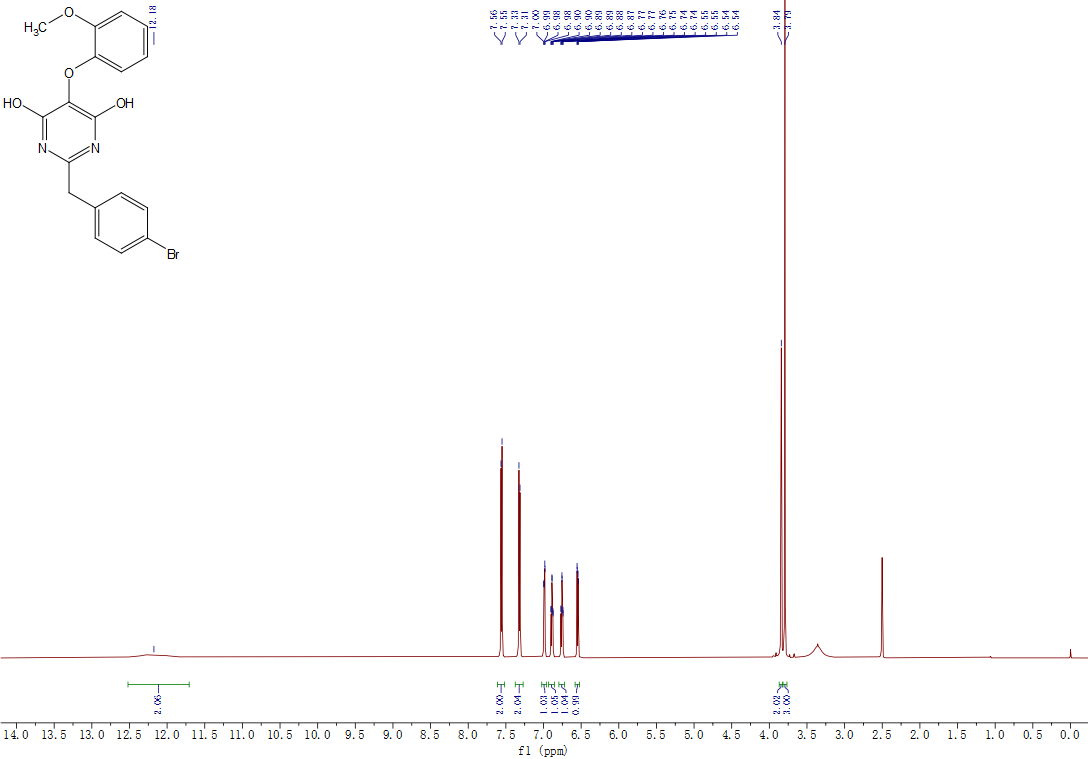


**Figure S28. ^1^H-NMR spectra of 6j**


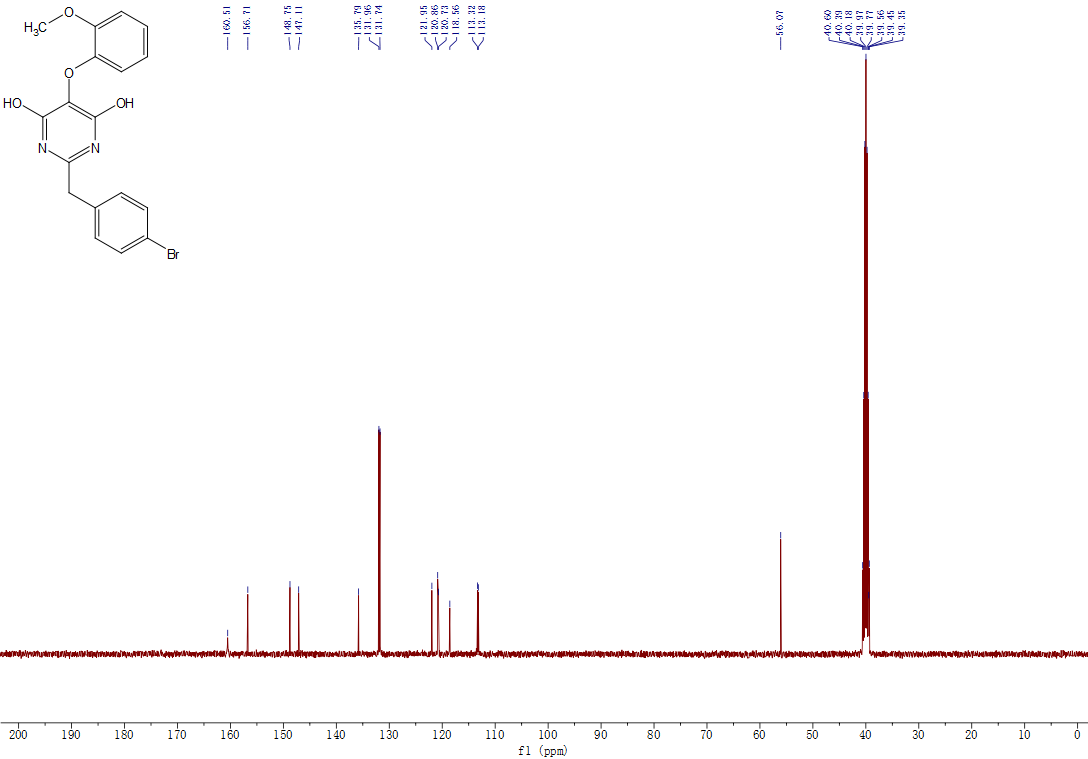


**Figure S29. ^13^C-NMR spectra of 6j**


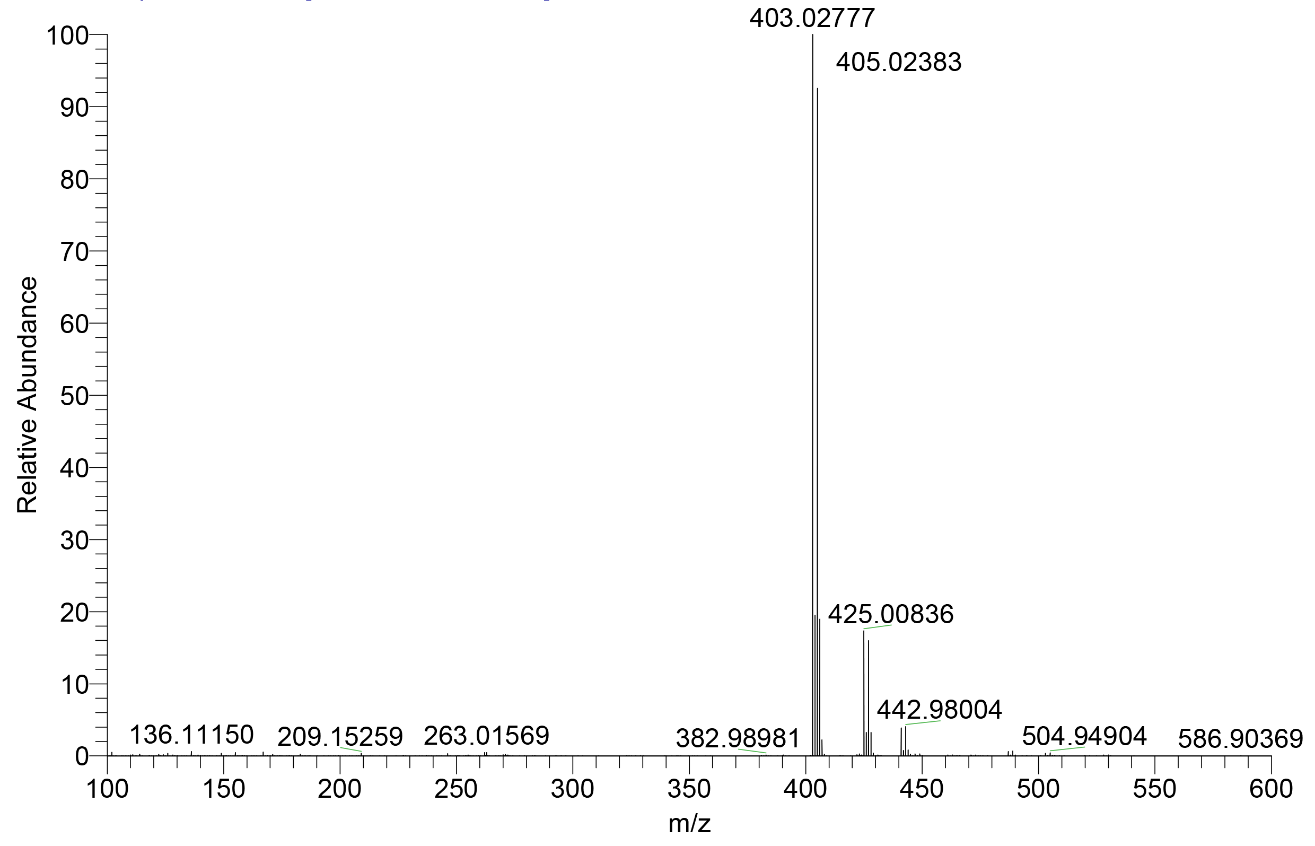


**Figure S30. Mass spectrum of 6j**

*
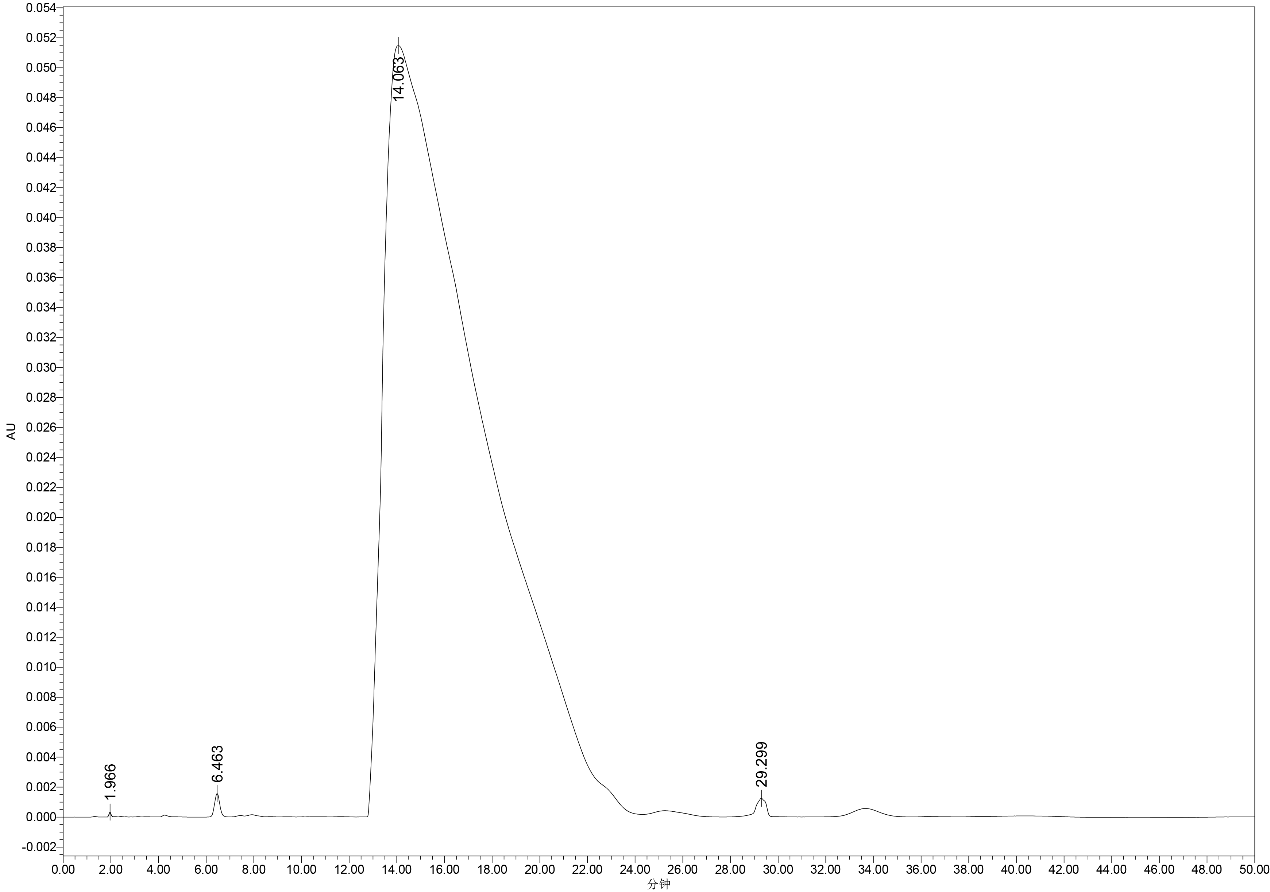
*

**Table S10.** HPLC analysis of **6j** (1.0mL/min, MeOH: Water = 60:40 v/v).

| Peak | Retention time | Peak area | Peak area % | Peak start | Peak end |
| --- | --- | --- | --- | --- | --- |
| 1 | 1.966 | 1847 | 0.01 | 1.85 | 2.117 |
| 2 | 6.463 | 21982 | 0.15 | 6.2 | 6.75 |
| 3 | 14.063 | 14511127 | 99.64 | 12.767 | 23.933 |
| 4 | 29.299 | 28160 | 0.19 | 28.933 | 29.683 |

*2.11 4,6-dichloro-2-(3-chlorophenyl)-5-(2-methoxyphenoxy)pyrimidine (****7a****)*


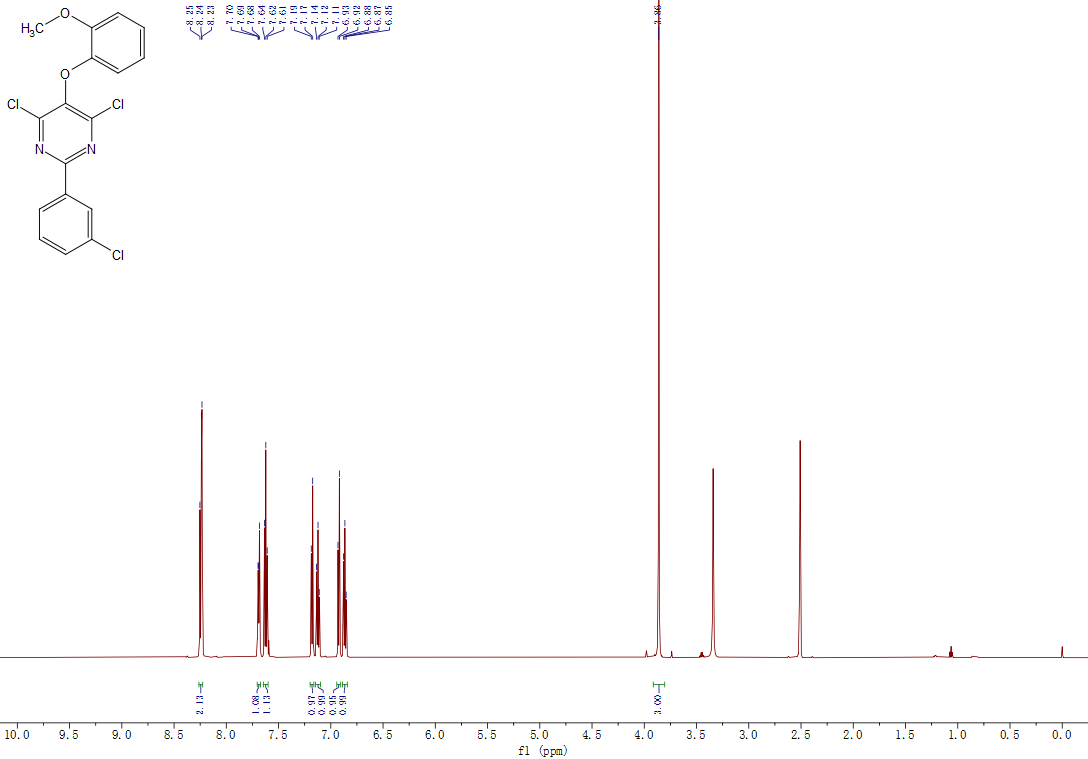


**Figure S31. ^1^H-NMR spectra of 7a**


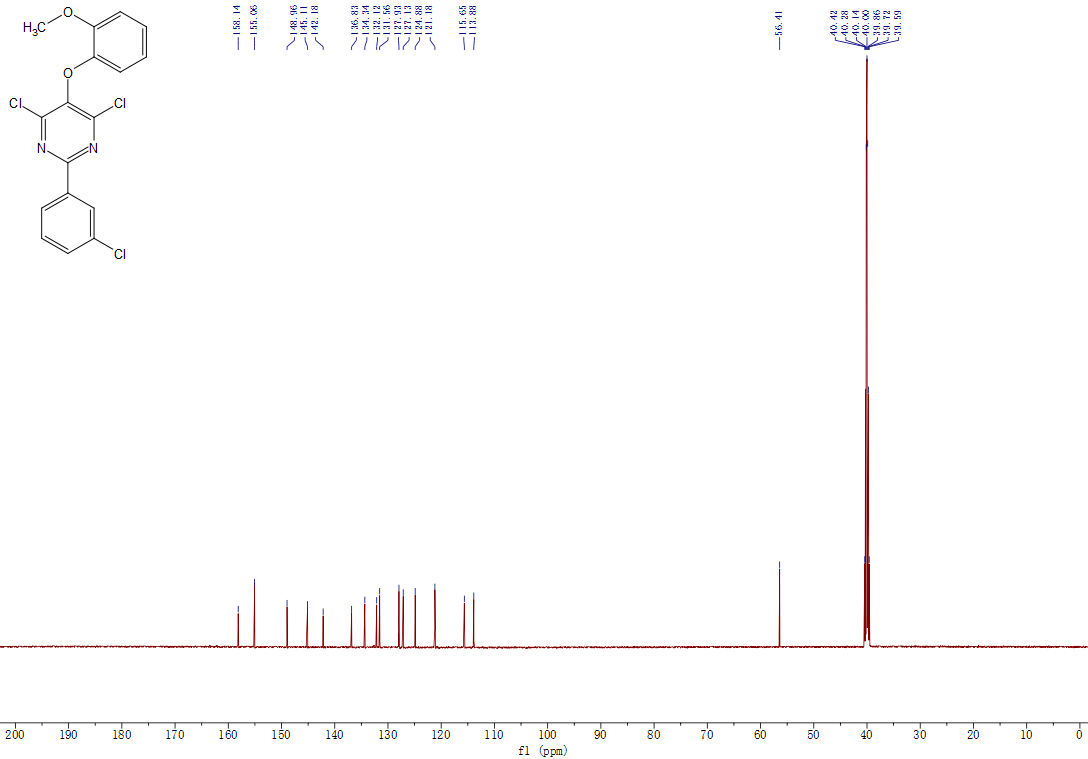


**Figure S32. ^13^C-NMR spectra of 7a**


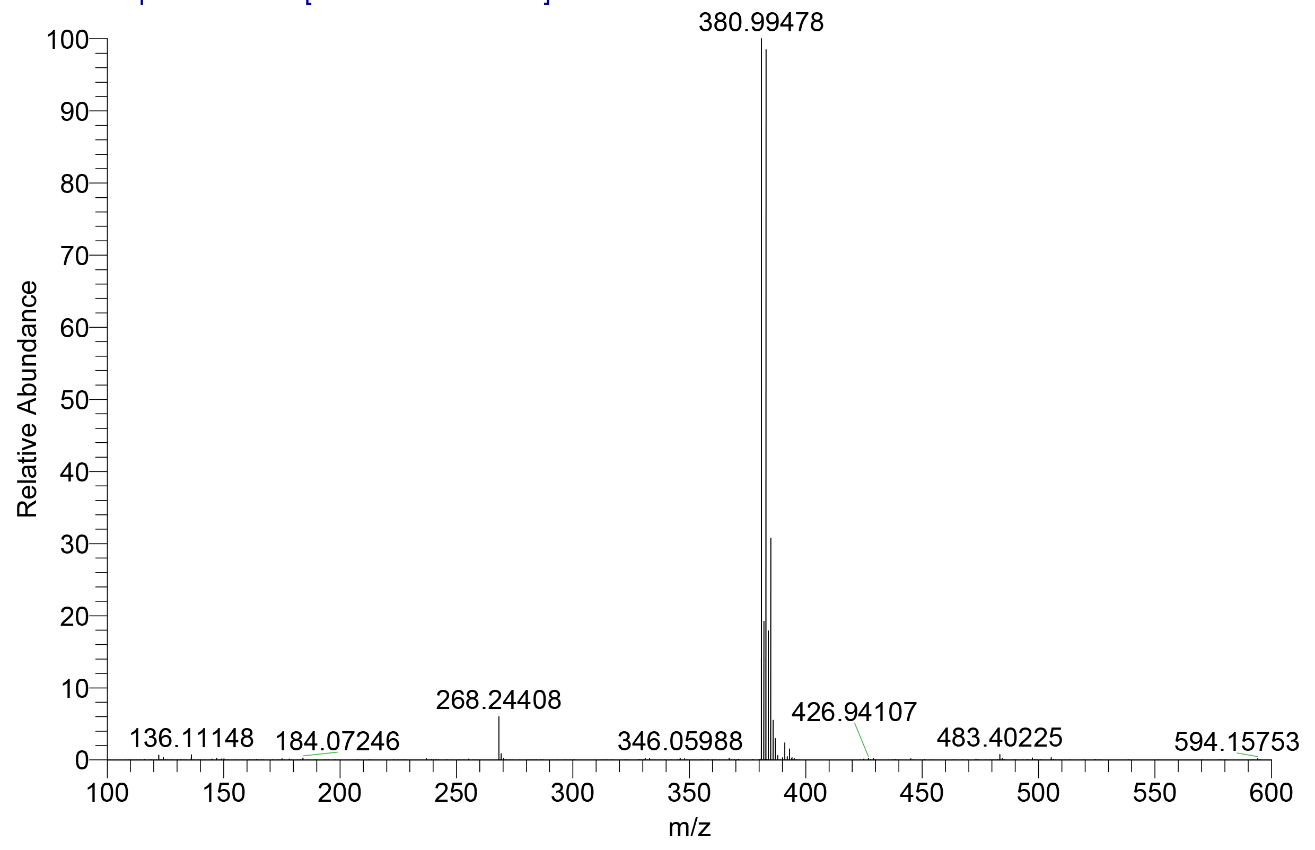


**Figure S33. Mass spectrum of 7a**

*
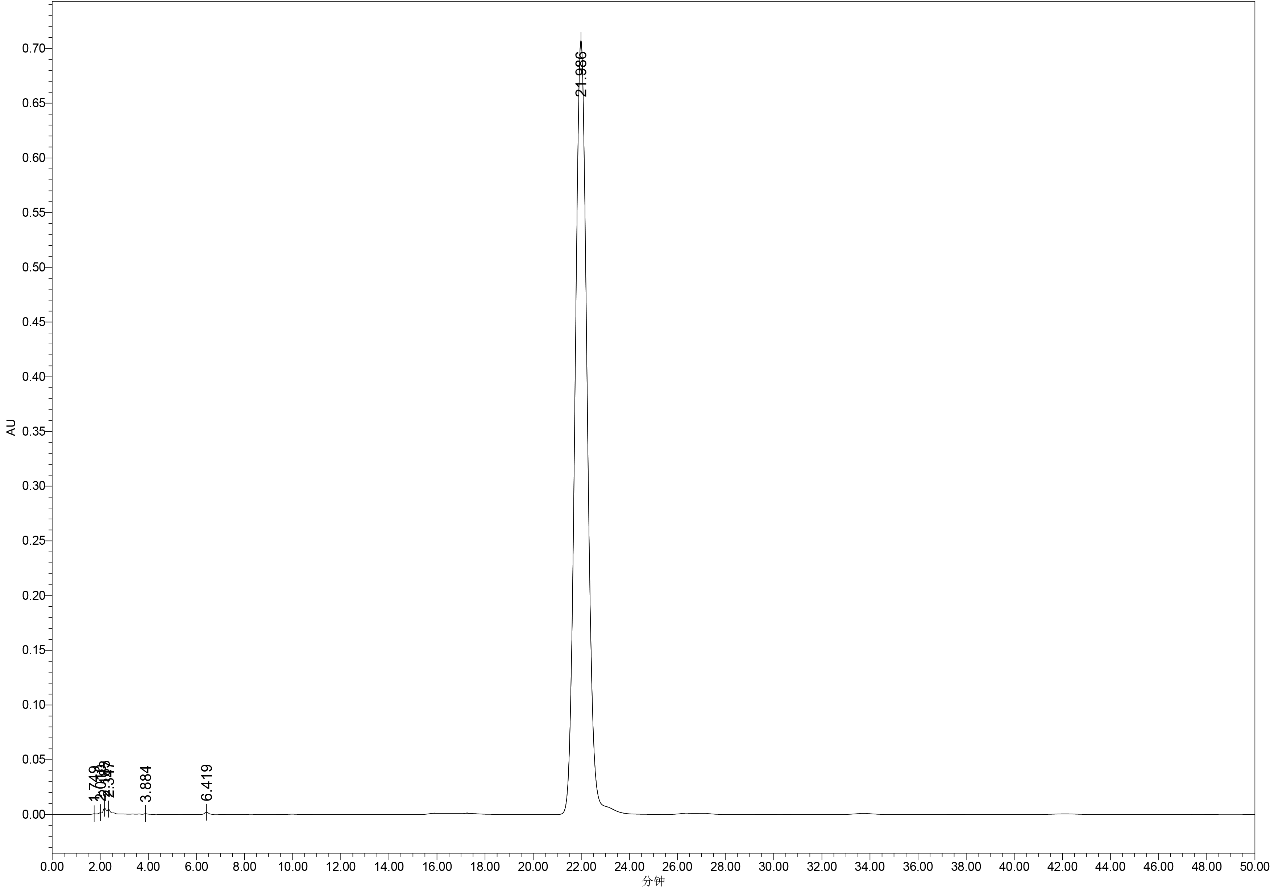
*

**Table S11.** HPLC analysis of **7a** (1.0mL/min, Acetonitrile: Water = 55:45 v/v).

| Peak | Retention time | Peak area | Peak area % | Peak start | Peak end |
| --- | --- | --- | --- | --- | --- |
| 1 | 1.749 | 4660 | 0.02 | 1.6 | 1.85 |
| 2 | 2.01 | 10604 | 0.04 | 1.85 | 2.05 |
| 3 | 2.183 | 49579 | 0.2 | 2.05 | 2.283 |
| 4 | 2.347 | 46737 | 0.19 | 2.283 | 2.783 |
| 5 | 3.884 | 5261 | 0.02 | 3.75 | 4.083 |
| 6 | 6.419 | 22708 | 0.09 | 6.167 | 6.683 |
| 7 | 21.986 | 24731666 | 99.44 | 21.067 | 23.883 |

*2.12 4,6-dichloro-2-(4-chlorophenyl)-5-(2-methoxyphenoxy)pyrimidine (****7b****)*


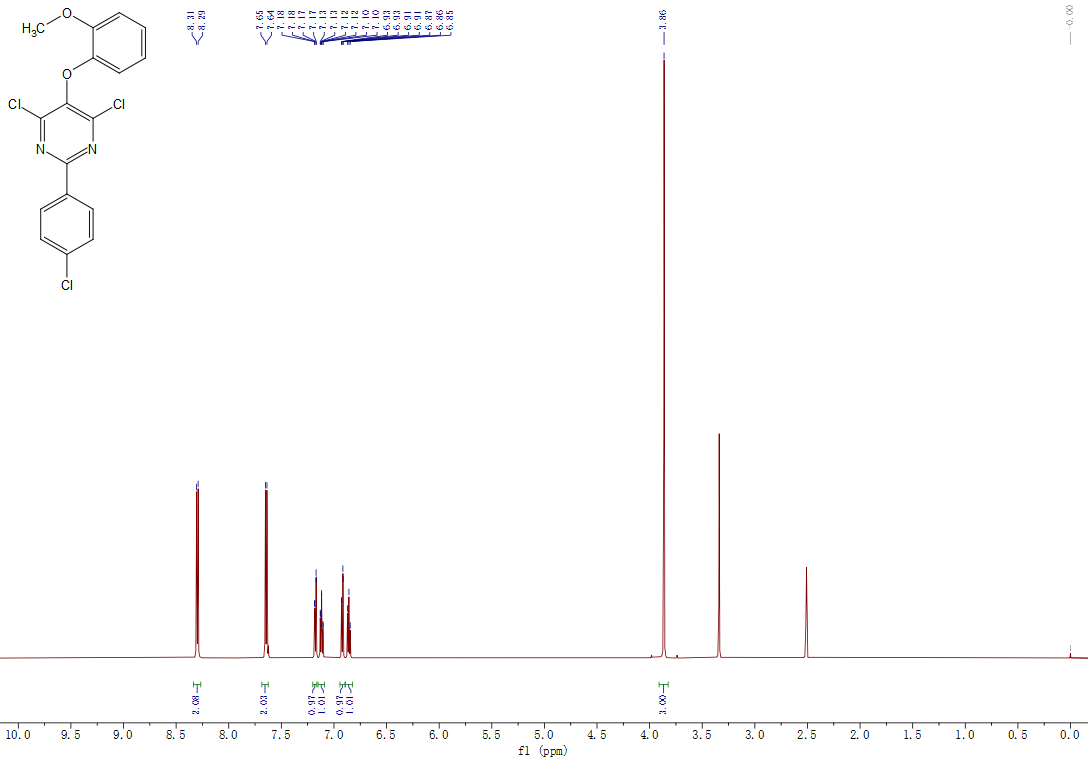


**Figure S34. ^1^H-NMR spectra of 7b**


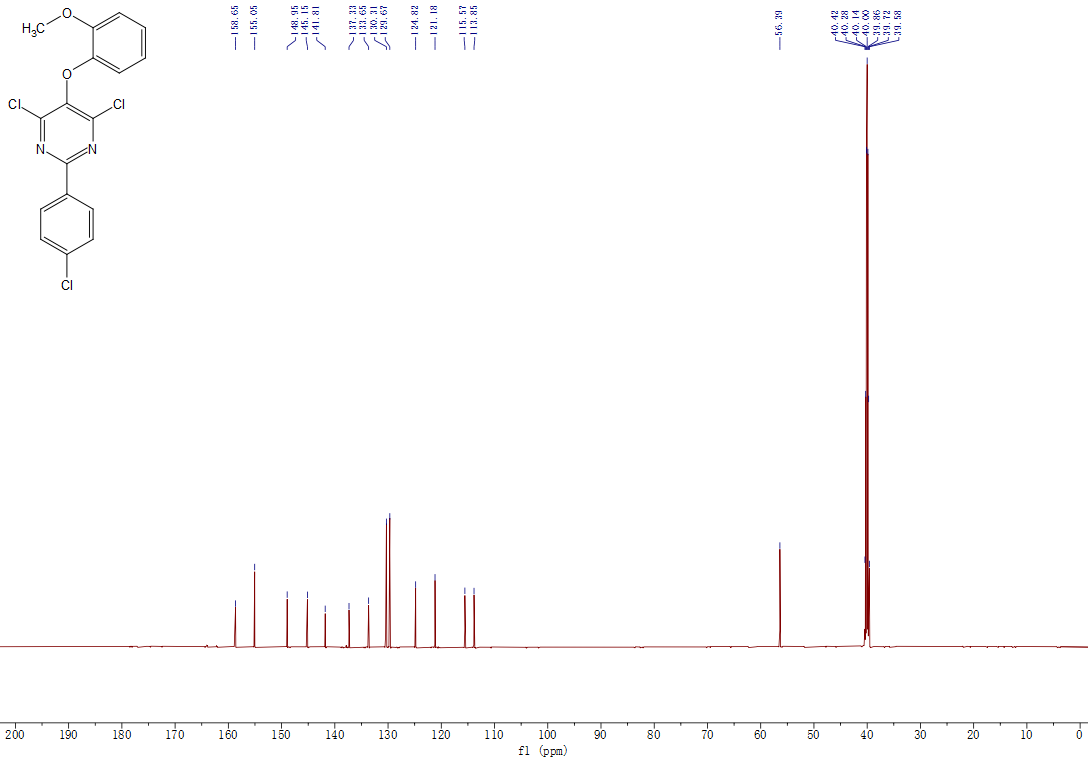


**Figure S35. ^13^C-NMR spectra of 7b**


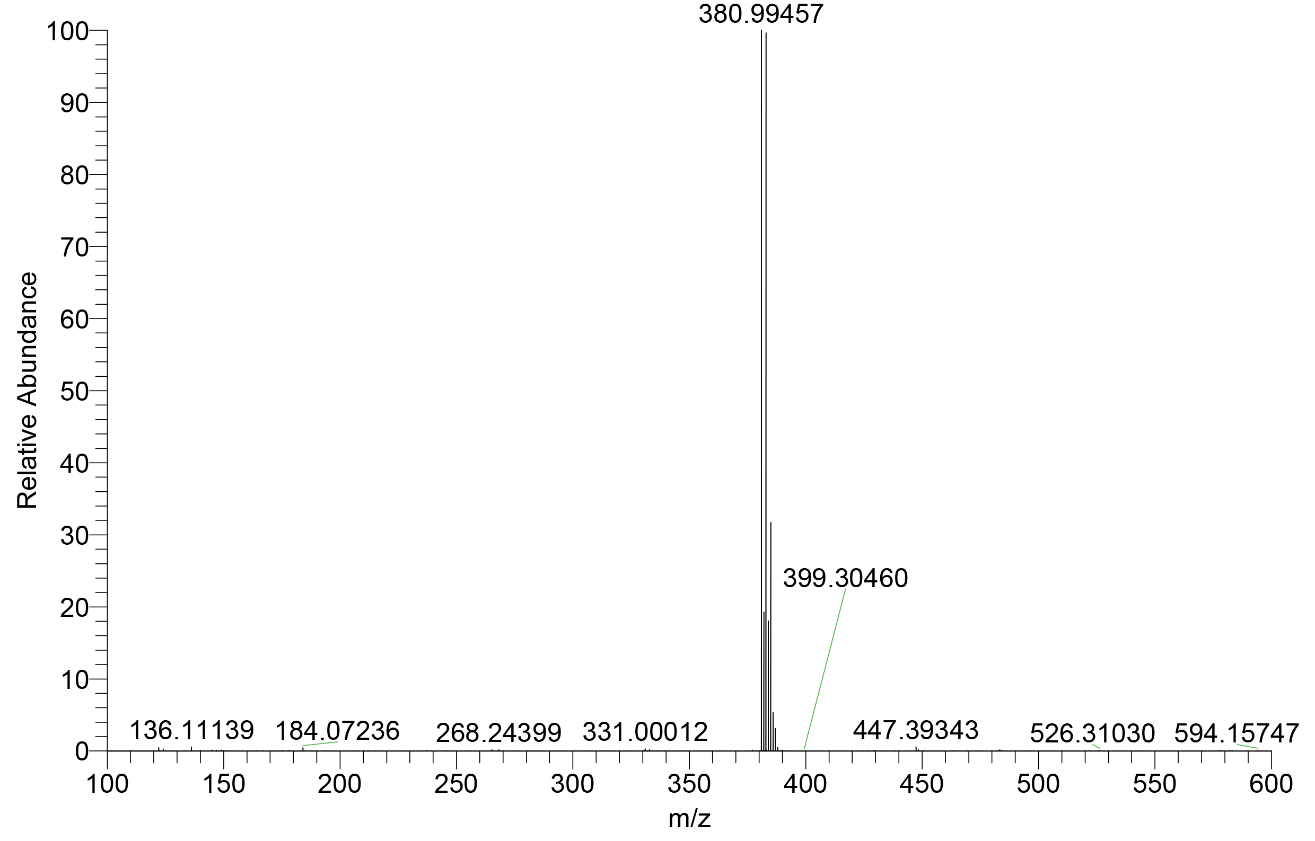


**Figure S36. Mass spectrum of 7b**

*
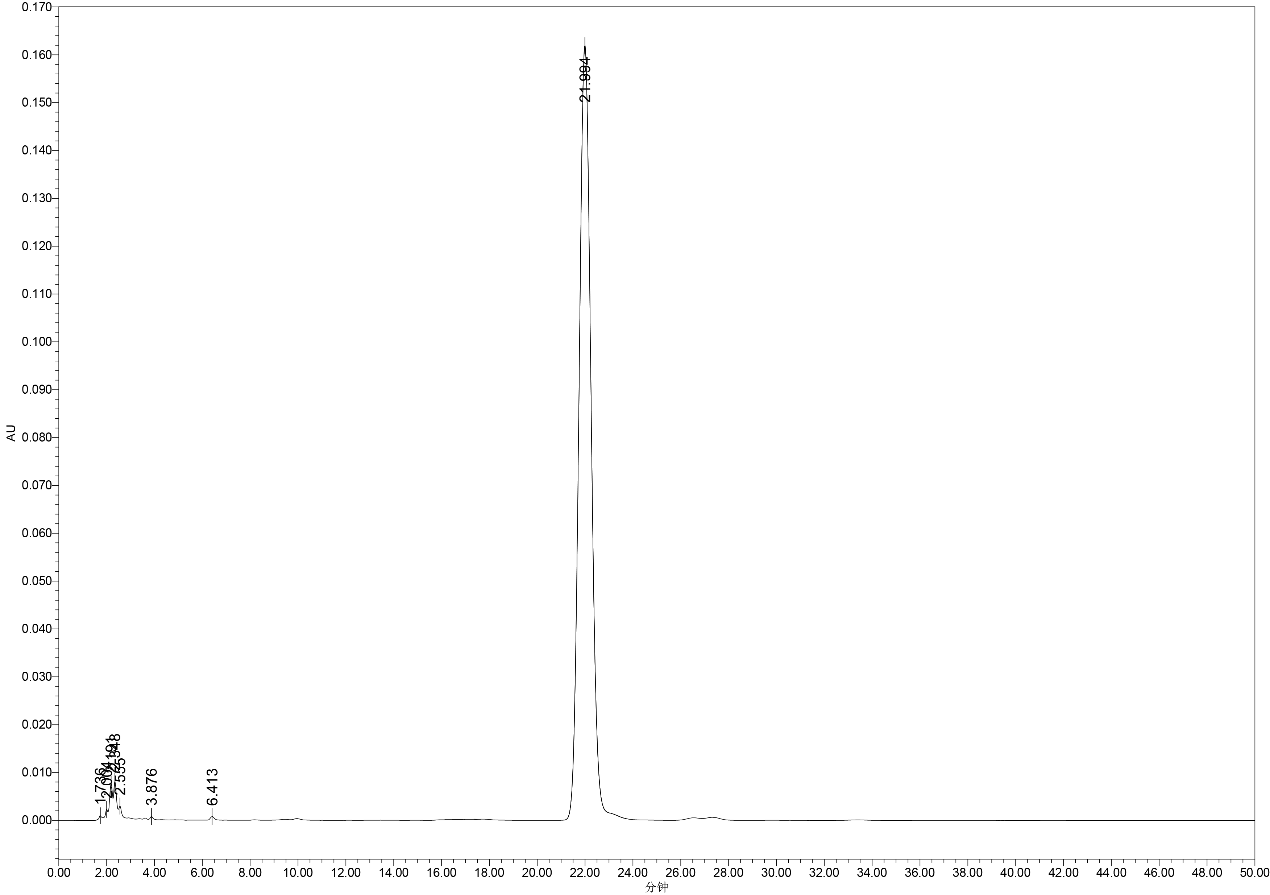
*

**Table S12.** HPLC analysis of **7b** (1.0mL/min, Acetonitrile: Water = 55:45 v/v).

| Peak | Retention time | Peak area | Peak area % | Peak start | Peak end |
| --- | --- | --- | --- | --- | --- |
| 1 | 1.736 | 7225 | 0.12 | 1.567 | 1.85 |
| 2 | 2.004 | 15597 | 0.27 | 1.85 | 2.067 |
| 3 | 2.191 | 61006 | 1.05 | 2.067 | 2.267 |
| 4 | 2.348 | 66412 | 1.14 | 2.267 | 2.483 |
| 5 | 2.555 | 21817 | 0.38 | 2.483 | 2.817 |
| 6 | 3.876 | 4682 | 0.08 | 3.75 | 4.05 |
| 7 | 6.413 | 7771 | 0.13 | 6.2 | 6.617 |
| 8 | 21.994 | 5629658 | 96.83 | 21.167 | 23.733 |

*2.13 2-(3-bromophenyl)-4,6-dichloro-5-(2-methoxyphenoxy)pyrimidine (****7c****)*


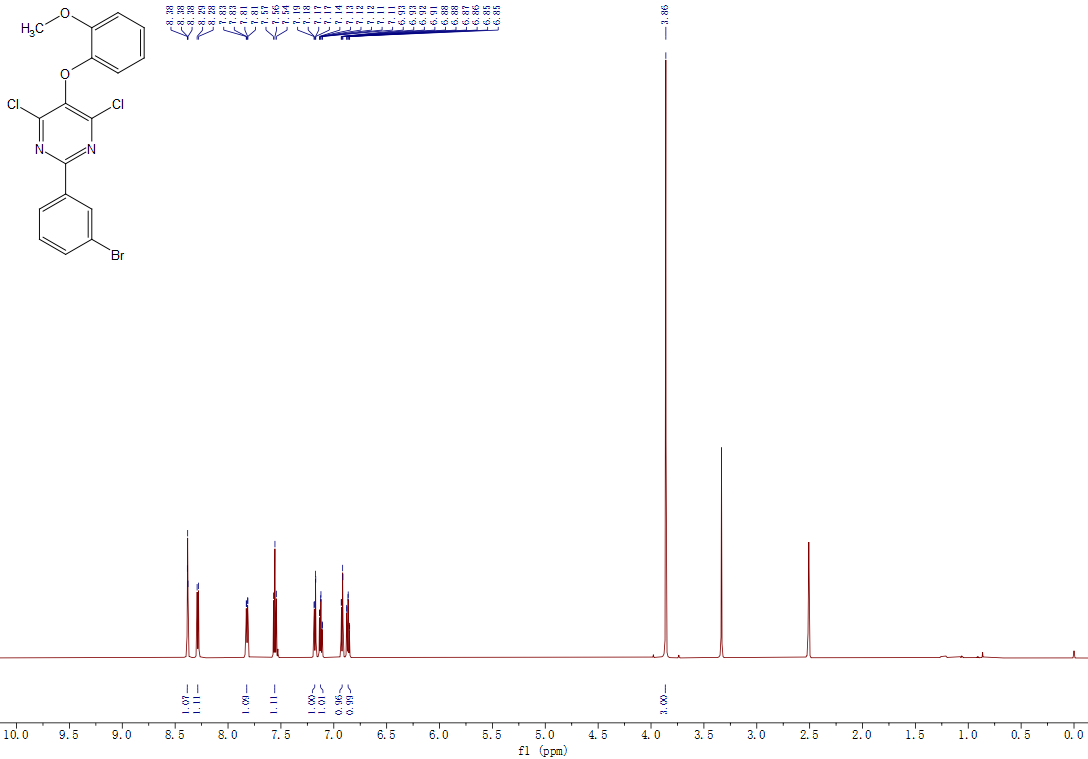


**Figure S37. ^1^H-NMR spectra of 7c**


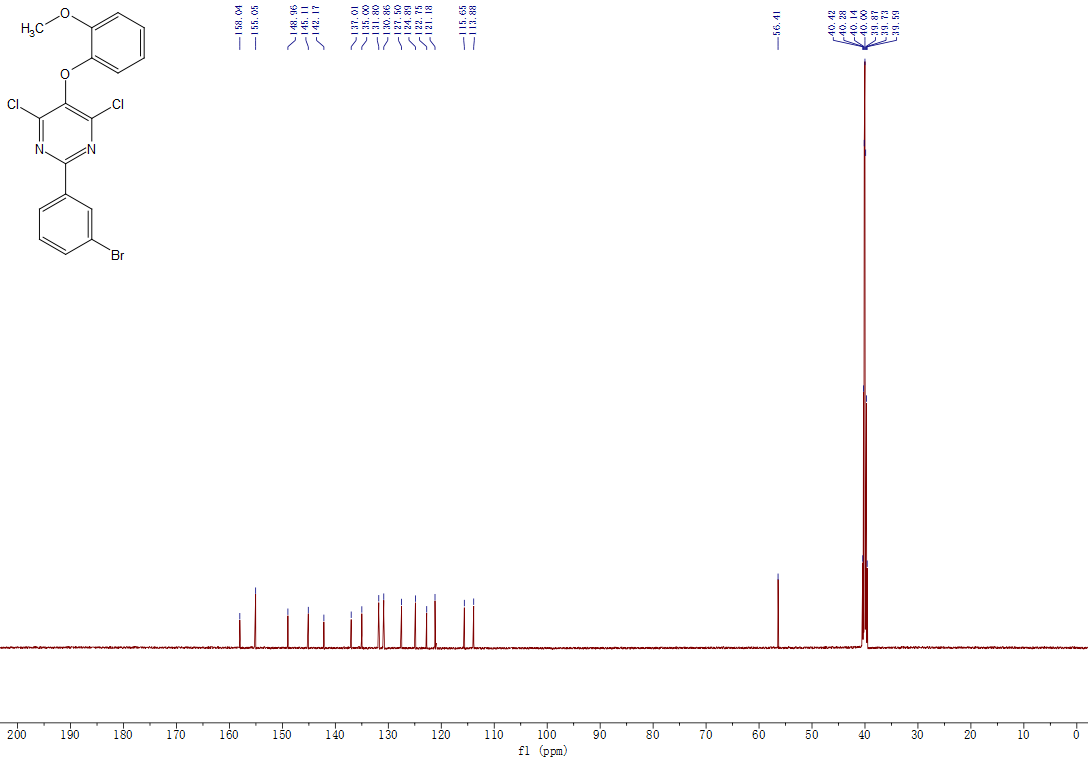


**Figure S38. ^13^C-NMR spectra of 7c**


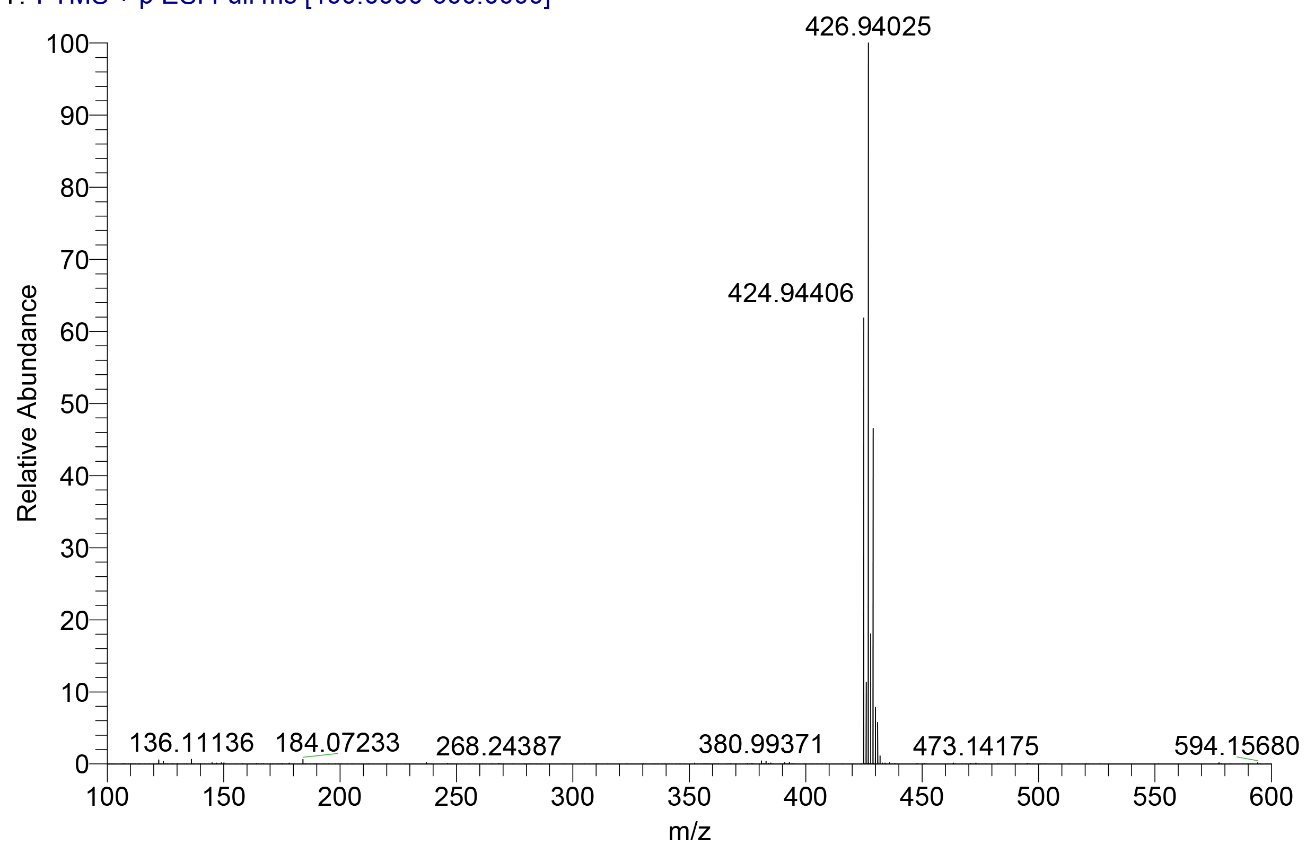


**Figure S39. Mass spectrum of 7c**

*
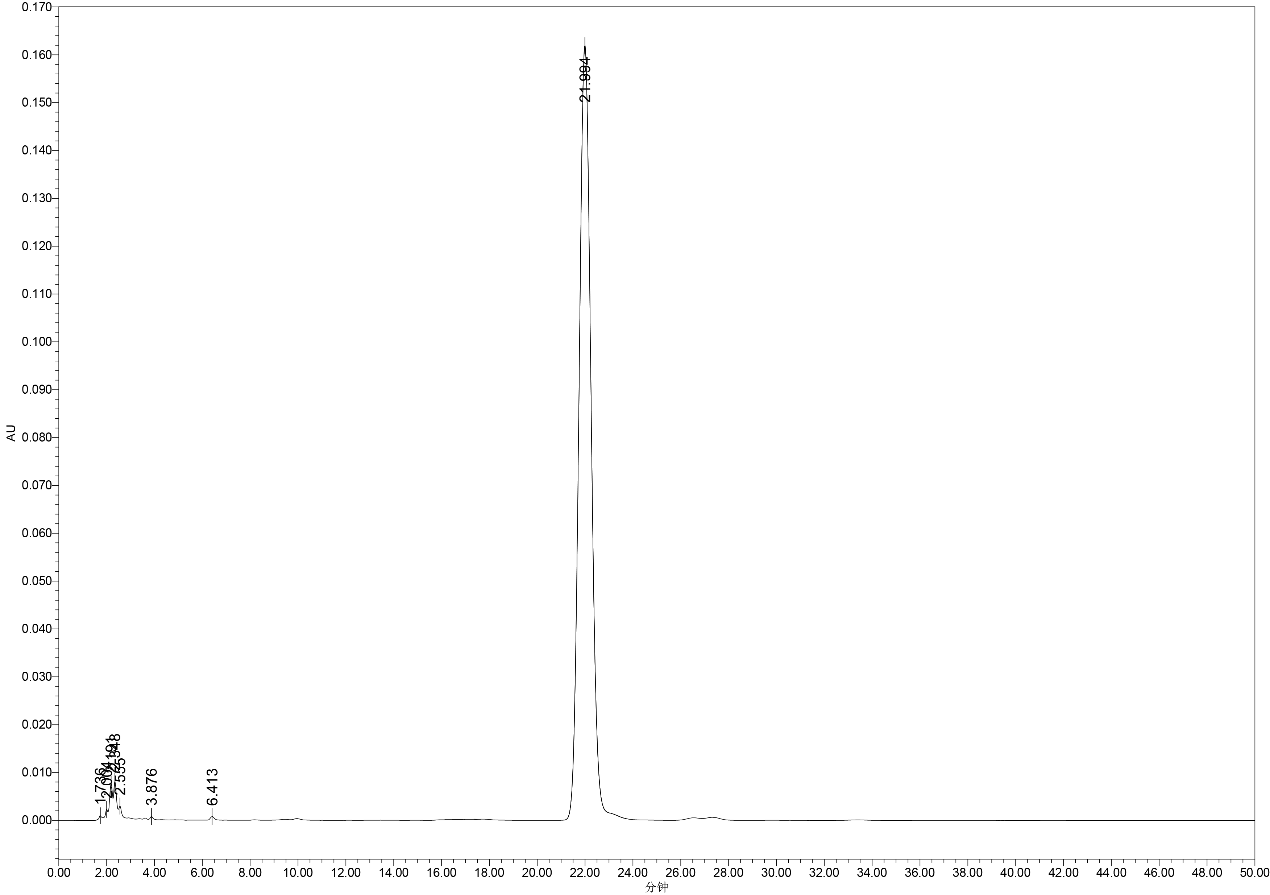
*

**Table S13.** HPLC analysis of **7c** (1.0mL/min, Acetonitrile: Water = 55:45 v/v).

| Peak | Retention time | Peak area | Peak area % | Peak start | Peak end |
| --- | --- | --- | --- | --- | --- |
| 1 | 1.74 | 7500 | 0.15 | 1.6 | 1.883 |
| 2 | 2.007 | 6990 | 0.14 | 1.883 | 2.05 |
| 3 | 2.177 | 36167 | 0.75 | 2.05 | 2.267 |
| 4 | 2.341 | 37306 | 0.77 | 2.267 | 2.5 |
| 5 | 2.563 | 10986 | 0.23 | 2.5 | 2.783 |
| 6 | 3.867 | 5736 | 0.12 | 3.717 | 4.05 |
| 7 | 6.414 | 8166 | 0.17 | 6.2 | 6.65 |
| 8 | 24.269 | 4730798 | 97.67 | 23.367 | 25.317 |

*2.14 2-(4-bromophenyl)-4,6-dichloro-5-(2-methoxyphenoxy)pyrimidine (****7d****)*


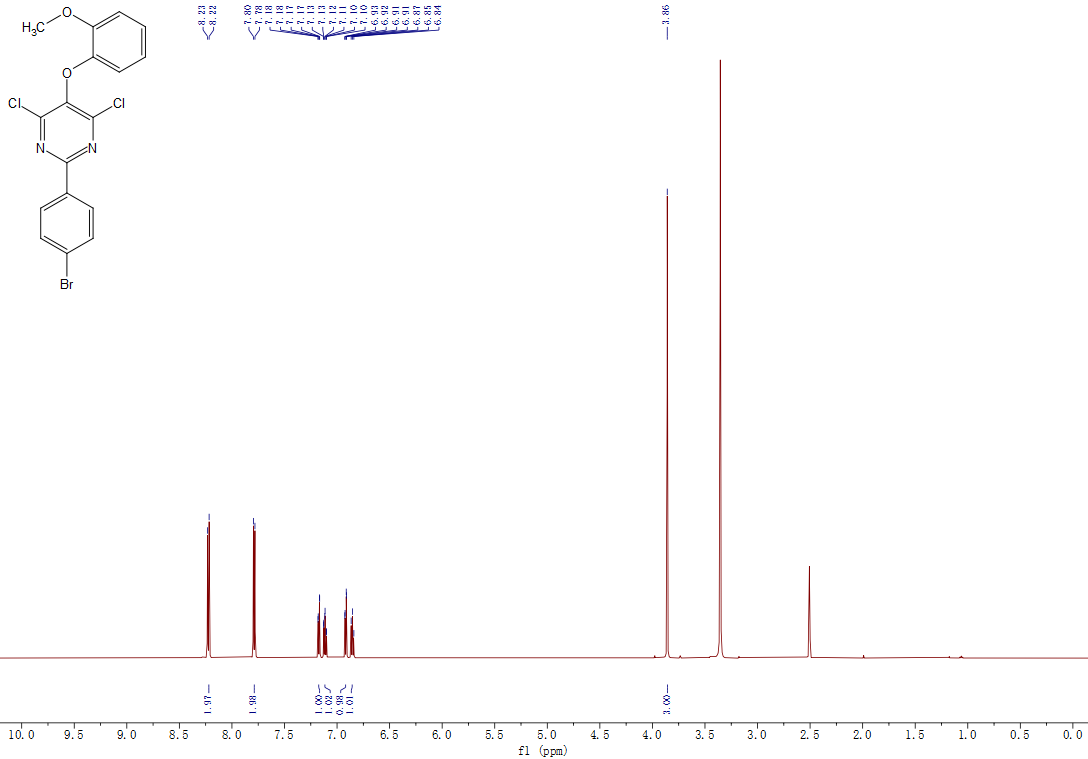


**Figure S40. ^1^H-NMR spectra of 7d**


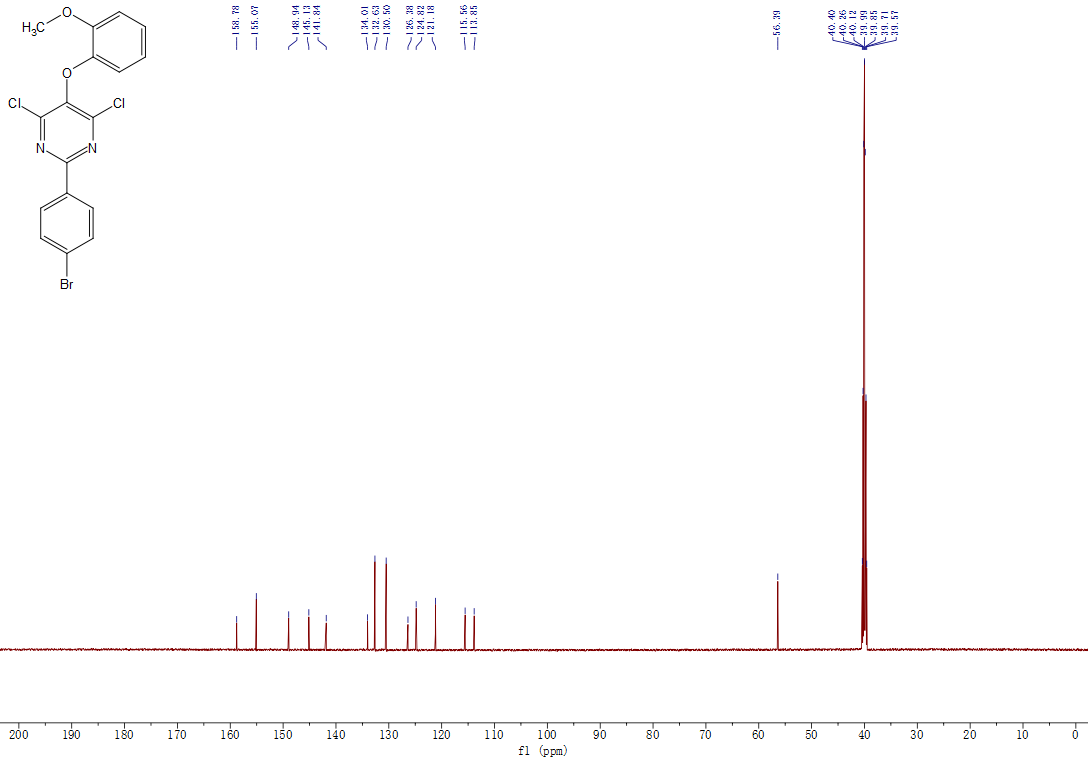


**Figure S41. ^13^C-NMR spectra of 7d**


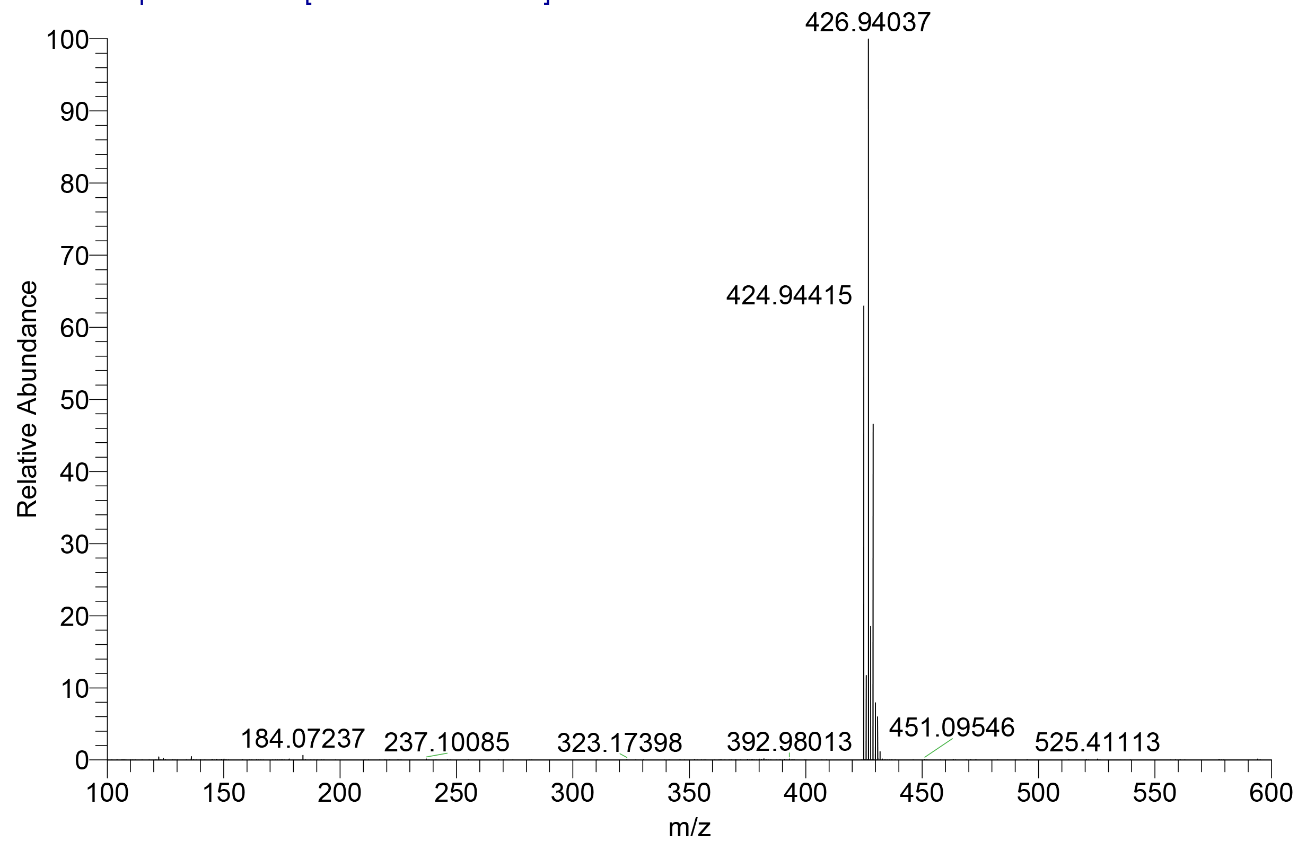


**Figure S42. Mass spectrum of 7d**

*
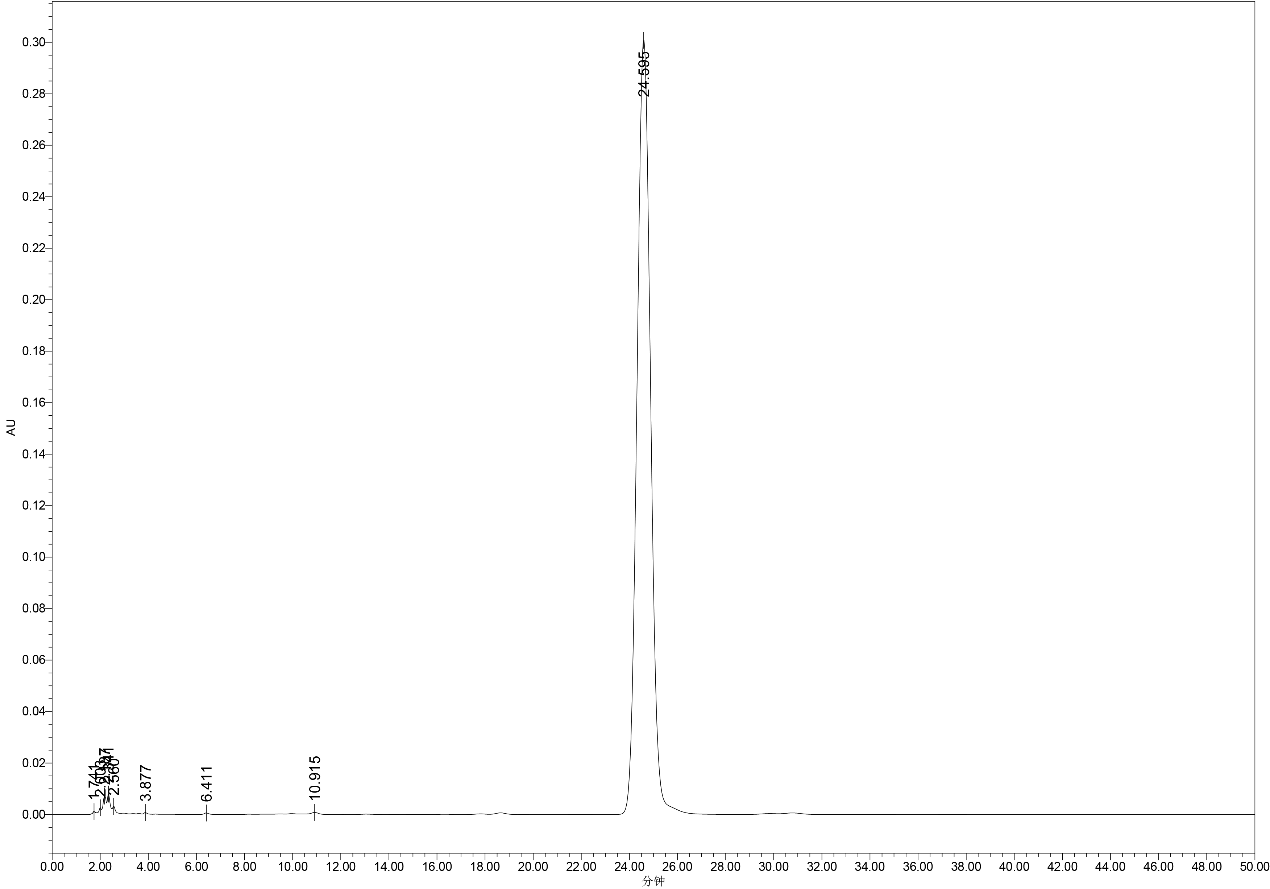
*

**Table S14.** HPLC analysis of **7d** (1.0mL/min, Acetonitrile: Water = 55:45 v/v).

| Peak | Retention time | Peak area | Peak area % | Peak start | Peak end |
| --- | --- | --- | --- | --- | --- |
| 1 | 1.741 | 9160 | 0.08 | 1.567 | 1.867 |
| 2 | 2.003 | 17717 | 0.15 | 1.867 | 2.067 |
| 3 | 2.187 | 59378 | 0.5 | 2.067 | 2.267 |
| 4 | 2.341 | 63161 | 0.53 | 2.267 | 2.483 |
| 5 | 2.56 | 23270 | 0.2 | 2.483 | 2.817 |
| 6 | 3.877 | 4487 | 0.04 | 3.75 | 4.05 |
| 7 | 6.411 | 5810 | 0.05 | 6.233 | 6.617 |
| 8 | 10.915 | 8934 | 0.07 | 10.7 | 11.183 |
| 9 | 2295 | 11722726 | 98.39 | 23.6 | 26.283 |

*2.15 4,6-dichloro-2-(3-iodophenyl)-5-(2-methoxyphenoxy)pyrimidine (****7e****)*


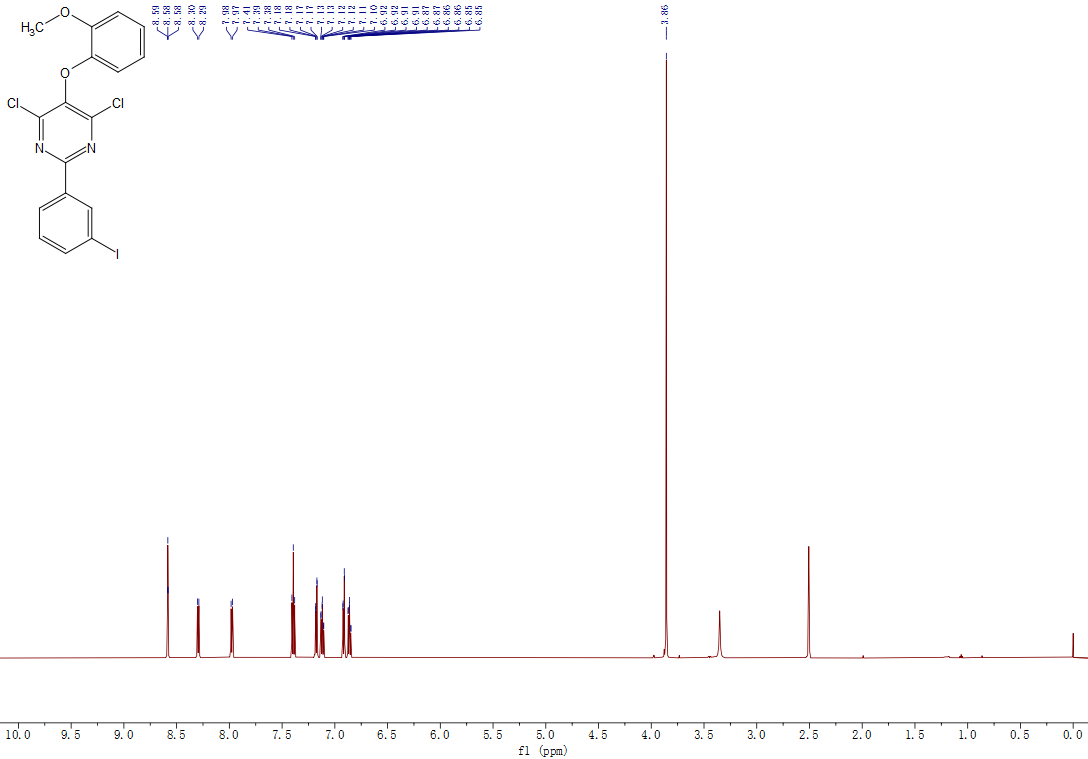


**Figure S43. ^1^H-NMR spectra of 7e**


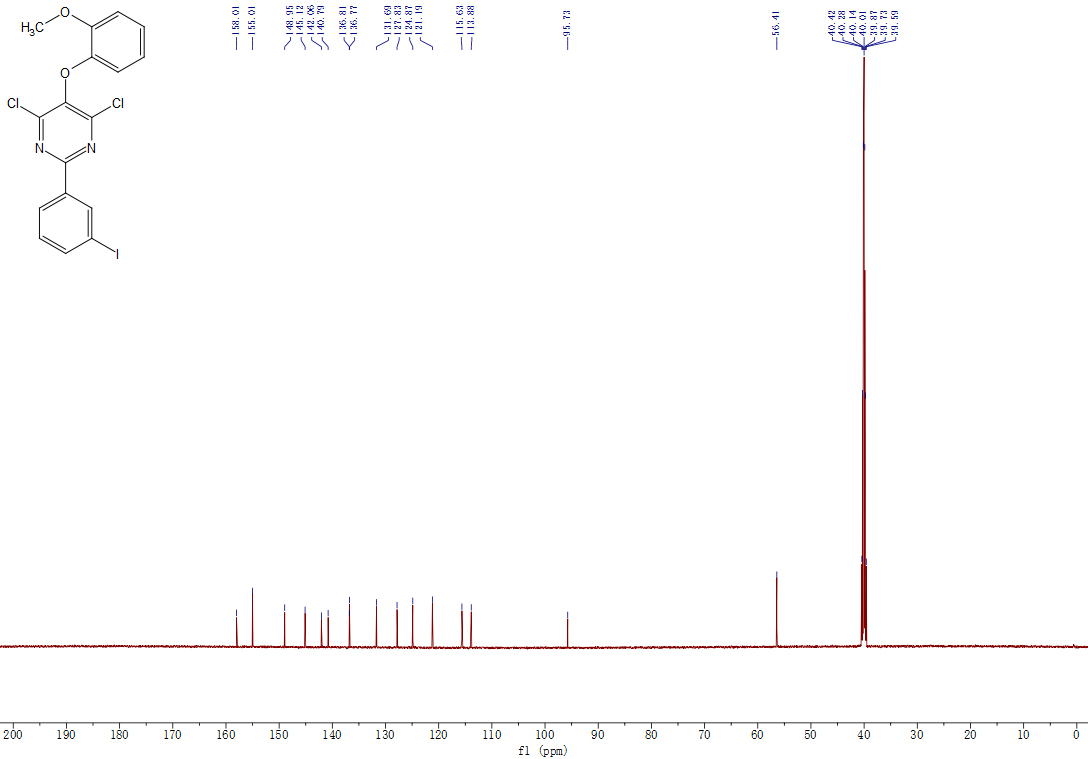


**Figure S44. ^13^C-NMR spectra of 7e**


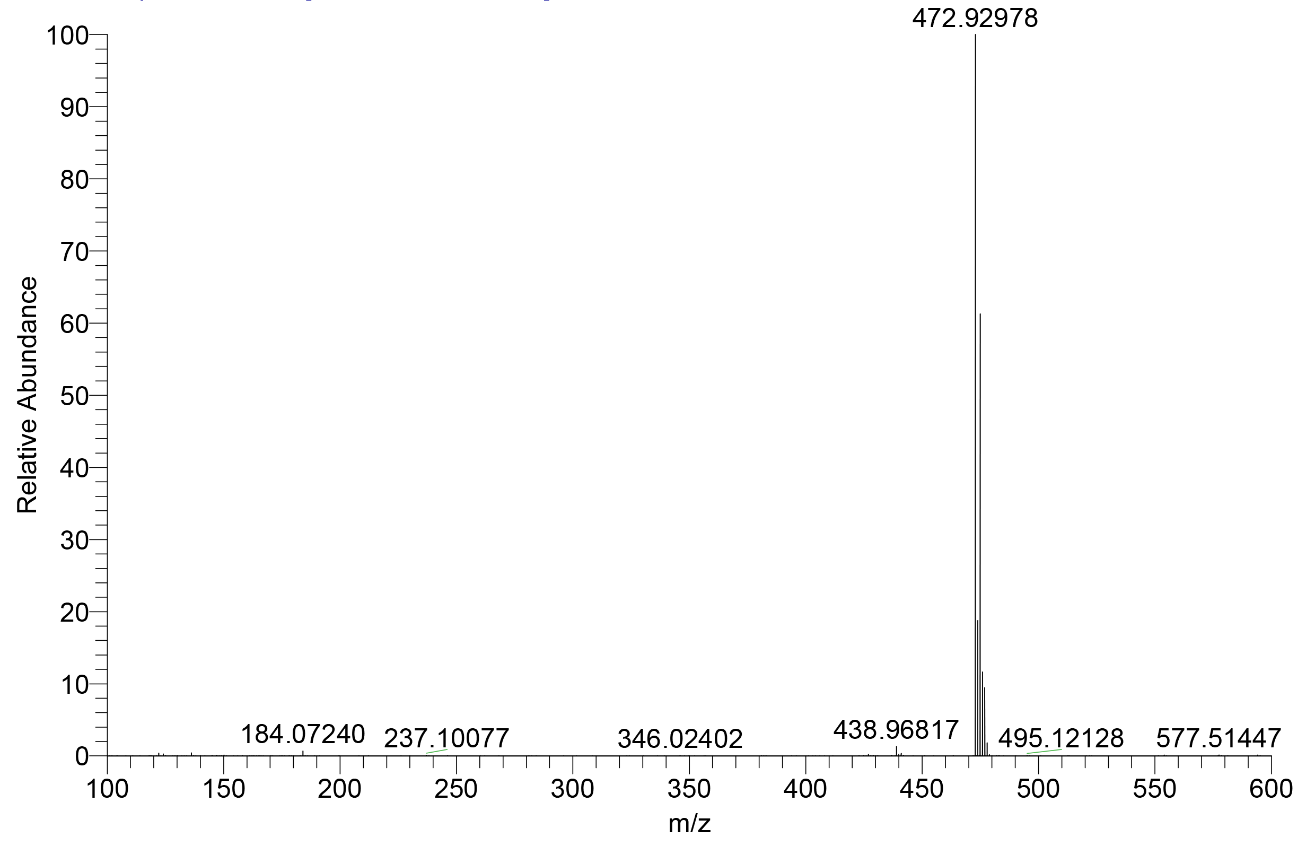


**Figure S45. Mass spectrum of 7e**

*
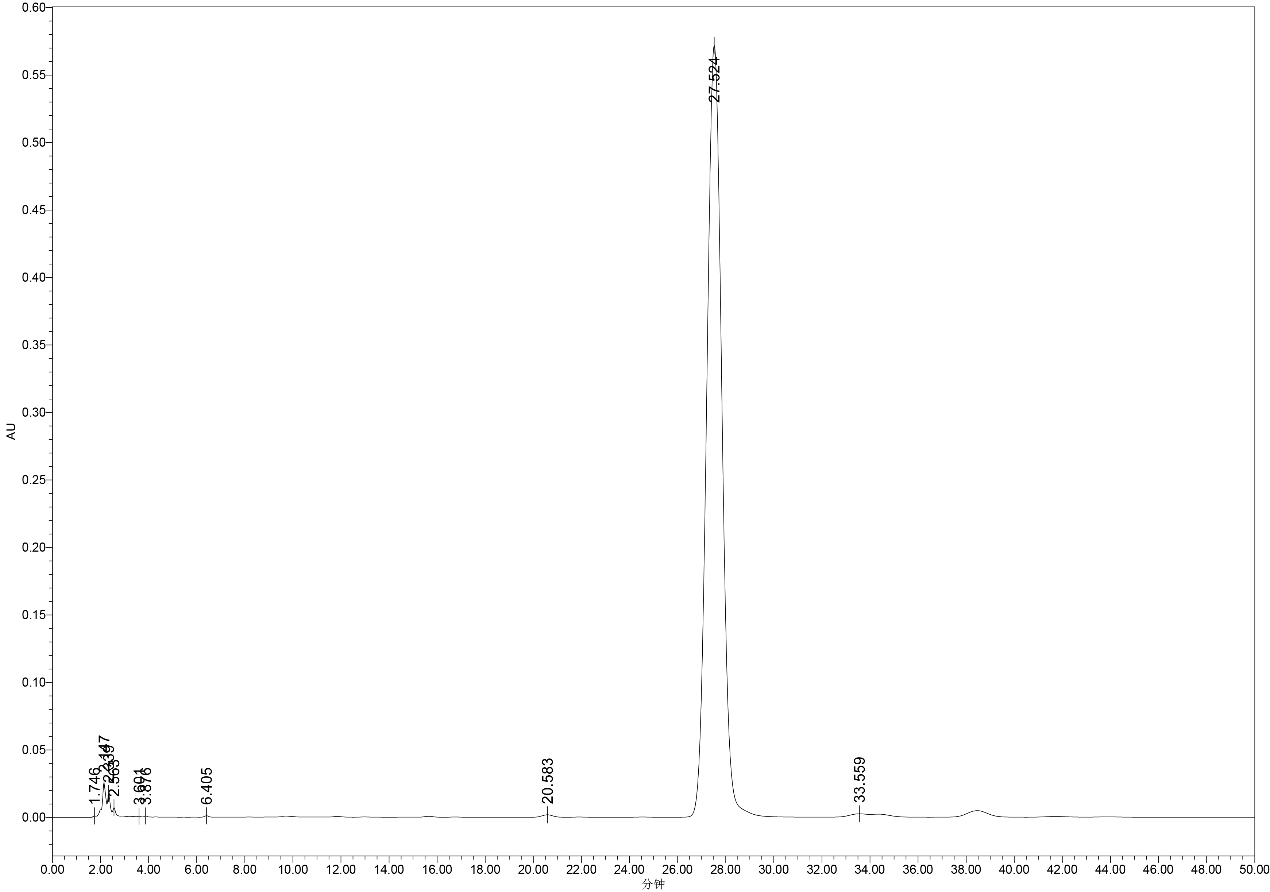
*

**Table S15.** HPLC analysis of **7e** (1.0mL/min, Acetonitrile: Water = 55:45 v/v).

| Peak | Retention time | Peak area | Peak area % | Peak start | Peak end |
| --- | --- | --- | --- | --- | --- |
| 1 | 1.746 | 8335 | 0.03 | 1.567 | 1.85 |
| 2 | 2.147 | 255531 | 1 | 1.85 | 2.267 |
| 3 | 2.339 | 135498 | 0.53 | 2.267 | 2.483 |
| 4 | 2.563 | 51668 | 0.2 | 2.483 | 2.85 |
| 5 | 3.601 | 3106 | 0.01 | 3.483 | 3.733 |
| 6 | 3.876 | 6032 | 0.02 | 3.75 | 4.083 |
| 7 | 6.405 | 11678 | 0.05 | 6.2 | 6.65 |
| 8 | 20.583 | 46933 | 0.18 | 20.133 | 21.05 |
| 9 | 27.524 | 24760922 | 97.38 | 26.367 | 29.45 |
| 10 | 33.559 | 147554 | 0.58 | 32.9 | 34.95 |

*2.16 4,6-dichloro-2-(4-iodophenyl)-5-(2-methoxyphenoxy)pyrimidine (****7f****)*


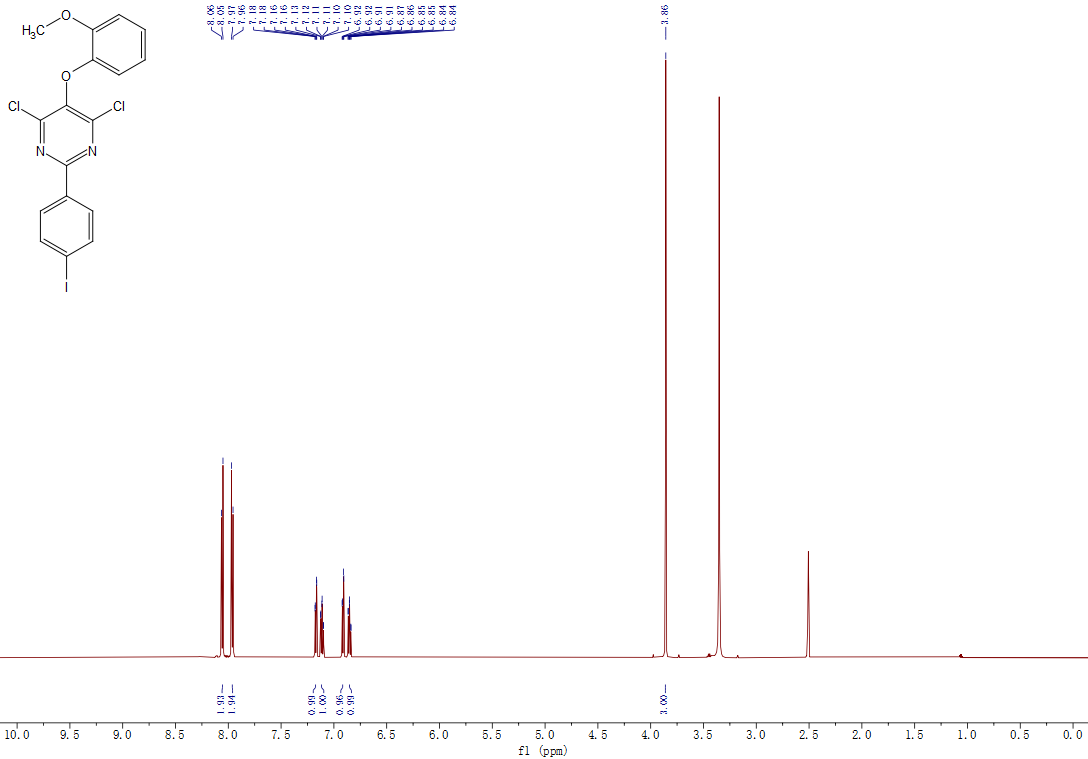


**Figure S46. ^1^H-NMR spectra of 7f**


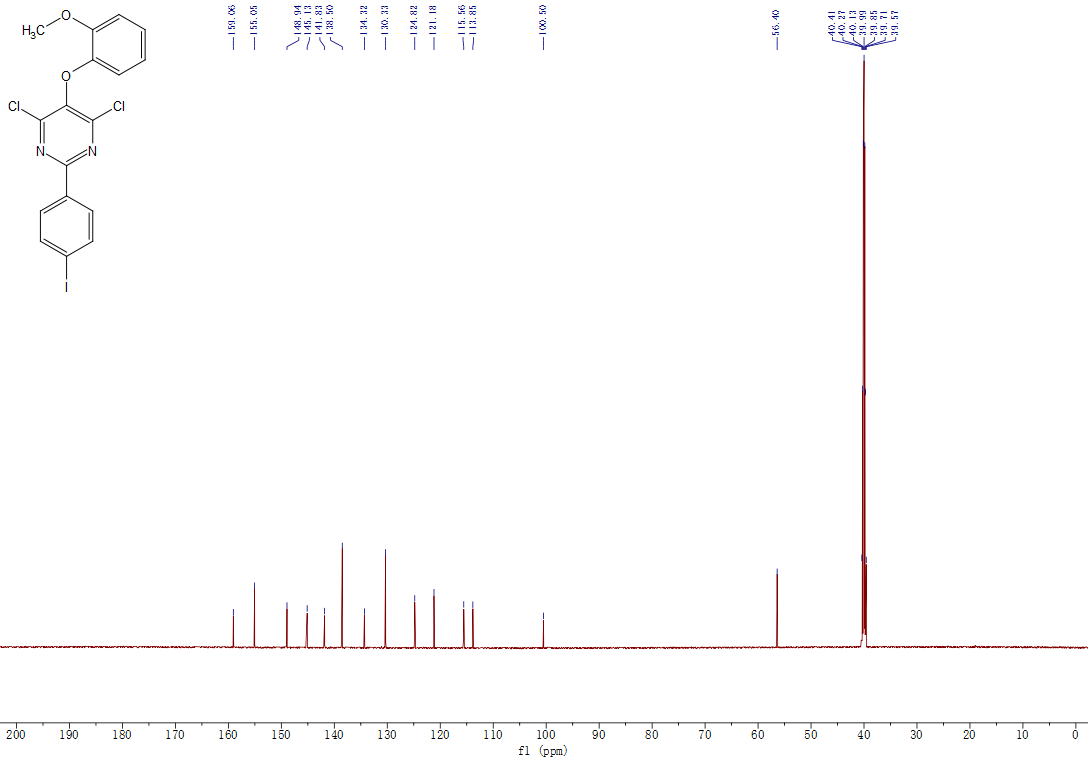


**Figure S47. ^13^C-NMR spectra of 7f**


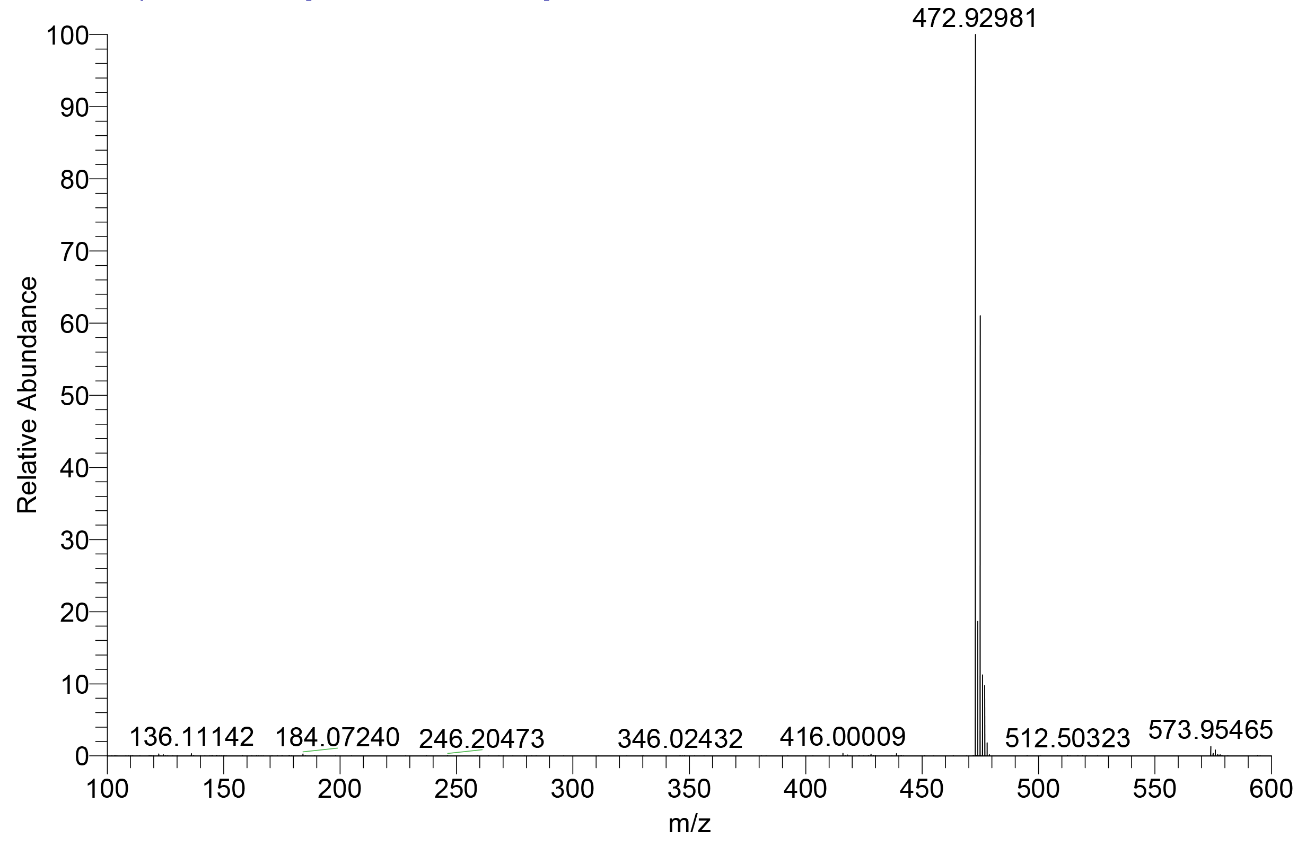


**Figure S48. Mass spectrum of 7f**

*
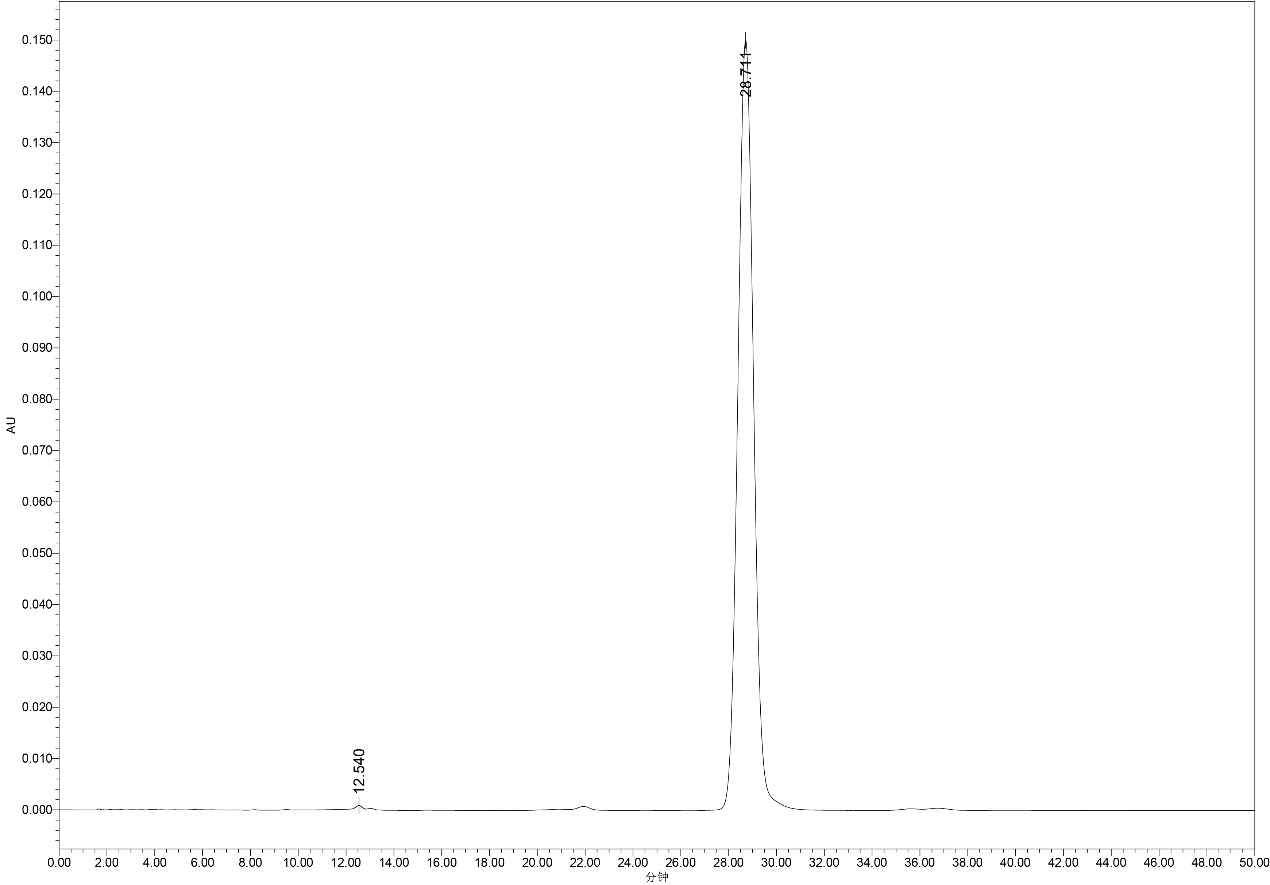
*

**Table S16.** HPLC analysis of **7f** (1.0mL/min, Acetonitrile: Water = 55:45 v/v).

| Peak | Retention time | Peak area | Peak area % | Peak start | Peak end |
| --- | --- | --- | --- | --- | --- |
| 1 | 12.54 | 10015 | 0.15 | 12.3 | 12.817 |
| 2 | 28.711 | 6705809 | 99.85 | 27.633 | 30.083 |

***3. Materials and methods***

*3.1 Tissue microarray*

Tissue microarrays were purchased from Shanghai Outdo Biotech Company (HMelC112CD01).

*3.2 Cell lines and treatments*

Melanoma cell lines—A375 cells, SK-MEL-28 cells, B16-F10 cells, and skin cell lines—HACAT cells were purchased from the American Type Culture Collection (ATCC, Manassas, VA, USA).

*3.3 mRNA-Seq and MeRIP-Seq*

The mRNA was separated from A375 cells using the Takara MiniBEST Universal RNA Extraction Kit (#9767, TAKARA) according to the manufacturer's instructions. The m6A immunoprecipitation (MeRIP) procedure was performed according to instructions issued by the manufacturer using a Magna MeRIP™ m6A kit (#17–10,499, Merck Millipore, MA). Briefly, purified mRNA was digested by DNase I and then fragmented into ∼100 nt using RNA fragmentation reagent and incubated at 94 °C. After fragmenting, the stop buffer was added, following which standard ethanol precipitation was performed and collected. The anti-m6A antibody for 12 μg was pre-incubated with 50 μL beads in IP buffer (150 mM NaCl, 0.1% NP-40, 10 mM Tris–HCl, pH 7.4) at room temperature for 1 h. Next, 6 μg of fragment mRNAs were added to the antibody-beads mixture and incubated at 4 °C for 4 h on a rotator. After adequate washing, the immunoprecipitated mixture was digested using high concentration of proteinase K, and the bound mRNAs were extracted using the phenol-chloroform method and ethanol precipitation and were used for qPCR analysis or library construction.

*3.4 RNA immunoprecipitation (RIP)*

General procedure of RNA immunoprecipitation (RIP) according to the manufacturer's instructions:5 μg anti-METTL3 (#15073-1-AP, Cell Signaling Technology), anti-eIF5A-Hypusine (RGK08101, AntibodySystem), and anti-rabbit IgG (Millipore, Germany) were incubated with 50 μL magnetic beads before cell lysates were added (approximately 2 × 10^7^ cells per sample). Then, the RNA-protein IP complexes were washed 6 times and proteinase K digestion buffer was used for incubation to remove the proteins. Finally, RNAs were extracted by phenol-chloroform RNA extraction and purified for PCR analysis. With the addition of primers, 2% agarose gel is used to detect the presence of bands of interest.

*3.5 Surface plasmon resonance (SPR)*

SPR procedure according to the manufacturer's instructions：First prepare a mixture of 1 μL of different concentrations of compounds (methanol as the solvent), 20 μM spermidine, 100 μM NAD, 50 μL glycine-NaOH buffer (pH 9.0), and 0.5 μg DHPS enzyme (Abcam, UK). The measured pH value was 8.0. Further, the mixture was incubated at 37^°^C for 2 h. Then 50 μL volume of the mixture to the light-proof 96 microtiter plate, an equal volume of NADH-Glo reagent, and incubated for 30 min at room temperature. Finally, used the BioTek Epoch full-wavelength microplate reader to record the luminescence readings. At the same time, a fresh NADH standard solution (0.5, 1, 2, 4, and 8 μM) was prepared in parallel for the NADH-Glo standard determination to build regression equations and estimated the generated NADH according to the standard curve, further calculated the inhibitory effect of target compounds on DHPS enzyme activity.

*3.6 Proteome Profiling*

Keratinocytes and ADSCs from 3 donors were harvested and solubilized in lysis buffer 17, according to the manufacture instructions (R&D Systems Inc., Minneapolis, MN, USA). Total protein concentration was determined using the DC protein assay (Bio-Rad Inc., Hercules, CA, USA), following the manufacturer’s instructions. Proteome Profiler™ Human XL Cytokine Array (R&D Systems, Inc., Minneapolis, MN, USA) was used to simultaneously assess soluble human proteins and their differential expression between the two cell types. Following the manufacturer’s instructions, the array membranes were blocked for 1 h on a rocking platform, and 150 µg of the cell lysates were incubated with array membranes overnight at 4 °C. The arrays were then incubated with a cocktail of biotinylated detection antibodies for 1 h followed by chemiluminescent detection with Streptavidin-HRP. Membranes were imaged using the ChemiDoc™ MP imaging system (Bio-Rad Inc., Hercules, CA, USA). The pixel densities at each capture spot were quantified and normalized to the reference spots of each blot. Images were analyzed using ImageJ 1.53c (Wayne Rasband National institutes of health, MD, USA) where the mean intensity corresponded to the relative expression of each blotted protein in the cell lysate.

*3.7 Tumor xenograft model*

Procedure of establishing tumor xenograft model:1 x 10^7^ A375 cells were subcutaneally implanted into nude mice with 100 μL PBS as the carrier, and the tumor volume reached 50 mm3 (tumor volume was calculated by the following formula :length×width2/2). Mice in each group (n=6) were intraperitoneally injected with 100 μL SPSS or 100 μL carrier (GL-1 or GC-7, 40 mg/kg) every 3 days. The tumor volume was monitored and measured with a caliper every three days. After 18 days of treatment, the mice were euthanized, xenograft tumors were dissected, and 0.5 mL of venous blood was collected with anticoagulant tubes.

*3.8 Pharmacokinetic studies*

Compound **GL-1** is prepared into the desired solution with DMSO + Solutol + SPSS for intravenous and intraperitoneal administration to 6 SPF male SD rats. Animals are fasted overnight (10 - 16 hours) prior to dosing. Blood samples are collected intravenously from each animal at 0.25 mL/time at 0.083, 0.25, 0.5, 1, 2, 4, 6, 8, and 24 hours after intravenous administration or at 0.083, 0.25, 0.5, 1, 2, 4, 6, 8, and 24 hours after intraperitoneal administration. Blood samples are placed in tubes containing sodium heparin and kept on ice until centrifugation. Blood samples will be centrifuged within 1 hour of collection at 2-8°C for 6 minutes at 6800 x g and stored frozen at approximately -70°C. Analytical results are confirmed using quality control samples for intra-assay variation. The precision of the >66.7% QC samples should be between 80% and 120% of the known value. Standard parameter sets including area under the curve (AUC_0-t_ and AUC_0-∞_), elimination half-life (T_1/2_), maximum plasma concentration (C_max_) and time to maximum plasma concentration (T_max_) were selected for data management using Microsoft Excel 2010 spreadsheet software and pharmacokinetic parameters were calculated using WinNolin 8.2 software.

***4. Additional results and related charts***

**Table S17.** The IC_50_ values of compounds **6a-7f** and GC-7 against DHPS, A375 cells, SK-MEL-28 cells, and CC_50_ values against HaCaT cells.

| **Compd. NO.** | **IC_50_ (μM) *^a^*** | | | | **CC_50_ (μM) *^a^*** |
| --- | --- | --- | --- | --- | --- |
|  | **DHPS** | **A375** | **SK-MEL-28** | **B16** | **HaCaT** |
| **6a** | - | >80 | >80 | >80 | - *^b^* |
| **6b** | - | - | - | 42.24±34.97 | >80 |
| **6c** | >80 | 6.15±0.84 | - | - | - |
| **6d** | - | >80 | 77.80±71.32 | - | - |
| **6e** | >80 | 7.10±0.36 | >80 | - | >80 |
| **6f** | - | 3.95±0.46 | 44.60±21.63 | - | - |
| **6g** | - | >80 | 40.16±33.72 | - | >80 |
| **6h** | - | >80 | - | >80 | 63.39±0.97 |
| **6i** | - | - | - | >80 | >80 |
| **6j** | >80 | >80 | - | 45.10±3.63 | - |
| **7a** | 4.21±0.51 | 25.15±1.44 | 22.89±5.05 | 23.23±5.38 | 29.97±0.41 |
| **7b** | 12.34±0.97 | 17.71±1.14 | 16.41±4.82 | 16.53±4.84 | >80 |
| **7c** | 16.81±1.42 | 17.50±0.39 | 16.17±1.59 | 16.29±1.65 | 33.48±0.77 |
| **7d** | 0.92±0.46 | 1.67±0.59 | 0.74±0.99 | 10.79±0.99 | 45.55±1.96 |
| **7e** | 2.67±0.66 | 2.21±0.39 | 6.28±0.48 | 6.33±0.48 | >80 |
| **7f(GL-1)** | 0.21±0.07 | 0.54±0.05 | 0.50±0.36 | 6.21±1.10 | 50.50±0.01 |
| **GC-7** | 1.58±0.02 | 2.26±0.39 | 3.73±0.19 | 8.38±0.24 | 17.63±0.27 |

*^a^* values were presented as means ± SD of at least three independent determinations.


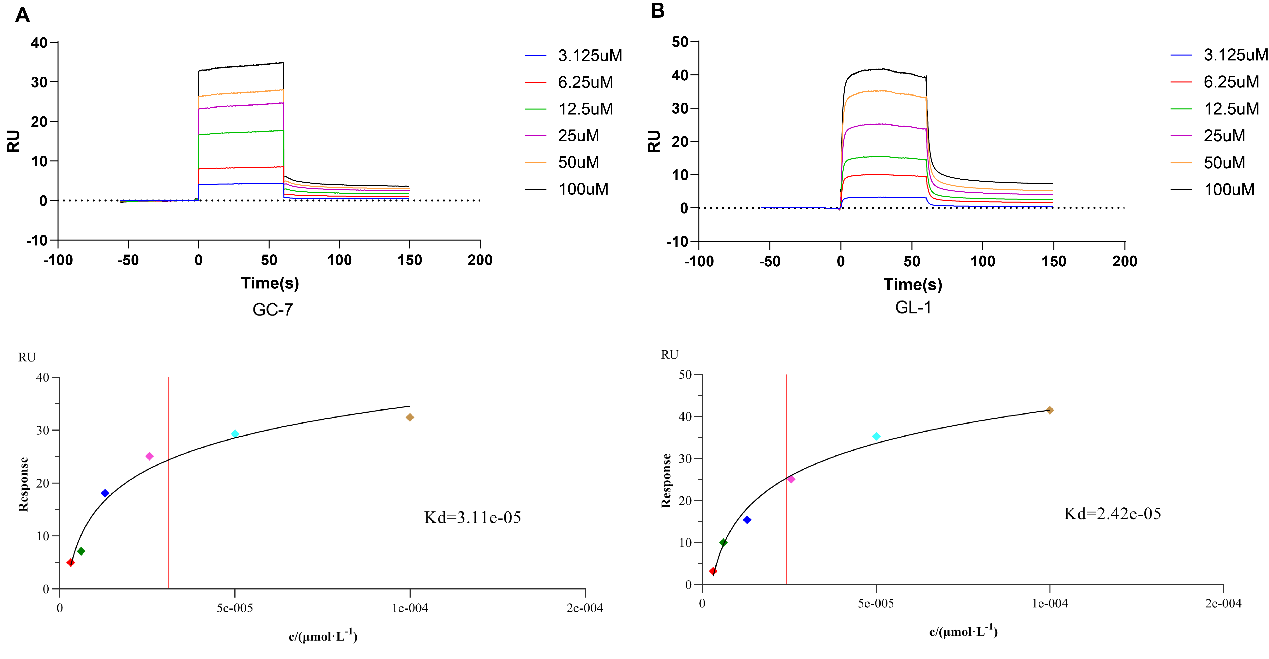


**Figure S49. SPR assay was used to analyze the affinity of GC-7 (A) or GL-1(B) for DHPS proteins.**


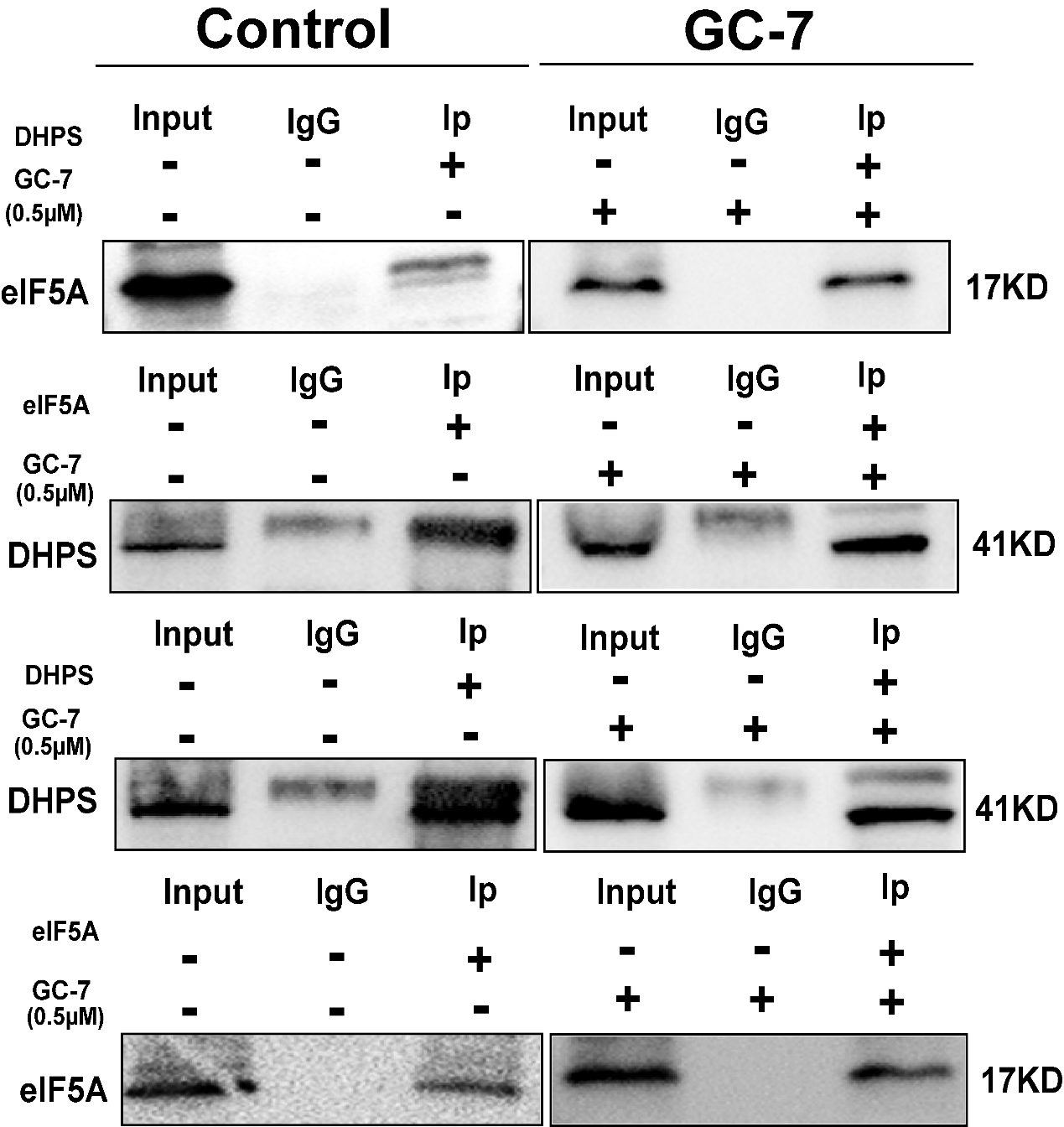


**Figure S50. Co-IP assay to probe the role of GC-7 on the interaction of DHPS with eIF5A protein.**


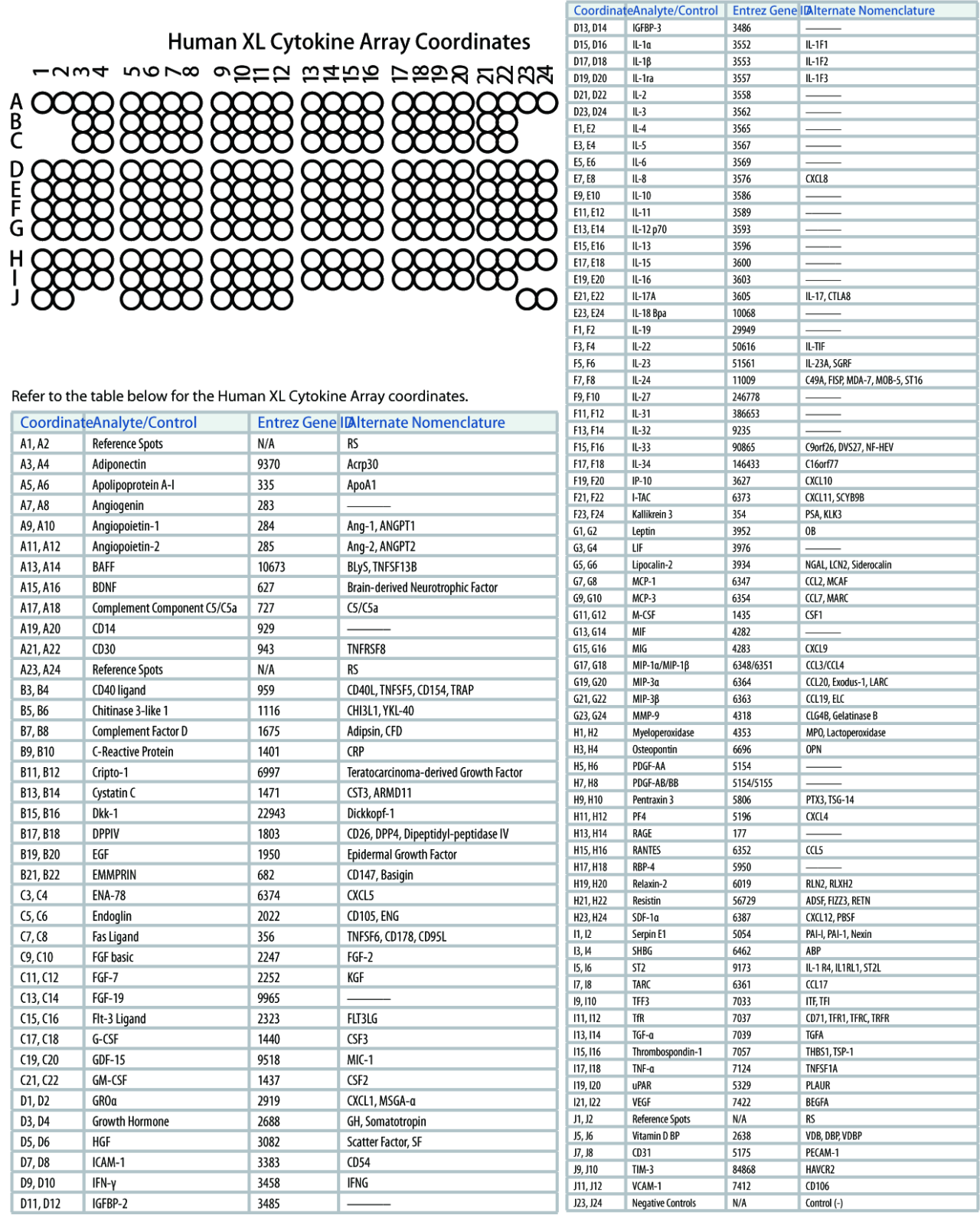


**Figure S51. Human XL Cytokine Array coordinates.**


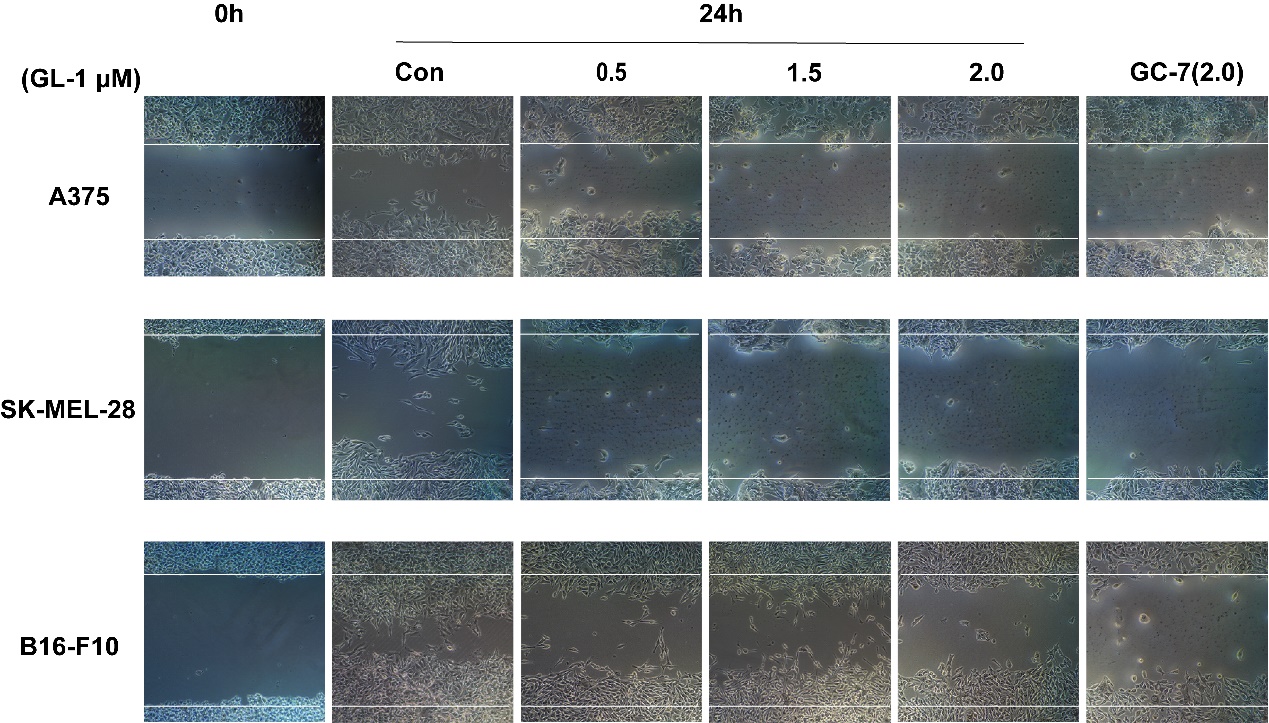


**Figure S52. Representative images of wound healing assay of A375, SK-MEL-28, B16-F10 cells after treatment with GL-1 and GC-7 drugs at 0 and 24 hours.**


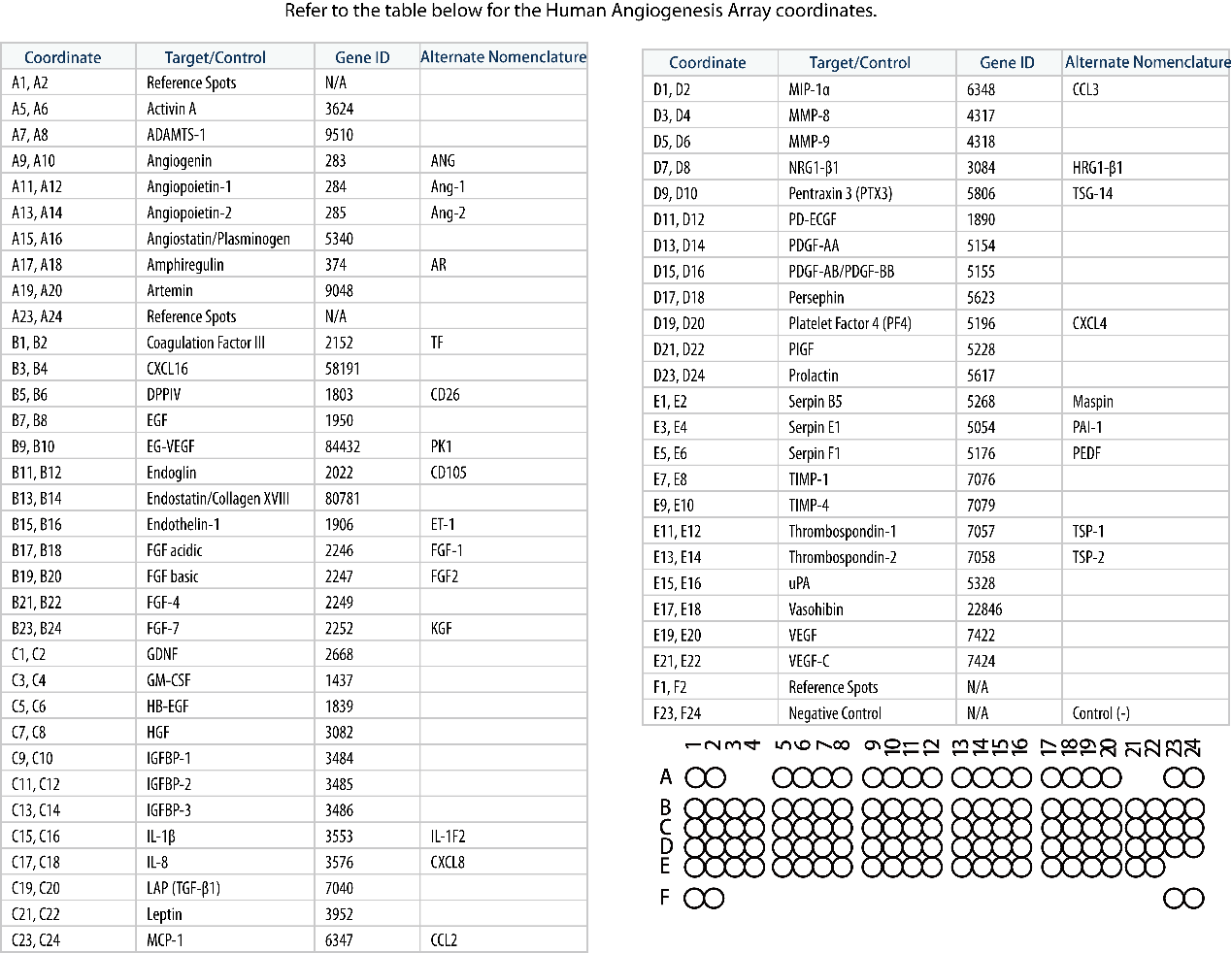


**Figure S53. Human Angiogenesis Array coordinates.**


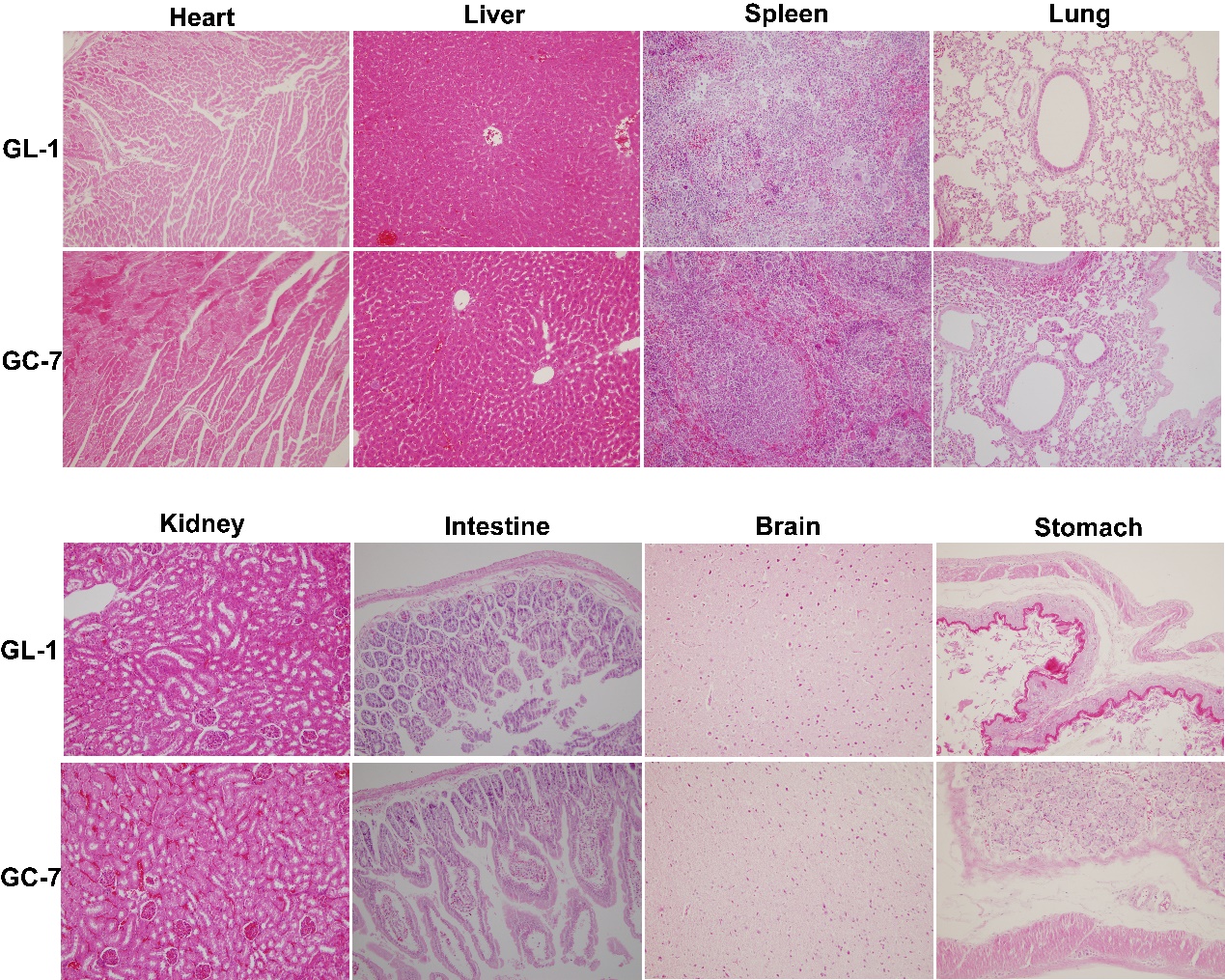


**Figure S54. Hematoxylin and eosin (HE) staining results of heart, liver, spleen, lung, kidney, intestine, brain, and stomach of nude mice after GL-1 and GC-7 administration.**

**Table S18.** The names and their Product numbers of the primary antibodies used in Western blot and co-immunoprecipitation (Co-IP) assays.

| **Primary antibody** | **Product number** | **Primary antibody** | **Product number** |
| --- | --- | --- | --- |
| GAPDH | 10494-1-AP, Proteintech Group | β-actin | 60008-1-lg, Proteintech Group |
| DHPS | ab224134, Abcam | TYMS | #15047-1-AP, Cell Signaling technology |
| Caspase3 | #19677-1-AP, Cell Signaling technology | Caspase9 | ab32539 |
| Cleaved-caspase3 | ab32042, Abcam | YTHDC1 | #14392-1-AP, Cell Signaling technology |
| YTHDF2 | #24744-1-AP, Cell Signaling technology | METTL3 | #15073-1-AP, Cell Signaling technology |
| eIF5A- hypusine | RGK08101,  AntibodySystm | eIF5A | #17069-1-AP, Cell Signaling technology |

**Table S19.** The names and their Product numbers of the primary antibodies involved in immunochemistry.

| **Primary antibody** | **Product number** | **Primary antibody** | **Product number** |
| --- | --- | --- | --- |
| METTL3 | #15073-1-AP,Cell Signaling Technology, USA | anti-Ki67 | b92742, Abcam, USA |
| YTHDC1 | #14392-1-AP, Cell Signaling Technology | anti-YTHDF2 | #24744-1-AP, Cell Signaling Technology |
| DHPS | ab224134, Abcam | anti-Cleaved-caspase3 | ab32042, Abcamtechnology |
| eIF5A-Hypusine | RGK08101, AntibodySystem technology |  |  |
